# Tinkering signaling pathways by gain and loss of protein isoforms: the case of the EDA pathway regulator EDARADD

**DOI:** 10.1186/s12862-015-0395-0

**Published:** 2015-07-02

**Authors:** Alexa Sadier, Elise Lambert, Pascale Chevret, Didier Décimo, Marie Sémon, Marie Tohmé, Florence Ruggiero, Théophile Ohlmann, Sophie Pantalacci, Vincent Laudet

**Affiliations:** Institut de Génomique Fonctionnelle de Lyon, UMR 5242 du CNRS, Université de Lyon, Université Claude Bernard Lyon 1, Ecole Normale Supérieure de Lyon, 46 Allée d’Italie, 69364 Lyon, Cedex 07, France; Laboratoire de Biométrie et Biologie Évolutive, CNRS UMR5558, Université de Lyon, Universite Claude Bernard Lyon 1, Villeurbanne, France; CIRI, International Center for Infectiology Research, Université de Lyon, INSERM U1111, Ecole Normale Supérieure de Lyon, Lyon, France

## Abstract

**Background:**

Only a handful of signaling pathways are major actors of development and responsible for both the conservation and the diversification of animal morphologies. To explain this twofold nature, gene duplication and enhancer evolution were predominantly put forth as tinkering mechanisms whereas the evolution of alternative isoforms has been, so far, overlooked. We investigate here the role of gain and loss of isoforms using *Edaradd*, a gene of the Ecodysplasin pathway, implicated in morphological evolution. A previous study had suggested a scenario of isoform gain and loss with an alternative isoform (A) newly gained in mammals but secondarily lost in mouse lineage.

**Results:**

For a comprehensive view of A and B Edaradd isoforms history during mammal evolution, we obtained sequences for both isoforms in representative mammals and performed *in vitro* translations to support functional predictions. We showed that the ancestral B isoform is well conserved, whereas the mammal-specific A isoform was lost at least 7 times independently in terminal lineages throughout mammal phylogeny. Then, to gain insights into the functional relevance of this evolutionary pattern, we compared the biological function of these isoforms: i) *In cellulo* promoter assays showed that they are transcribed from two alternative promoters, only B exhibiting feedback regulation. ii) RT-PCR in various tissues and ENCODE data suggested that B isoform is systematically expressed whereas A isoform showed a more tissue-specific expression. iii) Both isoforms activated the NF-κB pathway in an *in cellulo* reporter assay, albeit at different levels and with different dynamics since A isoform exhibited feedback regulation at the protein level. Finally, only B isoform could rescue a zebrafish *edaradd* knockdown.

**Conclusions:**

These results suggest that the newly evolved A isoform enables modulating EDA signaling in specific conditions and with different dynamics. We speculate that during mammal diversification, A isoform regulation may have evolved rapidly, accompanying and possibly supporting the diversity of ectodermal appendages, while B isoform may have ensured essential roles. This study makes the case to pay greater attention to mosaic loss of evolutionarily speaking “young” isoforms as an important mechanism underlying phenotypic diversity and not simply as a manifestation of neutral evolution.

**Electronic supplementary material:**

The online version of this article (doi:10.1186/s12862-015-0395-0) contains supplementary material, which is available to authorized users.

## Background

Explaining how species share a common genetic toolkit, but at the same time show a high phenotypic diversity is a crucial question of evo-devo [1–3]. Many examples described and theorized in the last 30 years have identified the genetic basis for evolutionary changes such as the modification of the existing toolkit by mutation (in coding or regulatory sequence) or its expansion/constriction by gene duplication and loss [1]. However, with the rise of omics, other mechanisms that contribute to diversification of developmental gene products can now be analyzed in depth. Among them, the generation of alternative isoforms is particularly relevant [4, 5].

Isoforms are generated by alternative splicing *sensu lato* (i.e. *via* differential exon retention, alternative promoters and alternative 3′ splice site) which, is considered as the most prominent mechanism for generating mRNA structural complexity [6, 7]. It can produce numerous new gene products that can be differentially regulated spatio-temporally and translated into proteins with different functions [8]. The evolution of alternative isoforms can give rise to new function or sub-function in different species [9]. Thus, the generation of alternative isoforms is now considered as an important force in evolution. Different levels of alternative splicing *sensu lato* were described among eukaryotes (and more precisely in the animal kingdom) [10–12]. Studies that compared transcriptomes between species or tissues have shown that a significant portion of splicing events are species-specific [5], suggesting a high turn-over in isoform gain and isoform loss and raising the question of whether the evolution of isoforms is largely neutral. However the extent to which this quantification is biased is unclear as datasets often incorporate unwanted splicing events like noisy splicing or regulatory splicing (e.g. the splicing of an alternative transcript does not result in encoding another protein but rather in regulating the abundance of another transcript [9]). Moreover, omics studies can only rely on very indirect and sometimes circular arguments (e.g. what is conserved is functional) to assess the functional significance of detected isoforms, if any and, the phylogenetic sampling is often very limited (e.g. mouse/human or a few mammals). In this context, such omics studies encounter difficulties concluding about the significance of patterns of isoform gain or loss during evolution. The field would now greatly benefit from specific case studies that combine functional experiments and broad phylogenetic sampling. Ideal candidates for such studies would be genes known to be important in phenotypic evolution, and that are likely to display variation in splice variants in a given group of species.

**Fig. 1:**
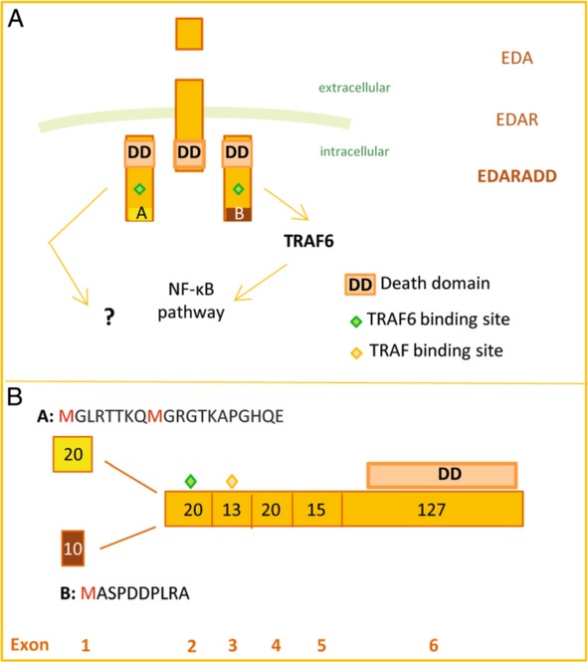
The EDA pathway and its downstream signaling. **a** EDA binds EDAR that recruits EDARADD B by interacting with its death domain (DD), leading to the downstream activation of the NF-κB pathway [17]. EDARADD A displays the same functional domains as EDARADD B but its activity is not known. Proteins are shown here with a color code that highlights exon 1A/B boundary. **b** The two isoforms differ by their short N-terminal part corresponding to the first exon that is close to the TRAF binding sites. Proteins are represented with exon boundaries and exon size (in amino acid) is indicated. Sequences of the short N-terminal peptide encoded by human exon 1A and 1B are represented in yellow (1A) and brown (1B)

*Edaradd* is one such promising candidate [9]. It encodes the intracellular scaffold protein EDARADD (or adaptor) specifically involved in the EDA-A1 pathway. In all vertebrate species tested so far, this pathway is necessary to the normal formation of ectodermal appendages (i.e. teeth, scales, hair, feathers, glands…) that are either missing or malformed in loss of function mutants [13, 14]. Since ectodermal appendages are hot spots of adaptation, this pathway is particularly relevant for morphological evolution [13]. It has been repeatedly found to be involved in morphological evolution, in species as distant as the stickleback fish and humans [15, 16]. Briefly, this pathway is related to the TNF receptor signaling pathway (Fig. 1A) and is composed by three specific components: the ligand EDA-A1, the receptor EDAR and the adaptor EDARADD. EDARADD serves as a relay between the receptor activated by the ligand and other intracellular proteins (such as TRAFs) involved in NF-κB pathway regulation, resulting in the activation of the NF-κB-dependent transcription upon ligand binding [17]. In a previous study we have shown that all three corresponding genes of the EDA-A1 pathway are highly conserved in vertebrates [18]. Most interestingly, in the context of this study, we had noted that, while non-mammalian genomes only carry a single isoform for the EDARADD adaptor, in mammals this gene encodes two isoforms, called A and B. The two isoforms differ by their short N-terminal part encoded by alternative first exons for detail on the genomic region see Additional file 1: Figure S1. The B isoform more closely resembles the isoform found in chicken, although the limited sampling associated with the short size of the B peptide did not allow drawing firm conclusions [18]. We also noted that B isoform is the only isoform found in mouse and rat [18]. These data therefore suggest a complex history of gain and loss with a newly evolved isoform in the mammalian lineage (A isoform), which was secondarily lost in the mouse/rat lineage.

Thus the Edaradd gene would provide a case study for the gain and loss of isoforms expressed from alternative promoters in relation to phenotypic evolution. It is quite obvious that isoform gain and evolution could support phenotypic evolution (and notably phenotypic innovation), provided that isoforms have different function or regulation. Because the functional tests to establish the function of EDARADD (mentioned above) were carried out in mouse, where the isoform A is absent, or in human, selecting the human-mouse conserved B isoform, it is currently unknown whether the two isoforms have a similar or a very different function. It is hard to predict just from their sequence. On one hand, in terms of domain content, both isoforms are very similar (Death domain, TRAF binding domains), but on the other hand, a very short N-terminal peptide is in principle sufficient to confer different biochemical properties, such as different levels of activation or different downstream signaling. Moreover, *in silico* predictions [18] had suggested that the two isoforms are likely expressed from two neighboring but different promoters. Therefore, besides their biochemical function, they could differ in terms of transcriptional regulation. Any of these differences could combine and translate in differential activation of the EDA pathway depending on the tissue and/or developmental time that could lead to phenotypic differences in terms of number and shape of ectodermal appendages. Based on published data, it is entirely possible that *Edaradd* isoforms differ in term of function and/or regulation, yet it remains to be proven.

When a new isoform is added to an old one during evolution, their ability to sustain phenotypic evolution in the long term might depend on the way the old and new isoform share the core gene function, that were ancestrally performed by the old isoform. Two extreme scenarios are possible: In the first, core functions are partitioned between both isoforms, in which case both may be strongly constrained. Alternatively, the ancestral isoform keeps core functions while the newly evolved isoform has more accessory roles, free to evolve in close association with phenotypic evolution. We expect that the gene will have a higher ability to support adaptive evolution (i.e. higher evolvability) in the latter case than in the former case, and in the former case than in absence of isoforms. Evaluating these possibilities for *Edaradd* not only requires a better knowledge of A and B isoform function, but firm conclusions about isoform B ancestrality and a broader view of A and B isoform evolution in mammals.

Therefore, in this study, we questioned the evolution of A and B isoform in a broader set of mammals than done previously [18], associating *in silico* predictions and bench experiments to firmly conclude about functionality. We showed that B isoform is ancestral and conserved, while A isoform exhibits an unexpected mosaic conservation in mammals, with many independent losses found during their late diversification. Moreover, we dissected the respective biochemical function and regulation of the two isoforms, in a set of experiments performed *in vitro*, *in cellulo* and *in vivo*. We conclude that the two isoforms mainly differentiated in term of regulation, keeping a similar function of NF-κB activation. We discuss how this combination of similar function and divergent regulation may enable differential regulation of the pathway in different developmental contexts, and we envision a scenario where the regulation of A isoform has been fast evolving in mammals in parallel with diversification of their ectodermal appendages, while B isoform was maintaining essential functions. We finally argue that isoforms generated from alternative promoters as exemplified by *Edaradd* could be an underappreciated mechanism ensuring the stability of some phenotypes and diversification of others during evolution.

## Results

### The B isoform of EDARADD is well conserved even outside mammals

In order to enlarge our previous dataset [18], the genomic region of the exon 1B was obtained by homology search (BLAST) against recently published genomes using the region of the closest available species as a probe. In previous work, we had suggested that the B isoform of EDARADD was homologous to the isoform found in chicken, suggesting that the B isoform could be the ancestral isoform whereas the A isoform would have been gained in mammals [18]. This finding was confirmed here by comparing sequences obtained from a larger panel of bird species (the extant vertebrate group closest to mammals). Indeed, both at the nucleotide and the amino-acid level, the N-terminal end of the mammalian B isoform exhibits strong similarities with the N-terminal end of the isoform found in birds [see Additional file 2: Figure S2]. In addition, the B isoform was found highly conserved in our upgraded set of 22 mammals [see Additional file 2: Figure S2]. More specifically, the promoter, the 5′UTR and the coding region of exon 1B was found in all genomes, except in some low coverage genomes for which the genome assembly is obviously incomplete in this region.

### The A isoform has been repeatedly lost during late mammal diversification

**Fig. 2:**
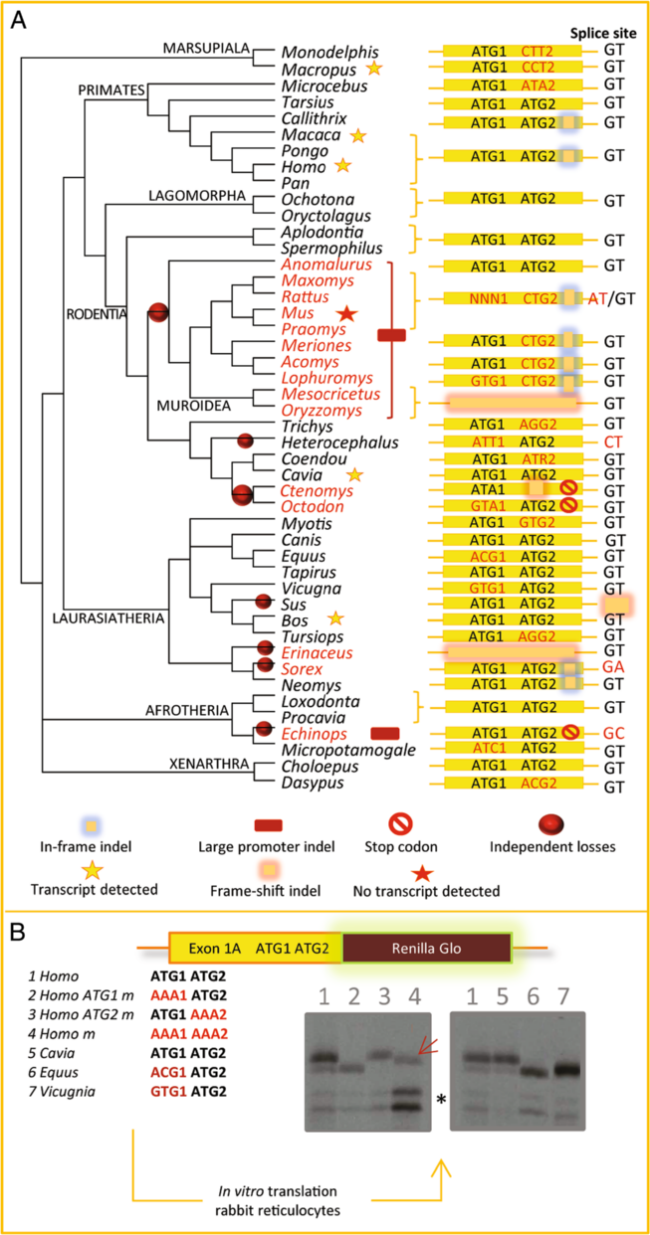
Mosaic conservation of the exon 1A in mammal evolution. **a** Examination of the exon 1A region in mammals. The yellow box represents the exon 1A in several mammal species [details in Additional file 6: dataset S6]. In human, two putative ATG could initiate the translation of a protein. For all species, these ATG are indicated as ATG1 and ATG2 when conserved. When not, the corresponding mutated codon is shown. The conservation of the promoter, the splice donor site and the frame were also studied and are indicated with symbols. The isoform-A transcript was detected by RT-PCR in human, guinea pig (*Cavia*) and wallaby (*Macropus*) or identified by BLAST against EST databases in macaca and cow (*Bos*). Species that are considered to have lost the ability to produce isoform A are shown in red. **b** *In vitro* translation experiments. The exon 1A of *Homo* [43], *Homo* mutated for the first ATG (2), *Homo* mutated for the second ATG [44], *Homo* mutated for both ATG [43] *Cavia* (5), *Equus* (6), *Vicugnia* (7) were cloned upstream the *Renilla* gene to test by *in vitro* translation their ability to produce a protein from the first exon with different ATG 1 and 2 context. The asterisk shows two faint bands that are the result of low efficiency initiation at a downstream non-specific ATG. The dark red arrow points out the faint band initiated by an alternative AAG codon for the human double mutant

The picture is different for the A isoform. In previous work, we had found that the exon 1A producing A isoform is conserved in a set of mammals but pseudogenized in mouse and rat [18]. This prompted us to increase our phylogenetic sampling by adding not only newly sequenced mammals (as done for B isoform) but also various rodent species, for which the region was obtained by PCR amplification followed by sequencing (Fig. 2A). We then aligned the putative promoter 1A region found ca. 500 bp upstream the ATG and looked for different criteria enabling functionality prediction, namely, the conservation of the promoter region, the splice donor site and the coding region with *bona fide* initiation site. In fact, in most species, exon 1A contains two ATG (here named ATG1 and ATG2) that can potentially initiate the translation: both are in frame and exhibit a canonical Kozak sequence, an indication of functionality.

However, a rapid look at this dataset revealed a very patchy conservation of ATG1 and ATG2: in some species (other than mouse and rat), ATG1 or ATG2 or both we mutated (Fig. 2A). Not only does such patchy conservation raise doubts as to whether a protein can actually be initiated in this exon 1A, but it is questionable whether a single ATG will be sufficient when the other is lost. Therefore, to be able to make realistic predictions from our dataset, we tested *in vitro* different constructs emanating from 4 different species where both ATG are present or one ATG is lost.

To this end, we cloned wild-type and mutant versions of the first exon of the A isoform (including the native 5′UTR region known to influence translation efficiency) upstream of the *Renilla* reporter gene and performed *in vitro* translation (Fig. 2B). For the wild type human construct (lane 1), we detected two bands that are alternatively lost for the single ATG mutant constructs (lanes 2 and 3) and both lost in the double mutant construct (lane 4). This supports the view that the first (strong) band corresponds to initiation at ATG1 and the second (faint) band to a less efficient initiation at ATG2. In such a test, it is common to observe that the presence of a potent ATG strongly limits initiation at downstream or nonspecific initiation sites, as ribosomes will preferentially initiate at the first site that they encounter during ribosomal scanning. However, if the most 5′ ATG is mutated, then initiation can occur with a higher frequency at these other sites. This also explains why other bands are observed in the double mutant (lane 4). Indeed, we observed two strong much lower bands (asterix, lane 4) that likely correspond to initiation at downstream sites, found within the renilla sequence (note that these two same bands are accordingly also hardly detected with all other constructs: see lanes 1 to 7). We also observed one faint band (lane 4, dark red arrow), which is slightly lower than the one seen with the wild type construct (compare lane 4 with lanes 1 and 3). Here, an analysis of the sequence showed the presence of a “near cognate AAU” site (an AAG codon), which is located 3 codons downstream from the ATG1 and was likely recognized by ribosomes, producing this faint band. With the guinea pig construct containing both ATG1 and ATG2, we obtained a similar pattern of initiation as with the wild type human construct (lane 5). Finally, in two species where only the ATG2 remains (*Equus* and *Vicugna*), initiation efficiently occurred at this site, as it is the case with the human ATG1 mutant construct (lane 6 and 7). We concluded that ATG1, or ATG2 (when ATG1 is missing), are potent initiation sites. As a consequence, we then infer in Fig. 2A that the A isoform is lost only when the two ATGs are mutated and/or the promoter region or the splice donor is mutated or absent (species indicated in red in Fig. 2A).

Our analysis revealed that in muroid rodents, the promoter region is completely rearranged: we observed a deletion of the conserved region where the transcriptional start site is mapped in human, replaced by a 72 bp insertion (see the red brace encompassing muroid rodents and the red box symbolizing the deletion in Fig. 2A). Mosaic deleterious events are observed in the coding sequence (ATG1 and/or ATG2 lost, frame-shift indels). This pattern strongly suggests that a deletion of the promoter region occurred in the common ancestor of muroids and abrogated the transcription of exon 1A (as observed in mouse, where the pseudogenized exon 1A is not transcribed, [18]). Following this event, constraints on the coding sequence were relaxed, leading to its mosaic degeneration in muroid rodents.

In other mammals, we found other losses (Fig. 2A). In *Ctenomys* and *Octodon*, two rodents that are more closely related to guinea pig where the A isoform is expressed [18], frame-shift indel and stop codons are observed. In *Echinops*, the promoter region exhibits a deletion and a stop codon is found in the coding sequence. The whole region is lost in *Erinaceus* and the splice site is lost in *Sus* as well as in *Sorex*. Because these species belong to very different branches of the mammalian tree or the mutation events are obviously independent, we could map at least 7 independent losses (red points in Fig. 2A). It is noteworthy that isoform-A is found in marsupials and in all main groups of placental mammals. Losses occurred later, during the diversification of these groups, as exemplified in rodents. In the next sections, we compared the function and regulation of A and B isoforms.

### The two EDARADD isoforms are expressed by two alternative promoters that are differentially regulated

**Fig. 3:**
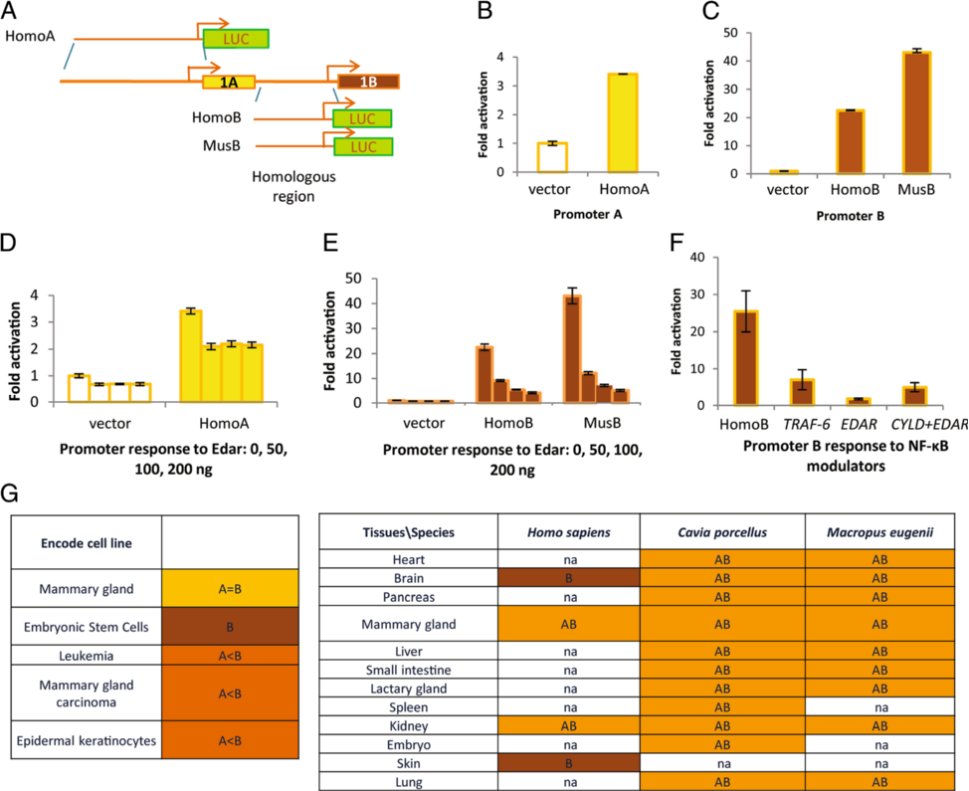
The two isoforms of EDARADD are expressed by two differentially regulated alternative promoters. **a** Scheme of the promoter constructs. The human promoter A region has no homologous region in mouse. Homo, *Homo sapiens*; Mus, *Mus musculus*. **b** *In cellulo* test of promoters activity. Constructs shown in A were transfected in HEK cells. Error bars show variation between triplicates of one out of 3 experiments. Y axis: normalized luciferase activity against β-galactosidase activity. **c** *In cellulo* test of promoters activity. B constructs shown in B were transfected in HEK cells. Three independent experiments were performed and yielded similar results. One out of these three experiments is shown with error bars showing variation between triplicates within this given experiment. Y axis: normalized luciferase activity against β-galactosidase activity. **d** Promoters response to EDA pathway activation by *EDAR* co-transfection. Constructs shown in A were co-transfected with increasing amount of an *EDAR* expressing plasmid: 0, 50, 100 or 200 ng. Error bars show variation between triplicates in one out of 3 experiments that yielded similar results. Y axis: normalized luciferase activity against β-galactosidase activity. **e** Promoters response to EDA pathway activation by *EDAR* co-transfection. B constructs were co-transfected with increasing amount of an *EDAR* expressing plasmid: 0, 50, 100 or 200 ng. Error bars show variation between triplicates in one out of 3 experiments that yielded similar results. Y axis: normalized luciferase activity against β-galactosidase activity. **f** Promoter B response to different NF-κB modulators. The human B promoter was co-transfected with 200 ng of *TRAF 6*, 200 ng of *EDAR* only or 200 ng of *EDAR* and 200 ng of *CYLD* supplemented or not by empty vector. Y axe: normalized luciferase activity as in B. **g** Expression of the two *EDARADD* isoforms. Expression of the two isoforms were assayed by RT-PCR in different tissues in human (*Homo sapiens*), a rodent guinea pig (*Cavia porcellus*) and a marsupial tammar wallaby (*Macropus eugenii*) (AB: A and B detected; B: only B detected; na: not assessed) and in different human cell lines using ENCODE RNAseq data (A = B : A and B co-expressed at similar levels; B > A: B expressed at higher levels; B: only B is detected). Cell lines: GM92, NHEK, GM78, H1ES, K562, MCF7, GM91

The existence of two distinct Edaradd mRNA [18] differing by their first exon suggests that two alternative promoters could control the expression of the two isoforms. To gain insight into the possible proximal regulation of the expression of both isoforms, we cloned the 5′ regions of the two putative promoters from two representative mammalian species upstream of a luciferase reporter gene and tested them in HEK cells that expressed both isoforms ([19] and data not shown). We chose the human gene which has the two isoforms, and the mouse which has lost the A isoform (Fig. 3A). When transfected in HEK cells, the B promoter exhibited a strong activity that was conserved in human and mouse (Fig. 3C). In contrast, the A promoter exhibited a low but significant activity (Fig. 3B). Because signaling pathways often feedback either positively or negatively [2], we tested promoter response to the EDA pathway activation by co-transfecting *EDAR* that induces an over-activation of the pathway *in cellulo* [20] (Fig. 3D and E). Whereas the A promoter did not exhibit any variation of its activity compared to the empty vector, the B promoter was downregulated in a dose-dependent manner by growing amount of transfected *EDAR*. This downregulation was observed with other NF-κB activators such as TRAF6 and partially rescued by NF-κB downregulators such as CYLD (Fig. 3F). It has to be noted that no cell death was detected during experiments (data not shown). Taken together, these results show a different regulation of the two promoters depending on the cellular context. To further investigate differences in transcriptional regulation *in vivo*, we studied the expression of the two isoforms in different tissues from two placental mammals (human, guinea pig - *Cavia porcellus* in Fig. 2A) and a marsupial (tammar wallaby, *Macropus eugenii* in Fig. 2A) by RT-PCR experiments and in human cell lines using the ENCODE data (Fig. 3G). The two isoforms were generally co-expressed in tissues and cell lines except in human skin and brain and in embryonic cell lines in which only the B isoform was detected. When co-expressed, the two isoforms were either expressed at the same level or the B isoform was more strongly expressed than the A isoform. This shows that the transcripts of A and B isoforms are differentially regulated *in vivo* and *in cellulo* in line with the differential regulation of their promoters in luciferase assays.

### The human A isoform of EDARADD is more active than the B isoform but accumulates more slowly

**Fig. 4:**
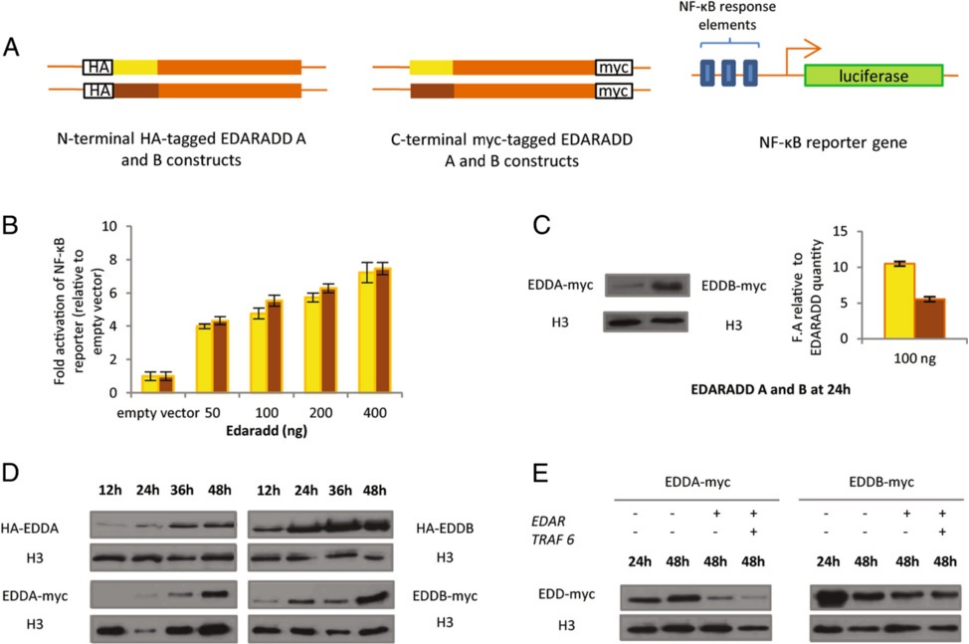
The two isoforms of EDARADD exhibit different activities and stabilities *in cellulo*. **a** Constructs. HA constructs: HA tag was N-terminally fused to EDARADD A or B protein. Myc constructs: Myc tag was C-terminally fused to EDARADD A or B protein. These constructs were generated using recombination so that 1A and 1B constructs only differ for A and B peptide coding region. NF-κB reporter gene: NF-κB are located upstream a minimal promoter and a luciferase reporter gene. **b** EDARADD A and B activate the NF-κB pathway in a dose-dependent manner. Increasing amounts of constructs encoding A or B isoforms were transfected in HEK cells with NF-κB reporter plasmid that harbors NF-κB response elements upstream luciferase reporter gene. The luciferase activity was normalized against β-gal and the empty vector. The luciferase activity was assayed 24 hours after transfection. In yellow: isoform A, in brown: isoform B. **c** A isoform is twice more active than B isoform when luciferase activity is corrected for Edaradd quantity. HEK cells were transfected with constructs encoding EDARADD A or B C-terminally tagged with myc (EDD-myc A or B) and the luciferase reporter gene as in A. 24 h after transfection, three wells were pooled in order to have enough material for Western Blotting and three others were used for luciferase assays. Proteins were detected by Western Blot with an anti-Myc antibody. Histone H3 was used as a control. Luciferase activity corrected for edaradd quantity is presented on the right. **d** EDARADD A and B reach different steady state levels. HEK cells were transfected with constructs encoding EDARADD A or B N-terminally tagged with HA (HA-EDD A or B) or C-terminally tagged with myc (EDD-myc A or B). Proteins were detected by Western Blot 12 h, 24 h, 36 h and 48 h after transfection. Histone H3 was used as a control. **e** EDARADD A but not B is destabilized by NF-κB activators. Western Blot for Myc C-terminally-tagged A and B isoforms following co-transfection of NF-κB activators such as *EDAR* or *TRAF6*, at 24 or 48 h. Histone H3 is used as a control

To decipher the biochemical role of the two EDARADD isoforms, we studied the function of the two human proteins *in cellulo* (Fig. 4) using the same HEK cells. We used recombination methods to generate expression plasmids for the two proteins that are rigorously identical (Fig. 4A), except for the small N-terminal peptide characterizing the A or B isoform (notably, translation initiation sites were identical). Since the first known function of the EDA pathway is to activate the NF-κB pathway we tested the ability of myc tagged-versions of both isoforms to activate the NF-κB pathway in a NF-κB reporter luciferase assay. As previously described, the B isoform activated the NF-κB pathway reporter in a dose-dependent manner [19] (Fig. 4B). The A isoform also activated the NF-κB pathway reporter in a dose-dependent manner. Examination of the protein level by western blot showed that the amount of B isoform was 2.5 higher than the amount of A isoform (Fig. 4C and D). Similar results were obtained whenever an N-terminal or a C-terminal tagged version of the proteins was used. Thus, to compare the activity of both isoforms, the luciferase activity needed to be corrected by the amount of EDARADD protein detected in the lysates. In so doing, the A isoform appeared to be twice more active than the B isoform (Fig. 4C). The co-transfection of the two constructs together at different concentrations did not exhibit any synergistic nor antagonistic effect [see Additional file 3: Figure S3]. We then followed the accumulation of the two proteins for 48 hours and found that the B isoform accumulated more rapidly than the A isoform (Fig. 4D). Considering the steady-state level of the two proteins, at 48 hours, the A isoform disappeared when co-transfected with *EDAR* or other NF-κB activators (Fig. 4E) whereas the B isoform remained stable suggesting a difference of regulation at the protein level. Similar results were obtained in presence of a proteasome inhibitor (MG132), presumably ruling out proteasome-dependent degradation (data not shown). In summary, the two human isoforms of EDARADD exhibit different functionalities *in cellulo*: they both activate the NF-κB pathway but exhibit differences in their dynamics: the B isoform accumulates more rapidly whereas the A isoform is more active but downregulated at the protein level following pathway activation. Such differences could impact their *in vivo* functions in transducing the NF-κB pathway both quantitatively and qualitatively.

### The two human isoforms of EDARADD exhibit different activities in a rescue experiment performed in zebrafish

**Fig. 5:**
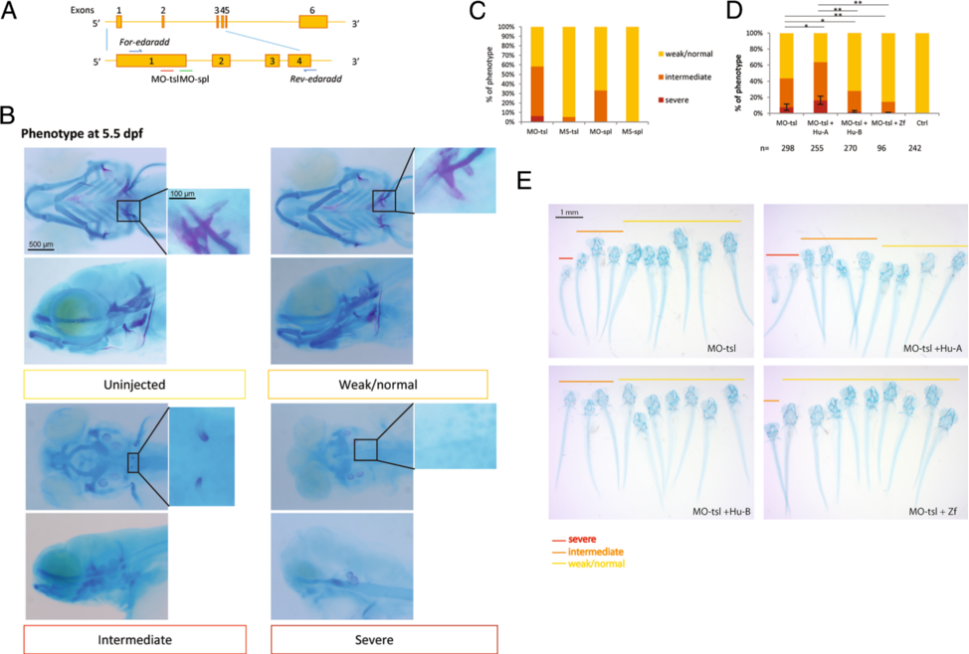
Human EDARADD B but not EDARADD A rescues skeletal defects observed in MO-injected zebrafish larvae. **a** Schematic representation of the exon-intron structure of the zebrafish *edaradd* gene. The location of target sequences for translation-blocking (MO-tsl) and splice-blocking (MO-spl) *edaradd* morpholinos is indicated by a red and a green line, respectively. Blue arrows indicate the location of the primers used for *edaradd* mRNA expression analysis during development [see Additional file 4: Figure S4] and for splice-blocking *edaradd* MO validation by RT-PCR analysis [see Additional file 4: Figure S4]. **b** Knockdown of Edaradd expression leads to skeletal defects. WT embryos were injected at one-cell stage and skeletal morphogenesis was analyzed through alcian blue and alizarin red staining at 5.5 dpf. The larvae phenotypes observed were classified as severe, intermediate or weak/normal phenotype. The severe phenotype presented no pharyngeal skeleton, a rudimentary neurocranial cartilage and no tooth could be detected. This defect was also accompanied by pericardial and yolk sac edema. Intermediate phenotype showed rudimentary pharyngeal skeleton without second arch and gill arch derivatives and sometimes one rudimentary tooth on each side of the larvae. Weak/normal phenotype demonstrated small gill arch derivatives or no obvious defects and at least one tooth on each side of the larvae could be clearly observed through alizarin red staining. Ventral and lateral views of the head are shown at low magnification (scale bar: 500 μm) and a zoom on the teeth is shown on the right panel (scale bar: 100 μm). **c** Percentages of the various phenotypic groups obtained following MO injection [see Additional file 4: Figure S4] or MS (Mismatch control) injection. These results are representative of at least 3 independent experiments following injection of at least 70 embryos per condition. **d** Human *EDARADD* A and B mRNA effects in rescuing experiments. One-cell stage embryos were co-injected with MO-tsl and/or capped human *EDARADD A* or *B*, or capped zebrafish *edaradd* mRNA with at least 70 embryos injected per condition. The barplot shows the percentage of each phenotypic group in 4 separate experiments and the statistical analyses have been performed on the severe group. **e** A representative group of 10 larvae, classified according to their phenotype, are shown for each condition

We next used the zebrafish system to assess if the two isoforms exhibited functional differences. To this end, we performed a rescue experiment where endogenous *edaradd* function has been obliterated by morpholino injections but is complemented by isoform A or B mRNA injections (Fig. 5). It is known that alterations of EDA pathway in zebrafish result in abnormal tooth and dermal skeleton development [21], but little is known about *edaradd* during early zebrafish development, i.e. the developmental window mainly targeted by morpholino injections.

Therefore, to help the interpretation of these experiments, we first gathered expression data for the endogenous unique splice variant of *edaradd* gene during zebrafish development. By semi-quantitative RT-PCR, we observed a high maternal expression of *edaradd*, which rapidly decreased at 8 hpf (hours post fertilization). *edaradd* expression then slowly increased from 12 hpf to 96 hpf [See Additional file 4: Figure S4]. By *in situ* hybridization, we could not detect a regionalized *edaradd* expression pattern (possibly due to low expression levels) [Additional file 5: Figure S5] but both *eda* and *edar* were expressed in the pharyngeal region from 48 and 24 hpf respectively, suggesting that the EDA pathway (and therefore *edaradd*) was active at least in this region during this period of zebrafish development.

We next investigated the function of *edaradd* gene during zebrafish embryonic development using morpholino antisense oligonucleotides directed either against *edaradd* translation initiation site (MO-tsl) to disrupt the translation of the protein or against *edaradd* exon 1 splice donor site to disrupt mRNA splicing (MO-spl) (Fig. 5A). Splice MO efficiency was confirmed by RT-PCR [see Aditional file 4: Figure S4 for details]. The *edaradd* MO-injected larvae exhibited abnormalities of the pharyngeal and cranial skeleton visible at 5.5 dpf (days post fertilization) following cartilage and mineralized tissue staining (Fig. 5B and C), consistent with the expression pattern evidenced for *eda* and *edar*. These embryos were classified depending on the severity of the skeletal defects and on the presence of teeth (see Fig. 5B for a detailed description). MO-tsl and MO-spl-injected larvae led to similar cartilage defects except that MO-spl had weaker effects, even at high concentrations [see Additional file 4: Figure S4].

To study the *in vivo* activities of the two human EDARADD isoforms, we tested their ability to rescue the phenotype of the *edaradd* morphants. We first checked that injection of *EDARADD A* or *B* mRNA or zebrafish *edaradd* in wild-type embryos had no obvious effect even following injection of 100 pg of mRNA per egg [see Additional file 4: Figure S4]. In particular, no supernumerary teeth were detected when observed at 5.5 dpf. We then co-injected 25 to 100 pg human *EDARADD A* or *B* or zebrafish *edaradd* mRNA with MO-tsl in WT embryos (Fig. 5D and E) and analysed the corresponding larvae as above. We first confirmed that zebrafish *edaradd* mRNA efficiently rescued the MO-tsl-induced skeletal defects. Human *EDARADD* B mRNA also provided significant rescue (2.1 ± 1.5 % larvae corresponding to the severe phenotype for MO-tsl + *EDARADD B* mRNA-injected embryos compared to 7.6 ± 3.9 % for MO-tsl-injected embryos (*p* = 0.039)). In sharp contrast, *EDARADD* A mRNA significantly increased the defects observed in MO-tsl-injected embryos (16.3 ± 5.1 % larvae corresponding to the severe phenotype for MO-tsl + *EDARADD* A mRNA-injected embryos compared to 7.6 ± 3.9 % for MO-tsl-injected embryos (*p* = 0.035)). Importantly, both the rescue and the worsening phenotypes were sensitive to the dose of *EDARADD* mRNA injected, with 50 and 100 pg of *EDARADD* mRNA producing significant and equivalent effects whereas 25 pg *EDARADD* mRNA leading only to slight effects (data not shown). Taken together these data show that the two human isoforms are not functionally equivalent in this heterologous system.

## Discussion

In this case study focused on the two alternative isoforms of the *EDARADD* adaptor, we investigated how a signaling pathway important for morphological evolution [22] could evolve functionally by gain and loss of protein isoforms. This let us characterize the evolution but also the function of EDARADD A and B isoforms. As some of our results are of particular interest with respect to the EDA pathway’s role in development and Human Ectodermal Dysplasia (HED) disease [23–26]) we first review and discuss in some detail the interpretation of our biochemical experiments, before returning to on our main focus, which is to integrate the later results with results about isoform evolution.

### EDARADD isoforms: activity and regulation

Because previously little was known about the functional role of EDARADD A isoform, we compared the activity of both human isoforms *in cellulo*, using the same cell lines (HEK cells) as previous studies [19, 20, 23]. HEK cells are kidney epithelial cells. Even if the EDA pathway is mainly known for its role in ectodermal cells, EDA pathway genes are indeed expressed in the kidney (as well as in other epitheliae without an ectodermal origin, e.g. lung, intestine) and *Edaradd* in particular is detected in HEK cells [19]. We focused on the activation of the NF-κB pathway as this is the major pathway activated by EDA [22]. We showed that the A isoform is more active than the B isoform as, for the same quantity of protein, the activation of the NF-κB pathway is 2.5 times higher in a reporter luciferase assay. It is not surprising that both isoforms are capable of activating the NF-κB pathway: beside their N-terminal difference, they share the same functional domains, such as the TRAF binding sites or the death domain interacting with EDAR (Fig. 1). The N-terminal part of proteins is often implicated in protein folding and stability [27], and this might explain two differences observed between the A and B proteins. First, the very N-terminal peptide could influence the folding of the N-terminal part, which may then be specific to each isoform. Since this N-terminal part contains the binding site for TRAF6 (Fig. 1B), a major interactor of EDARADD in transducing downstream signaling [27], a difference in folding may easily turn into the observed difference in level of activation of the NF-κB pathway. Second, the N-terminal peptide could affect the protein stability and explain the difference in steady-state levels. Linked to this, we showed that the A isoform is downregulated by the activation of the pathway, in contrast to the B isoform. This downregulation of the A protein level is linked to the activation of the pathway as the addition of *TRAF 6* further enhances the decrease in protein level. The mechanism for this downregulation is unclear, but does not seem to involve proteasome-dependent degradation. Together, these results obtained in an overexpression system suggested that the human A and B EDARADD proteins are biochemically different, despite that they differ only in a very short N-terminal peptide. However, this difference may impact EDA pathway activation quantitatively rather than qualitatively.

The rescue experiments performed *in vivo* in zebrafish further confirmed this biochemical difference. Before performing this rescue, we characterized the effects on zebrafish development of *edaradd* downregulation by morpholino injection. The latter resulted in defects of pharyngeal tooth formation, which could be expected based on the phenotype observed in teeth of other fishes [21, 28] and mammals [22]. However, the craniofacial skeleton was also abnormal. This was in line with *eda* and *edar* expression in the developing craniofacial region detected from 48 and 24 hpf respectively. Interestingly, both in mouse and human, a mutation in the *Eda* gene leading to defects in the craniofacial skeleton has also been observed [29]. Our results thus highlight an underappreciated role of the EDA pathway in craniofacial development. We then tested the ability of human A and B isoforms to rescue this phenotype. The human B isoform, but not the A isoform, is capable to rescue the *edaradd* MO-induced phenotype. Of note, this rescue observed despite EDARADD B overexpression has no major effect on zebrafish development. This is reminiscent of the results with the K14-Edaradd mice, which overexpress *Edaradd* in skin appendages. These mice display no overt phenotype, yet the transgene can rescue the mutant hair follicle and tail kinking phenotype ([30, 31] and D. Headon, personal communication). This rescue experiment suggests that the B isoform is functionally closer to the single zebrafish Edaradd protein and thus probably closer to the ancestral EDARADD protein predating A isoform gain in mammals. In contrast, the human isoform A is unable to rescue the edaradd MO-induced phenotype. Worse, it tends to enhance phenotype severity. This suggests that the A protein may exert a dominant negative effect on the remaining endogenous Edaradd protein. The human A isoform could compete with the endogenous protein, but being unable to transduce downstream signaling in zebrafish. Alternatively, as suggested *in cellulo*, the human A isoform could be rapidly destabilized following pathway activation and in turn destabilize the endogenous protein through heteromer formation. Whatever the explanation, these experiments showed that the two isoforms are functionally different *in vivo*. These differences will have to be further explored *in vivo* in mammals. For example, in a near future, genome-engineering strategies could in principle be applied to species that possess both isoforms such as guinea pig.

Finally, we also studied the transcriptional regulation of the two isoforms. We found that the A promoter exhibits a weak activity in HEK cells whereas the B promoter is more active and exhibits a feedback regulation following activation of the pathway. Moreover, the B isoform is expressed in all tissues and cell lines checked in this study. In contrast, the A isoform is more often expressed at lower levels in cell lines and is not found in some tissues and cell lines. This suggests that the B promoter may drive B isoform expression in all epithelial tissues making use of the pathway, but be turned off upon activation of the pathway, whereas the A promoter may confer to the A isoform a more tissue-restricted expression, which remains unchanged (at the transcriptional level) when the pathway is activated.

Putting together the transcriptional and post-transcriptional levels, there are clear regulatory differences between the two isoforms despite the fact that both activate the NF-κB pathway. The B isoform is well expressed, stable, activates NF-κB consistently and exhibits feedback regulation at the transcriptional level. The A isoform is expressed in more specific contexts, activates NF-κB strongly but is downregulated by a unknown feedback mechanism at the protein level. Thus it appears that the two isoforms should enable regulating the EDA pathway in a tissue-specific manner and in different ways, in terms of level of activation and dynamics. In the context of the development of ectodermal appendages, [22, 32], such flexibility of the pathway activation is interesting, as the level and dynamics of the pathway activation is expected to have phenotypic consequences on the patterning of ectodermal appendages. Indeed, we know from many examples of experimental tinkering of the pathway that changes in activity translate into phenotypic changes in terms of number, size and shape of ectodermal appendages (e.g. in hair [33] and teeth [34]), some highly resembling natural species variation [35, 36].

### A case study for the evolutionary tinkering of signaling pathways by isoforms evolution

Previously, the expansion of signaling pathways repertoire was mainly explained by gene or genome duplication [1] but recent transcriptomic studies revealing the ubiquity and lability of alternative isoforms [9] suggest to add the evolution of isoforms as a key mechanism. EDARADD nicely exemplifies this notion.

We showed that the B isoform is the ancestral isoform and is conserved in all examined species. Several results suggest that the B isoform has kept the core, vertebrate-conserved, functions of EDARADD: i) it is highly similar at the amino-acid level to the single isoform found in birds and even in fish ii) the human B isoform is able to rescue the zebrafish morphant, iii) it is expressed in all tissues where the pathway is found. In sharp contrast, the A isoform newly evolved in mammals due to a new upstream promoter and was repeatedly lost during mammal diversification. Its expression pattern suggests a more accessory role than isoform B. Despite that it also activates the NF-κB pathway as isoform B, it turned out that it clearly differs from isoform B in terms of transcriptional and post-translational regulation. Therefore, our results suggest that this new A isoform brought new possibilities and levels of regulation to the EDA pathway, different from B isoform. Moreover, our results highlight the highly mosaic albeit nonrandom evolution of A isoform. Indeed, A isoform is present in both marsupial and placental mammals (Fig. 2, see also Fig. 4 for expression) that diverged 165–215 MYA [37], and in most groups of placental mammals. This contrasts with the high rate of losses found during late (post- KT boundary) mammals diversification. This suggests that the loss of isoform A is specifically associated with mammal diversification and the underlying extensive diversification of their ectodermal appendages (hair, teeth, glands, nails…) in terms of number and shape. It is not easy to find a common phenotypic trend in the lists of species where A isoform is lost. Although we may have missed it, we rather favor the possibility that the independent losses of A isoform are associated with a number of different phenotypic changes. Indeed, it is likely that isoform A regulation evolved rapidly along the mammalian tree together with changes in ectodermal appendages, the loss in some species being just extreme cases in which the regulation diverged up to be lost. The loss could occur either because changes in ectodermal appendages phenotype made it dispensable or because the loss participated in reaching an adaptive phenotype.

## Conclusions

In summary, as the EDA pathway is a hot spot of adaptation [22], it is easy to imagine that by modulating the level of activation of the EDA pathway through space and time, the regulation of these two isoforms (and especially A) could follow or sometimes allow rapid evolution of ectodermal appendages. Simultaneously, the two isoforms (and especially B) could ensure that essential functions are maintained. If true, this duality between an ancestral slowly evolving isoform and a newly rapidly evolving isoform may provide a strong evolvability to the pathway in terms of spatio-temporal regulation, while ensuring that essential functions are maintained. This case study suggests we pay greater attention to mosaic evolution of isoforms as an important mechanism of phenotypic change.

## Methods

### Exon 1A region sequence

The exon 1A region of 24 Mammals (21 rodents, three non-rodent) was obtained by extracting total genomic DNA from ethanol-preserved tissues obtained from the collection of Preserved Mammalian Tissues of the Institut des Sciences de l’Evolution of Montpellier (France) with a DNeasy Blood and Tissue extraction kit (Qiagen). The region was then PCR amplified and sequenced twice independently using primers F4 AGAARGAACCACARACCAAACCTC and R4 TTTGGCATTAGTTACTGCCTGACC. Other mammal species sequences were obtained by tBLASTN on Ensembl genomes and TRACE release 59 (3 August 2010) accession number are in the Additionnal file 6: Dataset S6. Sequences were aligned using Muscle (http://www.drive5.com/muscle/) [38] followed by manual refinements and visualized in Seaview (http://pbil.univ-lyon1.fr/software/seaview.html). [See dataset in Additional file 6: Dataset S6].

### *in vitro* translation experiments

Eight exon 1A constructs (Human, Human ATG1 mutant, Human ATG2 mutant, Human double mutant, *Cavia porcellus*, *Equus caballus*, *Meriones crassus*, *Vicugnia pacos*) were synthetized (the limits of exon 1A in the different species being defined by sequence homology to the human exon 1A) , cloned in *p0Renilla* vector and tested by *in vitro* translation as [39]. Briefly, *p0Renilla*-derived vectors containing a 100 nt long poly(A) tail were linearized by EcoRI digestion. Capped mRNAs were transcribed *in vitro* 2 h at 37 °C using 1 μg of linear DNA template; 80 U of T7 RNA polymerase (Promega); 40U of RNAsin (Promega); 10 mM of rCTP, rUTP, rATP; 0.36 mM rGTP; 30 mM DTT; 1.24 mM of m^7^GpppG cap analogue (New England Biolabs) in transcription buffer (40 mM Tris–HCl (pH 7.9), 6 mM MgCl_2_, 2 mM spermidine and 10 mM NaCl). mRNAs were DNase treated and precipitated with 1 volume of ammonium acetate 5 M (2.5 M final concentration). The integrity of mRNAs was checked on agarose gel electrophoresis and their concentration was quantified by spectrophotometry at 260 nm using Nanodrop (NanoDrop Technologies, Wilmington, DE, USA). Then, *in vitro* transcribed RNAs were translated for 30 min at 30 °C in 10 μl of the supplemented untreated RRL or the Flexi® Rabbit Reticulocyte System 50 % (v/v) each (Promega Co., Madison, WI, USA) in the presence of KCl (75 mM), MgCl_2_ (0.5 mM), 20 μM of amino acids mix minus methionine and 0.6 μCi of [^35^S]-methionine (GE Healthcare Life Sciences). Reactions were stopped with 2 × SDS-loading buffer and the products were resolved by 15 % SDS–PAGE. Gels were dried and subjected to autoradiography using Biomax films (Eastman Kodak Co.).

### Promoter analysis

We amplified by PCR the 5′ region of exon 1A in human and exon 1B in human and mouse. We took 2900 pb upstream the ATG of the exon 1A for the human A promoter and the entire region between the end of the exon 1A and the ATG of the exon 1B for the human B and mouse B promoters. We used: for human A promoter: FhumA 5′- TGGCACATTCTGAGGCATAG-3′, RhumA 5′- AAGATGCTCGCTGGTTGTCT-3′; for human B promoter: FhumB 5′-ATATGCTAGCGCATTTGCTGTCTTCCTGG-3′, RhumB 5′-GAGACTCGAGGGCAAACGAGCGTCCG-3′, for mouse B promoter: FmusB 5′- ATATGAGCTCAACTCCAGACTCCCCTCCT-3′, RmusB 5′-ATATCTCGAGCCCTGAGAACAGCAGGAT-3′.

These fragments were cloned into pGL2 basic vector upstream luciferase reporter gene to test the ability of the two putative promoters to initiate the transcription in HEK cells. HEK cells grown in 24-well plates were then transfected using Exgen500 transfection reagent (Euromedex). Each well received 400 ng of each promoter cloned in pGL2basic vector and 100 ng of β-galactosidase expression plasmid. For the tests with putative activators such as EDAR, TRAF6 or CYLD, promoter vectors were cotransfected with i) growing amount of *EDAR* 50, 100 or 200 ng supplemented by empty pCDNA3, or 200 ng of *EDAR* cloned into pCDNA3, or 200 ng of *TRAF6* cloned into pCDNA3 or 200 ng of *EDAR* and 200 ng of *CYLD* into pCDNA3 kindly gifted by Gilles Courtois and supplemented by empty pCDNA3. Cells were lyzed 24 h after transfection and firefly luciferase activities measured using the Dual-Luciferase Reporter Assay System (Promega). Normalization for transfection efficiency in luciferase reporter assays was done by including a constant amount of a β-galactosidase expression plasmid in all transfections and measuring β-galactosidase activity. Each transfection was performed in triplicate, and three independent experiments were performed.

### RT-PCR experiments

A guinea pig was obtained from Harlan. Treatment satisfied the requirements of the latest european directive 2010/63. Euthanasia was performed by a trained and authorized person (n°69387512), administrating lethal dose of pentobarbital. Guinea pig total RNA was extracted from fresh or RNAlater (sigma) preserved tissues using Qiagen RNA easy kit. *Macropus eugenii* RNAs were a kind gift of Dr Kevin Nicholas (Melbourne University). Human RNA were obtained from Ambion: FirstChoice® Human Total RNA Survey Panel for brain, mammary gland, kidney and skin. All RNAs were reverse transcribed using Superscript III Invitrogen. PCR fragments were amplified using an exon 1A specific primer or an exon 1B specific primer and a reverse primer in exon 5, respectively: F-A TGGTCCCACGCAGCACTAGC, F-B 5′-GAGACCCGGGACGAACAGGC-3′, R 5′-AGGGAGCGCTGTATCTTGAATGGG-3′ for *Cavia porcellus*; F-A 5′-ATGGGACTCAGGACTACAAAGCA-3′ F-B 5′-ATGGGACTCAGGACTACAAAGC-3′ R 5′-AGGGTGCGCTGTATCTTGAATGGG-3′ for *Macropus eugenii*, F-A 5′-ATGGGCCTCAGGACGACTAAACAGATGG-3′, F-B 5′-TGCGCGCAGATCATATGGTAAAGGAAC-3′ and R 5′-ACCTGCGCAGAACCTTCTCCACGTC-3′ for *Homo sapiens*. Sequences were deposited in Genebank with the following accession numbers: XXXX.

### RNAseq analysis

The following datasets were downloaded from ENCODE website (http://genome.ucsc.edu/cgi-bin/hgTrackUi?hgsid=346780313&c=chr1&g=wgEncodeCaltechRnaSeq, human genome version hg19, GRCh37: GM92.paired.75 nt.; NHEK.paired.75 nt.200pb; GM78.paired.75 nt.200pb; H1ES.paired.75 nt.200pb; K562.paired.75 nt.200pb; MCF7.paired.75 nt.200pb; GM91.paired.75 nt.200pb. These cells lines were selected because of their relevance regarding the role of the Eda pathway in mammals: GM92, GM78 and GM91 cells are derived from mammary gland cell line, HE1S cells are derived from embryonic cell lines, NHEK are derived from epidermal keratinocytes, K562 cells are derived from leukemia and MCF7 cells are derived from mammary gland carcinoma.

To estimate the number of reads for each exon, we used the reads mapped on the human genome version hg 19 on ENCODE website. For each cell line and replicate, we extracted the BAM file corresponding table and discarded ambiguous reads. We then obtained specific reads for each exon 1A and 1B and for constitutive exon 6 as a control. As the number of read depends on exon size, we normalized the number of reads by exon size (228 bp for exon 1A, 245 bp for exon 1B and 426 bp for exon 6) and checked that the number of reads was coherent by ensuring that exon 1A + exon 1B = exon 6. True number of reads mapping to exon A and exon B were compared to expected numbers under the hypothesis that both isoforms were expressed at the same levels. This was done in each library, by using Chi-squared test comparing observed and expected distributions for the number of reads and corrected for multiple tests (7 tests). Results were unambiguous, as the 4 lines showing a significant difference in expression levels between A and B (NHEK (*p* = 10-15), H1ES (10–27), K562 (10–60), MCF7 (10–17)), also showed a clearly skewed fold change (more than 8–10 times in exon B compared to exon A).

### NF-κB reporter assays and Western Blotting

Expression vectors for EDARADD B were kindly gifted by Asma Smahi and Céline Cluzeau and included 1) Full coding sequence of human *EDARADD B* tagged with a C-terminal myc tag and cloned in pCDNA3.1 and 2) Full coding sequence of human *EDARADD B* tagged with a N-terminal HA tag and cloned in pCDNA 3. We amplified the *EDARADD A* N-terminal end by RT-PCR from human RNA and we used recombination to replace the sequence encoding the B peptide by the sequence encoding the A peptide directly in the above mentioned *EDARADD B* vectors (InFusion Cloning system, Clonetch).

For NF-κB activation assays, HEK cells grown in 24-well plates were transfected using Exgen500 transfection reagent (Euromedex). Each well received 200 ng of IgKLuc luciferase reporter (Clontech) kindly gifted by Asma Smahi and Céline Cluzeau as well as the above *EDARADD* pcDNA3 vectors and empty pCDNA3 vector to give 1.0 μg of DNA per well. Normalization for transfection efficiency in luciferase reporter assays was done by including a constant amount of a β-galactosidase expression plasmid in all transfections and measuring β-galactosidase activity. Cells were lyzed 24 h after transfection and luciferase activities measured using the Dual-Luciferase Reporter Assay System (Promega), according to the manufacturer’s protocol. Each transfection was performed in triplicate, and three independent experiments were performed.

For Western Blotting, HEK cells grown in 6-well plates were transfected with 1.5 μg of pCDNA3.1-*EDARADD-myc* or empty pCDNA3 or 1.5 μg pCDNA3.1-*EDARADD*-myc cotransfected with 750 ng of *EDAR* or *TRAF6* supplemented by empty pCDNA3 using Exgen500 transfection reagent (Euromedex). 24 hours or 48 hours after transfection, cells were harvested using ice-cold PBS and lysed in 50 μL of RIPA buffer. Proteins were fractionated in 12 % SDS/polyacrylamide gel electrophoresis and transferred to a nitrocellulose membrane. Mouse anti-HA antibody (1:5000), mouse anti-myc antibody (1:5000) and rabbit anti-histone H3 (ab1791, Abcam) were used as primary antibodies and the reactive bands were detected via an ECL kit (Amersham Life Sciences).

### Fish husbandry

Zebrafish and their embryos were handled according to standard protocols [40] and in accordance with the animal welfare committees of the Ecole Normale Supérieure de Lyon. Zebrafish strains of AB/Tü were reared and staged at 28.5 °C according to Kimmel and collaborators.

### RT-PCR for zebrafish experiments

Total RNA was extracted from 25 embryos at various time points using the Nucleospin RNA II kit (Macherey-Nagel) according to the protocol for animal tissues and 1 μg of RNA was reverse-transcribed using Moloney murine leukemia virus reverse transcriptase (Promega), followed by amplification of desired cDNA by PCR with Taq DNA polymerase with ThermoPol buffer (BioLabs). The ENSEMBL accession number for Danio rerio *edaradd* mRNA was ENSDART00000089916. Standard PCR primers for *edaradd* mRNA detection and splice blocking morpholino validation were as follows: For-*edaradd*, 5′-GCTCTGTGTTCCCACATCAA-3′; Rev-*edaradd*, 5′-ACCAGGAGGAACAGAGCTGA-3′. The *actin1* gene was used as a control: For-*β*-*actin1*, 5′-AAGCAGGAGTACGATGAGTCTG-3′; and Rev-*β*-*actin1*, 5′-GGTAAACGCTCCTGGAATGAC-3′.

### Morpholino knockdown

Morpholino oligonucleotides (*edaradd* MO-tsl (translation blocking): 5′-CACACATAGAAGAAGATCGTTGATC-3′; *edaradd* MO-spl (splice blocking): 5′-CACCAAGCGTATTTACTTACCAAAC-3′) and the corresponding mismatch controls (MO sequence containing five base mismatches): *edaradd* MS-tsl: 5′-CAgAgATAcAAGAAcATCcTTGATC-3′ and *edaradd* MS-spl: 5′-CACgAAcCGTATaTAgTTAgCAAAC-3′) were supplied by Gene Tools and diluted in sterile water containing 0.05 % phenol red. One-cell stage AB/Tü embryos were injected with 0.5 nl of morpholino oligonucleotide dilution (1 mM for MO-tsl and 2 mM for MO-spl) and then incubated at 28.5 °C in E3 medium (5 mM NaCl, 0.17 mM KCl, 0.33 mM CaCl2, and 0.33 mM MgSO4). The phenotypic observations of morphants were performed with a stereomicroscope or a light microscope (all from Leica) equipped with a digital camera. MO-injected embryos were routinely compared with corresponding MS-injected, phenol red-injected, and uninjected embryos.

### RNA preparation and rescue experiments

Capped *Homo sapiens EDARADD*-*A* and -*B* and *Danio rerio edaradd* were synthesized and purified from pCS2+:*EDARADD*-*A* and *B* and pCS2+:*edaradd* plasmids by using the mMESSAGE mMACHINE Sp6 kit (Ambion). For the rescue experiments, 25, 50 and 100 pg of capped human *EDARADD*-*A*, −*B* mRNA or capped zebrafish *edaradd* mRNA were co-injected with *edaradd* MO-tsl at the one-cell stage. These one-cell stage embryos were co-injected with MO-tsl and/or capped human *EDARADD* A or B mRNA with at least 70 embryos injected per condition. The barplot shows the percentage of each phenotype condition and is representative of at least 3 separate experiments with 3 different capped mRNA productions. Results are given as the mean ± S.E. The significance of differences between mean values was evaluated by Student t test (*, *p* < 0.05; **, *p* < 0.01).

### Zebrafish staining and whole mount *in situ* hybridization

Alcian blue (cartilage) and Alizarin red (bone) double staining was performed as described [41] with minor modifications: staining was performed overnight on 5.5 dpf larvae with a solution containing 0.02 % (w/v) Alcian Blue 8GX, 100 mM MgCl_2_ and 0.005 % (w/v) Alizarin red in 70 % EtOH. Larvae were then bleached 20 min in 1.5 % (v/v) H_2_O_2_ and 1 % (w/v) KOH and then digested for 5 min in 1.67 % (w/v) trypsin.

Whole mount *in situ* hybridization was performed as previously described [42]. Staging was performing according to Kimmel. Sense and antisense RNA probes for each gene tested were prepared from partial cDNA. The development of endogenous pigments was inhibited by exposing embryos to 1-phenyl-2-thiourea (PTU) at a final concentration of 0.2 mM.

## Additional files

Additional file 1: Figure S1. Genomic region of human EDARADD gene. Genomic region of the *EDARADD* gene in human (version hg19) vizualized in the UCSC genome browser (https://genome-euro.ucsc.edu/cgi-bin/hgTracks?db=hg19&position=chr1%3A236480456-236681365&hgsid=198526640_PuRKSKevW48nfe6o7ZmzCdqA0Kun). Genomic coordinates are the following: Chromosome 1: 236,348,262-236,484,914, exon 1A: Chr 1: 236,394,278-236,394,505, exon 1B: Chr 1: 236,395,416-236,395,660. In human ENCODE 7 cell, exon 1 A and B transcription initiation sites are embedded into a single H3K27ac peak, an epigenetic mark which is associated with initiation sites and active enhancers (green arrow). Human EST can be visualized above the gene organization, with 1A or 1B alternative first exon. Upstream, two human spliced EST match another H3K27ac peak (red arrow). They are composed of three exons matching this region and exons 2–6 of *EDARADD*. Such a transcript could possibly encode an *EDARADD* protein (that would then be initiated with an internal methionine as compared with A and B proteins, although it is not a consensus kozak sequence: gatcatATGG/gccA/GccATGG). The functional significance of this putative *EDARADD* transcript is however very dubious. Not only ESTs matching these 3 additional exons are strongly underrepresented in EST databases as compared with EST matching exons 1A et 1B, but they all come from testis, a tissue which is known for showing leaky transcription. We thus tend to believe that transcriptional activity at this site may rather reflect the presence of a distant enhancer of Edaradd (enhancers are known to be transcribed and show H3K27ac epigenetic mark as initiation sites) rather than the transcription of protein coding transcript. We nevertheless investigated the presence of a homologous transcript in other species. No upstream initiation site is found in mouse (EST database screening), suggesting that if this upstream H3K27ac peak corresponds to a third alternative promoter, this promoter is not conserved in mouse. Parts of the genomic region spanning the H3K27ac peak are conserved in the cow, but found on another chromosome than *EDARADD* (chr8 instead of 28); and no homologous region is found in dog; suggesting that if there is a third *EDARADD* alternative promoter, which we find unlikely, it would not be conserved in mammals. In conclusion, we did not consider this putative human third *EDARADD* transcript/promoter in the rest of the manuscript because its functional significance is dubious and moreover it does not seem conserved in mammals. Additional file 2: Figure S2. Exon 1 outside mammals. Coding sequence of exon 1B is well conserved in mammals and even in vertebrates whereas the one of exon 1A is variable with a frequent loss of ATG (regarding A isoform, for an augmented dataset, see Fig. 1 and [Additional file 6 - Dataset S6]). Additional file 3:Figure S3. EDARADD A and B do not exhibit any synergic or antagonistic effect for NF-κB activation. *EDARADD* A and B were transfected and cotransfected in HEK cells as followed: *EDARADD* A 300 ng, *EDARADD* B 300 ng, *EDARADD* A 200 ng with *EDARADD* B 100 ng, *EDARADD* A 100 ng with *EDARADD* B 200 ng, *EDARADD* A 150 ng with *EDARADD* B 150 ng. Each time, a NF-κB reporter plasmid that harbors NF-κB response elements upstream a luciferase reporter gene was cotransfected. The luciferase activity was normalized against the vector. The luciferase activity was assayed 24 hours after transfection. Additional file 4: Figure S4. Details for experimental validation of *edaradd* knockdown with morpholinos and *edaradd* rescue experiments in zebrafish. A: *edaradd* mRNA level in zebrafish during embryonic development. *edaradd* mRNA level was analyzed by RT-PCR on whole embryos from 2.5 to 120 hpf. *edaradd* is highly detected as a maternal transcript before zygotic transcription starts. At 12 hpf, zygotic *edaradd* mRNA transcription starts and increases until 96 hpf. This result is representative of 3 independent experiments. B: Validation of Morpholino-induced *edaradd* downregulation. WT embryos were injected at one-cell stage with 4 ng translation- (MO-tsl) or 8 ng splice-blocking *edaradd* MO (MO-spl) and *edaradd* mRNA expression was analyzed by RT-PCR using specific primers to amplify DNA sequence either side of the targeted splice donor site (between exons 1 and 4) (see Fig. 5A for primer and morpholino localization). At 48 hpf, we observed an absence of PCR product in MO-spl-injected embryos when compared to MS-spl-, MO-tsl- and MS-tsl-injected embryos demonstrating that the MO-spl efficiently blocked *edaradd* splicing. As expected, neither injection with translation blocking MO (MO-tsl) nor with its mutated version (MS-tsl) impacted *edaradd* mRNA levels. This result is representative of 3 independent experiments. C: Knockdown of *edaradd* expression with MO-tsl or MO-spl. Percentages for the various phenotypic groups obtained following MO-tsl injection were determined and are representative of 3 independent experiments; each experiment was performed with at least 70 injected embryos. D: Determination of human *EDARADD* A and B role through rescuing experiments in zebrafish. One-cell stage embryos were co-injected with MO-tsl and/or capped human *EDARADD* A or B or zebrafish *edaradd* mRNA. At least 70 embryos were injected per condition and the results are expressed as the percentage of each phenotypic groups observed per condition. Results are representative of 6 separate experiments performed with 2 different batches of mRNA. Statistical analyses have been performed on the severe phenotypic condition. E: Overexpression of human EDARADD A or B isoforms or zebrafish Edaradd has no obvious effect on skeletal development. WT embryos were injected with 100 pg of capped human *EDARADD A* or *B* mRNA or zebrafish *edaradd* mRNA at one-cell stage and skeletal morphogenesis was analyzed through alcian blue and alizarin red staining at 5.5 dpf. A representative ventral view of the head of a larvae is shown for each condition and a zoom on the teeth is shown on the right panel (scale bar: 100 μm). Additional file 5: Figure S5. Expression of Eda and Edar in the pharyngeal region during zebrafish embryogenesis determined using whole mount *in situ* hybridization. A: Coexpression of *eda* and *edar* at 60hpf in the pharyngeal region in lateral view (up) and ventral view (low). B: Expression pattern of *eda* from 48 to 80 hpf. For each stage a lateral view is shown with, as an inset, a ventral view. No expression was detected before 48hpf. C: Expression pattern of *edar* from 24 to 80 hpf. For each stage a lateral view is shown with, as an inset, a ventral view. No expression was detected before 24 hpf. Additional file 6: Dataset S6. Sequences dataset of the exon 1A region. Alignment of the exon 1A region obtained by BLAST or sequencing (see material and method for details) in Seaview software. Human TSS (Transcription Start Site) and the two ATG are annotated.
