# Supplementary figures and images for: Tinkering signaling pathways by gain and loss of protein isoforms: the case of the EDA pathway regulator EDARADD
Source: BMC Evol Biol. 2015 Jul 2;15:129. doi: 10.1186/s12862-015-0395-0 (PMC4489351; doi:10.1186/s12862-015-0395-0)

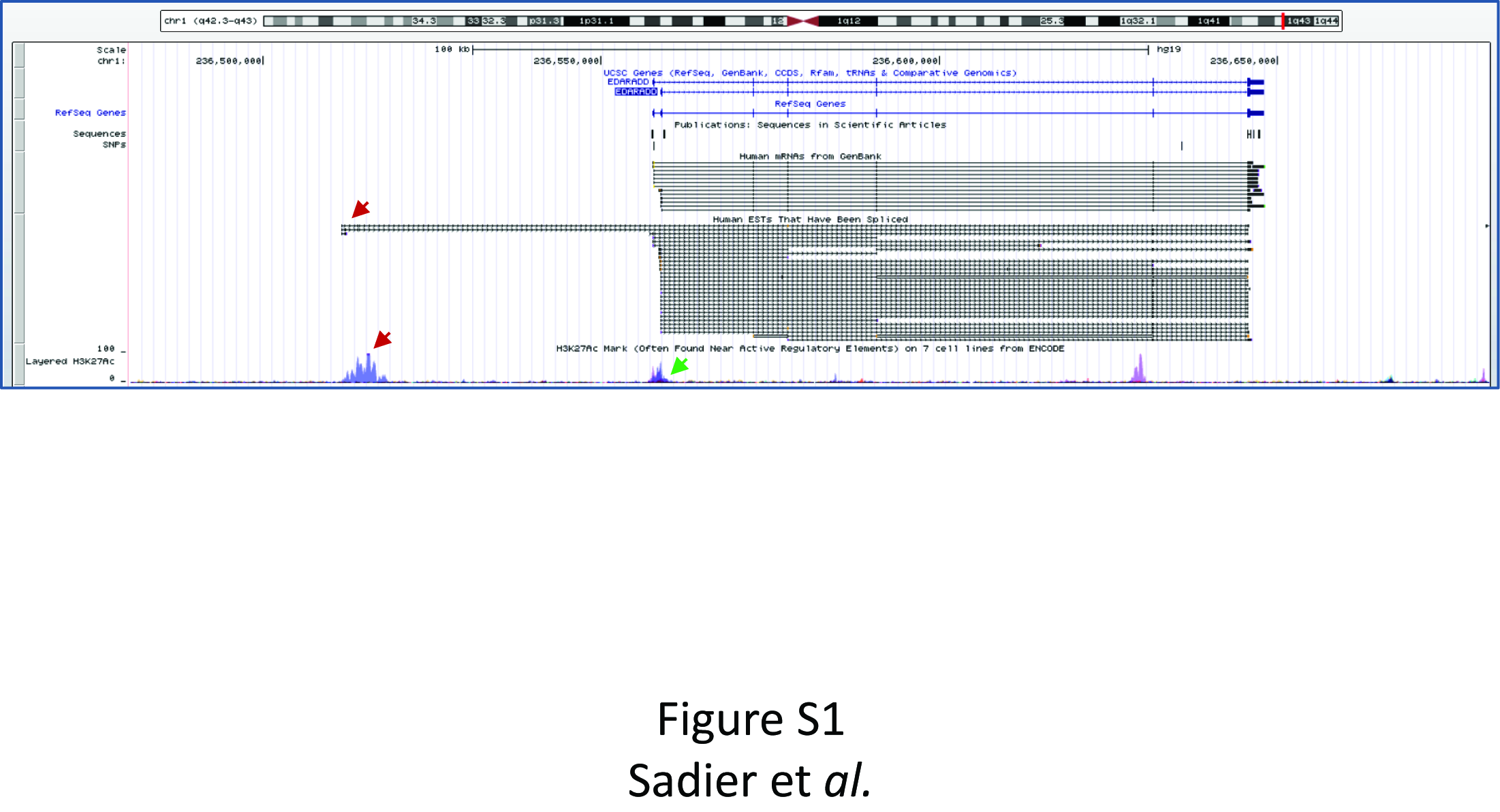

Supplement: Additional file 1: Figure S1. — Genomic region of human EDARADD gene. Genomic region of the EDARADD gene in human (version hg19) vizualized in the UCSC genome browser (https://genome-euro.ucsc.edu/cgi-bin/hgTracks?db=hg19&position=chr1%3A236480456-236681365&hgsid=198526640_PuRKSKevW48nfe6o7ZmzCdqA0Kun). Genomic coordinates are the following: Chromosome 1: 236,348,262-236,484,914, exon 1A: Chr 1: 236,394,278-236,394,505, exon 1B: Chr 1: 236,395,416-236,395,660. In human ENCODE 7 cell, exon 1 A and B transcription initiation sites are embedded into a single H3K27ac peak, an epigenetic mark which is associated with initiation sites and active enhancers (green arrow). Human EST can be visualized above the gene organization, with 1A or 1B alternative first exon. Upstream, two human spliced EST match another H3K27ac peak (red arrow). They are composed of three exons matching this region and exons 2–6 of EDARADD. Such a transcript could possibly encode an EDARADD protein (that would then be initiated with an internal methionine as compared with A and B proteins, although it is not a consensus kozak sequence: gatcatATGG/gccA/GccATGG). The functional significance of this putative EDARADD transcript is however very dubious. Not only ESTs matching these 3 additional exons are strongly underrepresented in EST databases as compared with EST matching exons 1A et 1B, but they all come from testis, a tissue which is known for showing leaky transcription. We thus tend to believe that transcriptional activity at this site may rather reflect the presence of a distant enhancer of Edaradd (enhancers are known to be transcribed and show H3K27ac epigenetic mark as initiation sites) rather than the transcription of protein coding transcript. We nevertheless investigated the presence of a homologous transcript in other species. No upstream initiation site is found in mouse (EST database screening), suggesting that if this upstream H3K27ac peak corresponds to a third alternative promoter, this promoter is not conserved in [file 12862_2015_395_MOESM1_ESM.tiff]

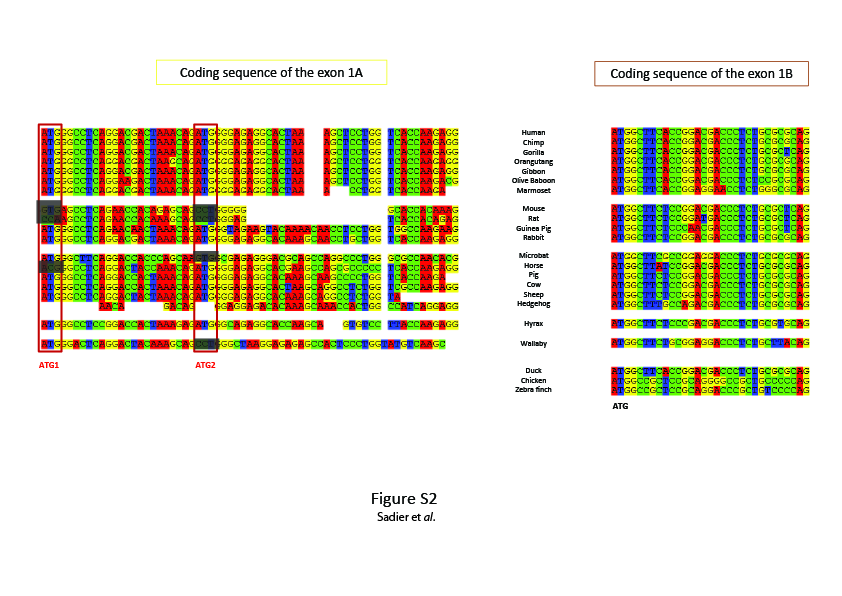

Supplement: Additional file 2: Figure S2. — Exon 1 outside mammals. Coding sequence of exon 1B is well conserved in mammals and even in vertebrates whereas the one of exon 1A is variable with a frequent loss of ATG (regarding A isoform, for an augmented dataset, see Fig. 1 and [Additional file 6 - Dataset S6]). [file 12862_2015_395_MOESM2_ESM.tiff]

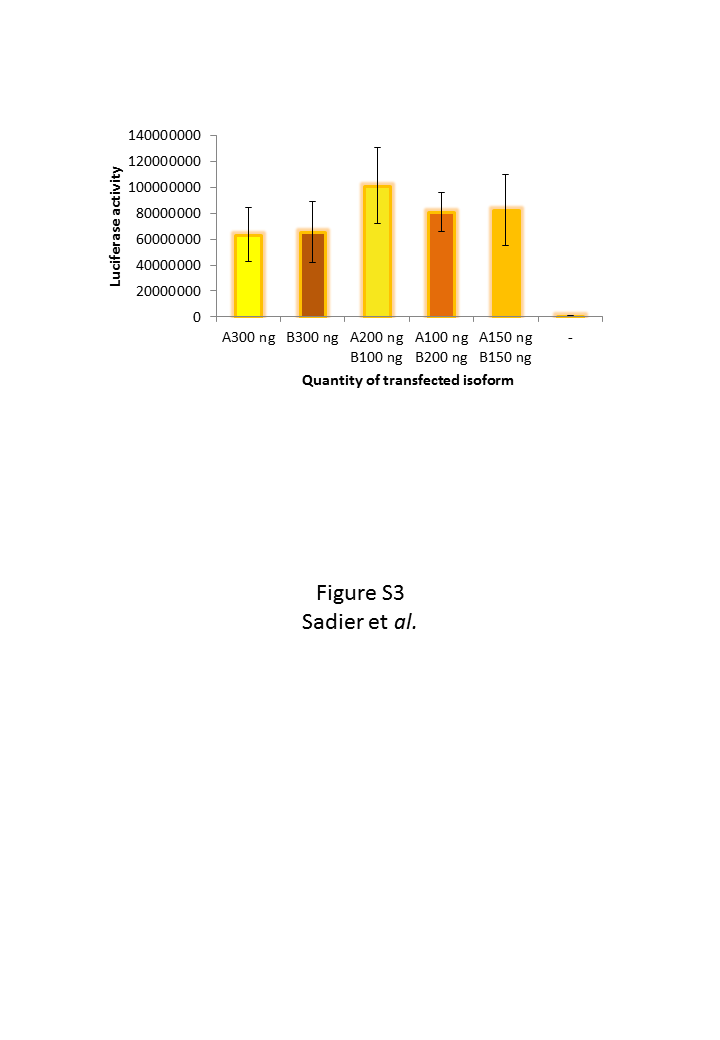

Supplement: Additional file 3:Figure S3. — EDARADD A and B do not exhibit any synergic or antagonistic effect for NF-κB activation. EDARADD A and B were transfected and cotransfected in HEK cells as followed: EDARADD A 300 ng, EDARADD B 300 ng, EDARADD A 200 ng with EDARADD B 100 ng, EDARADD A 100 ng with EDARADD B 200 ng, EDARADD A 150 ng with EDARADD B 150 ng. Each time, a NF-κB reporter plasmid that harbors NF-κB response elements upstream a luciferase reporter gene was cotransfected. The luciferase activity was normalized against the vector. The luciferase activity was assayed 24 hours after transfection. [file 12862_2015_395_MOESM3_ESM.png]

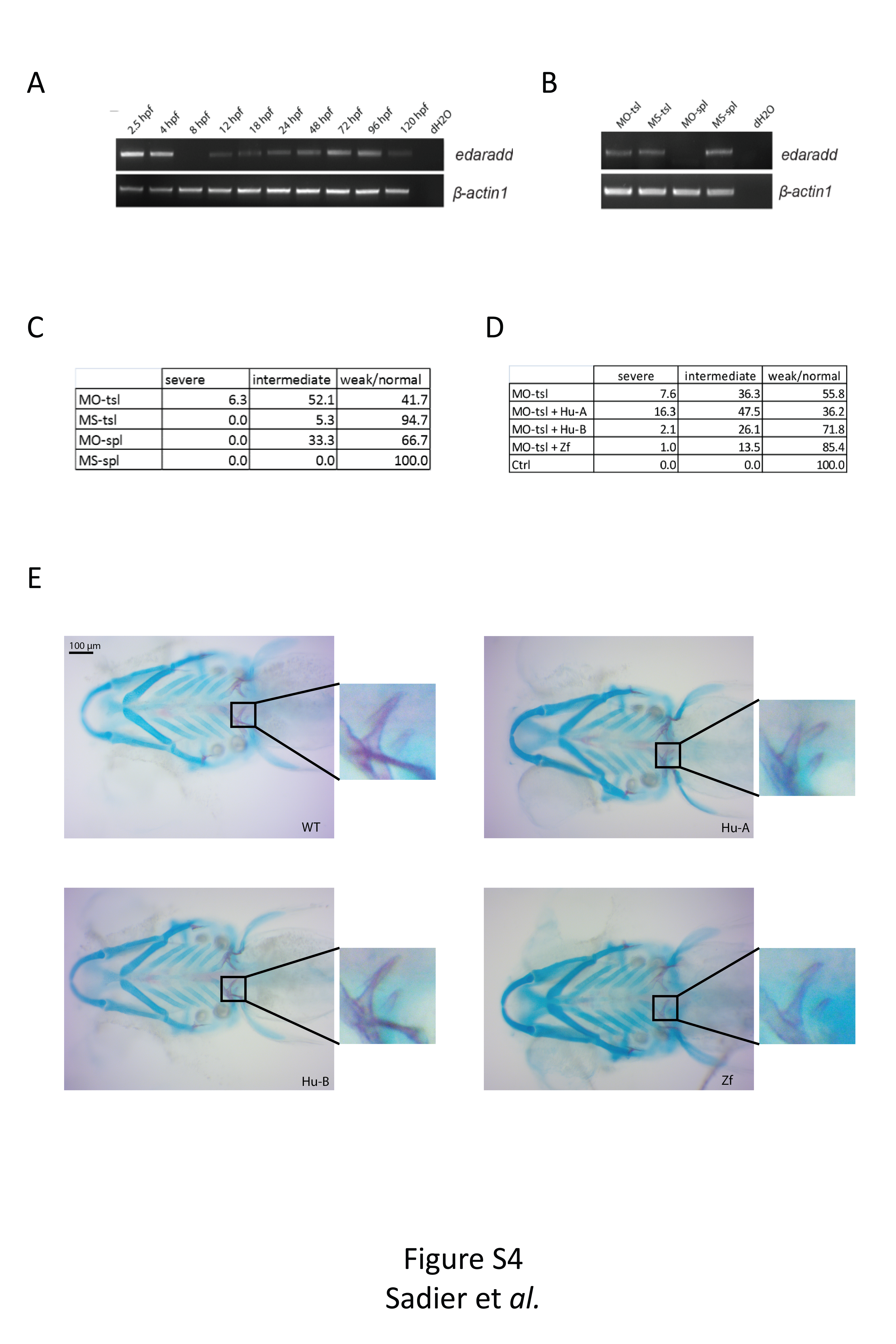

Supplement: Additional file 4: Figure S4. — Details for experimental validation of edaradd knockdown with morpholinos and edaradd rescue experiments in zebrafish. A: edaradd mRNA level in zebrafish during embryonic development. edaradd mRNA level was analyzed by RT-PCR on whole embryos from 2.5 to 120 hpf. edaradd is highly detected as a maternal transcript before zygotic transcription starts. At 12 hpf, zygotic edaradd mRNA transcription starts and increases until 96 hpf. This result is representative of 3 independent experiments. B: Validation of Morpholino-induced edaradd downregulation. WT embryos were injected at one-cell stage with 4 ng translation- (MO-tsl) or 8 ng splice-blocking edaradd MO (MO-spl) and edaradd mRNA expression was analyzed by RT-PCR using specific primers to amplify DNA sequence either side of the targeted splice donor site (between exons 1 and 4) (see Fig. 5A for primer and morpholino localization). At 48 hpf, we observed an absence of PCR product in MO-spl-injected embryos when compared to MS-spl-, MO-tsl- and MS-tsl-injected embryos demonstrating that the MO-spl efficiently blocked edaradd splicing. As expected, neither injection with translation blocking MO (MO-tsl) nor with its mutated version (MS-tsl) impacted edaradd mRNA levels. This result is representative of 3 independent experiments. C: Knockdown of edaradd expression with MO-tsl or MO-spl. Percentages for the various phenotypic groups obtained following MO-tsl injection were determined and are representative of 3 independent experiments; each experiment was performed with at least 70 injected embryos. D: Determination of human EDARADD A and B role through rescuing experiments in zebrafish. One-cell stage embryos were co-injected with MO-tsl and/or capped human EDARADD A or B or zebrafish edaradd mRNA. At least 70 embryos were injected per condition and the results are expressed as the percentage of each phenotypic groups observed per condition. Results are representative of 6 separate experiments performed with 2 differen [file 12862_2015_395_MOESM4_ESM.png]

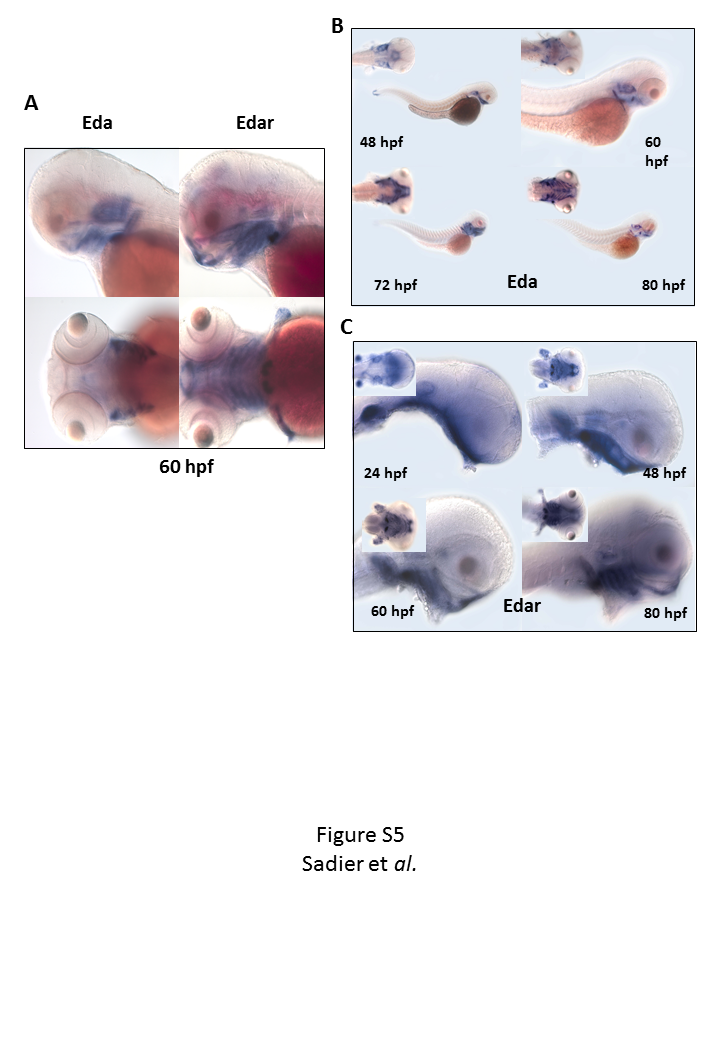

Supplement: Additional file 5: Figure S5. — Expression of Eda and Edar in the pharyngeal region during zebrafish embryogenesis determined using whole mount in situ hybridization. A: Coexpression of eda and edar at 60hpf in the pharyngeal region in lateral view (up) and ventral view (low). B: Expression pattern of eda from 48 to 80 hpf. For each stage a lateral view is shown with, as an inset, a ventral view. No expression was detected before 48hpf. C: Expression pattern of edar from 24 to 80 hpf. For each stage a lateral view is shown with, as an inset, a ventral view. No expression was detected before 24 hpf. [file 12862_2015_395_MOESM5_ESM.png]
