# Supplementary material for: Tinkering signaling pathways by gain and loss of protein isoforms: the case of the EDA pathway regulator EDARADD
Source: BMC Evol Biol. 2015 Jul 2;15:129. doi: 10.1186/s12862-015-0395-0 (PMC4489351; doi:10.1186/s12862-015-0395-0)
Supplement: Additional file 6: Dataset S6. — Sequences dataset of the exon 1A region. Alignment of the exon 1A region obtained by BLAST or sequencing (see material and method for details) in Seaview software. Human TSS (Transcription Start Site) and the two ATG are annotated. [file 12862_2015_395_MOESM6_ESM.pdf]

Alignment: C:\Users\asadier\Thèse\These\Papiers\PNAS edaradd\Edaradd fichier supplementary
Seaview [blocks=10 fontsize=10 A4] on Tue Jan 14 16:35:20 2014

```
                           1
Monodelphis                TCTC-AGAAA -TTAGCCCC- --TTTATGAG GAAAGCA--- -GAACCACAA AACA--AACT
Macropus                   TCTC-AAAAA -TTAGCCCG- --TTTATGAG AAAAGCA--- -GAACCACAA CACA--AACT
Microcebus_murinus         CGTC-AGAGA -TCATTCCAC C-TGCACCAG AAGTACAGAA AGAACCACAA ACCA--AACC
Tarsius_syrichta           CGTC-AGAAC -TAATTCCAT C-TTCCTGAC ATGTCCAGAA AGAACCACAA ACCA--AACC
Callithrix_jacchus         CGTC-AGAA- -TAATTCCAT C-TTCATGAG AATTACAGAA AGAACCACAA ACCA--AACC
Macaca_mulatta             CGTC-AGAA- -TAATTCCAT C-TTCATGAG AAGTACAGAA AGAACCACAA AC-A--AACC
Pongo_pygmaeus             CGTC-AGAA- -TAATTCCAT C-TTCATGAG AAGTACAGAA GGAACCACAA ACAA--AACC
Gorilla_gorilla            CGTC-AGAA- -TAATTCCAT C-TCCATGAG AAGTACAGAA AGAACCACAA ACCA--AACC
Homo_sapiens               CGTC-AGAA- -TAATTCCAT C-TCCATGAG AAGTACAGAA AGAACCACAA ACCA--AACC
Pan_troglodytes            CGTC-AGAA- -TAATTCCAT C-TCCCTGAG AAGTACAGAA AGAACCACAA ACCA--AACC
Ochotona_princeps          CCTC-ACAAA -TTATTCCAT C-TTCATGTG AAGTACAGA- GGGACCACAA ACCA--AACC
Oryctolagus_cuniculus      CCTC-ACAAA -CAATTCCAT C-TGCATGTA AAGTACAGAA AGAACCACAA ACCA--AACC
Marmota_monax              ---------- ---------- ---------- ---------- ---------- ----------
Aplodantia_rufa            ---------- ---------- ---------- ---------- ---------- ----------
Spermophilus               CCAC-AAAAA -TAATTCCGA C-TTCATGTG AAGTACAGAA AGAACCACAA ACCA--AACC
Anomamorus                 ---------- ---------- ---------- ---------- ---------- ----------
Maxomys                    ---------- ---------- ---------- ---------- ---------- ----------
Rattus rattus              ---------- ---------- ---------- ---------- ---------- ----------
Rattus exulans             ---------- ---------- ---------- ---------- ---------- ----------
Rattus_norvegicus          CCTC-AGAAT TGAATTCCAT C-TCCATGTG AAGCATATAA GGAACCACAG GCCA--AACC
Mus_musculus               CCTC-AGAAA TGAATTCCAT C-TCTGCGTG GAGTATAGAA GGAACCACAG ACCA--AACC
Mus_spretus                ---------- ---------- ---------- ---------- ---------- ----------
Mus_pahari                 ---------- ---------- ---------- ---------- ---------- ----------
Praomys                    ---------- ---------- ---------- ---------- ---------- ----------
Meriones                   ---------- ---------- ---------- ---------- ---------- ----------
Meriones_crassus           ---------- ---------- ---------- ---------- ---------- ----------
Acomys cahirinus           ---------- ---------- ---------- ---------- ---------- ----------
Lophuromys sikapusi        ---------- ---------- ---------- ---------- ---------- ----------
Mesocricetus_auratus       ---------- ---------- ---------- ---------- ---------- ----------
Oryzomys                   ---------- ---------- ---------- ---------- ---------- ----------
Trichys_fasciculata        ---------- ---------- ---------- ---------- ---------- ----------
Heterocephalus_glaber      ---------- ---------- ---------- ---------- ---------- ----------
Coendou_melanurus          ---------- ---------- ---------- ---------- ---------- ----------
Cavia_porcellus            CATC-AGAAA -TAATTCCAT C-AGCATGTG AAGTACAGAT AGGACCACAA ACCA--AACC
Ctenomys_maulinus          ---------- ---------- ---------- ---------- ---------- ----------
Octodon_degus              ---------- ---------- ---------- ---------- ---------- ----------
Loxodonta_africana         TTTC-AGAAA -TGATTCCAT T-TTCATGTA AAGTACAGAA AGAACCACAA AAAA-CAACC
Procavia_capensis          GCTC-AGAAA -TTATCCCAT C-TGCATGA- AAGTACAGAA AGAACCACAA AAAAATAACC
Echinops_telfairi          CCTC-AGAAA -TTATTCCAT C-TTCATGAG AAATACAGAA CAAACCACAA AACA--AACC
Micropotamogale            ---------- ---------- ---------- ---------- ---------- ----------
Myotis_lucifugus           CCTC-AGTAA -CCCTTTCAT AAT-CAAGAG ACCTACAGAG TGAACCACAC ACCC--GACC
Canis_familiaris           CCTG-ACAAC -CAACT---T C-TTCATGAG AAGT-CAGAA AGAACCACAA ACCA--AACC
Felis_catus                ---T-AGAAA -TAATTCCAT C-TTCGTGAG AAGT-CTGCA AGAACCACAA ACCA--AACC
Ovis_aries                 ---------- ---------- ---------- ---------- ---------- ----------
Equus_caballus             CCTC-AGAAG -CAATT---- ----CAAGAG AAGCACAGAA AGAACCACAA ACCA--AACC
Tapirus_terrestris         ---------- ---------- ---------- ---------- ---------- ----------
Vicugna                    ---C-AGAAA -TAGTTTCAC C-TTCATGAG ATGTACAGAA AGAACCACAG AACA--AACC
Sus_scrofa                 CCTC-AGAAA -TAATTCCAT C-GTCATGAG ACATACGGAA AGAACCACAA ACCA--AACC
Bos_taurus                 CCTC-AGAAA -TAATTCCAC C-TTCATGAG AAGT----AA AGAACCACAA ACCA--AACC
Tursiops_truncatus         CCTC-AGAAA -TAATTCCAT C-ATCATGAG AAGTACAGAA AGAACCACAA ACCA--AACC
Erinaceus                  CTTCCAGA-- -TAATTCTAT C-TTCATCAG A-GTGCAAAA AGAACCACGA ACCA--GACC
Sorex_araneus              -CTC-AGAGC -TAATTCCA- TCTGCATGAG AAGTACAGAA AGAAACACAA ATCA--TACC
Neomys_anomalus            ---------- ---------- ---------- ---------- ---------- ----------
Choloepus_hoffmanni        CCTC-AGAAA -TGATTCCAT CATCCATGAG AAGTACAGAA AGAACCACAA GACA--AATC
Dasypus                    -CTC-AAAAA -TGACTCCAC CAT-CATGAG AAATACAGAA AGAACCACAA GGCA--AACC
```

```
                          61
Monodelphis               CCCCAAATGG TCAACCATAG AAAATACCCA TT---AAAAA AATT------ ----------
Macropus                  CCCCAACCAC TCAACAATAG AAAATACCGA TTT---AAAA AGTT------ ----------
Microcebus_murinus        TC-AAAATGG ACTCTAAGAG AAAATACCCT TT----ACAA AATT------ ----------
Tarsius_syrichta          GC-AAAACAG ATTTTGAGAG AGCATGCCTT TT---AGAAA ACTT------ ----------
Callithrix_jacchus        TC-CAAATTG ATTGTAAGAT AAGATATGCT T---AAAAAA AATT------ ----------
Macaca_mulatta            TC-CAAATCG ATTCTAAGAT AAAATACCCT TT---AAAAA AATT------ ----------
Pongo_pygmaeus            TC-CAAATTG ATTCTAAGAT AAAATACCTT T---AAAAAA AATT------ ----------
Gorilla_gorilla           TC-CAAATTG ATTCTAAGAT AAAATACCCT T---AAAAAA AATT------ ----------
Homo_sapiens              TC-CAAATTG ATTCTAAGAT AAAATACCCT T---AAAAAA AATT------ ----------
Pan_troglodytes           TC-CAAATTG ATTCTAAGAT AAAATACCCT T---AAAAAA AATT------ ----------
Ochotona_princeps         TC-AAAATGG ATTCTCTGAG AAAGTACCCT T---AAAAAA AATT------ ----------
Oryctolagus_cuniculus     TC-AAAATGG ATTCTATGAG AAAATACCCT T-----AAAA AAAT------ ----------
Marmota_monax             ---AAAATGG ATTCCAAGAG AAAATGCCCT TT---AAAAA AATT------ ----------
Aplodantia_rufa           ---AAAATGG ATTCCAAGAG AAAATACACT TT---AAAAA AATT------ ----------
Spermophilus              TC-AAAATGG ATTCCCAGAG AAAATGCCCT TTT---AAAA AATT------ ----------
Anomamorus                ---GAAATGG ATTTTAAGAG AAAACACCTT TTT-AAAAAA AGTT------ ----------
Maxomys                   ---AAAATAG ACTCCTTGAG AAACTACCCT TTCT-AAAAT AATT------ ----------
Rattus rattus             ---AAAACAG ACTCCCTGAG AAAATATCCT TTCT-AAAGT AACT------ ----------
Rattus exulans            ---------- ---------- ---------- ---------- ---------- ----------
Rattus_norvegicus         TC-AAAACAG ACTCCCTGAG AAAATATCCT TTCT-AAAAT AACT------ ----------
Mus_musculus              TC-AAAGTAG ACTCCCTGAG AAAATACCCT TTCT-AAATT AATT------ ----------
Mus_spretus               ---AAAGTAG ACTCCCTGAG AAAATACCCT TTCT-AAATT AATT------ ----------
Mus_pahari                ---------- ACTCCCTGAG AAAACACCCT TTCT--AATT AACG------ ----------
Praomys                   ---AAAATAG ACTCCCTGAG AAAATACCCT TACT-AAGTT AATT------ ----------
Meriones                  ------ATAG GCTCC-TGAG AAAATACCCT TTCT-AAAAT AATT------ ----------
Meriones_crassus          --------GG CCTCC-TGAG AA--TACCCT TTCT--AAAT AATT------ ----------
Acomys cahirinus          ---AAAATAG ACTCCCTGAG AAAATACCCT TTCT-AAAAT CATT------ ----------
Lophuromys sikapusi       ---AAAACGG ACTCCCTGAG AAAATACCCT TTCT-AAAAT CATT------ ----------
Mesocricetus_auratus      ---AGAATAG ATTCCCTGAG AAAATACCCT TTCT-AAAAT AATT------ ----------
Oryzomys                  ---AAAATAG ACTCC-TGAG AAAATACCCT TTCT-AAAAT AATT------ ----------
Trichys_fasciculata       ---ATAAGGG ACTCTAAGAG AAAATACCAT TT---AAAAA AATT------ ----------
Heterocephalus_glaber     ---AAAAGTG ACTC------ AAAATACCAC TTTT--AAAA ATTT------ ----------
Coendou_melanurus         ---AAAAGGG ACTCTAAGAG AAAATACCAT TTT---ACGA AGTA------ ----------
Cavia_porcellus           TC-AAAAGGT ACTCTAAGAG AAAATACCAT TTT---ACAA AGTT------ ----------
Ctenomys_maulinus         ---AAAAGGG CCTCTAAGGG AGAATACCTT TTT---GTAA AGTT------ ----------
Octodon_degus             ---AAAAGGG CCTCTAAGGG AAAATACCAT TTT---GCAA AGTT------ ----------
Loxodonta_africana        TC-CAGATGG ACTC-AAGAG AAAGTACCCT T--AAAAAAA AATT------ ----------
Procavia_capensis         TC-CAAATGG ACTC-AAGAA AAAATACCCT T-AAAAAAAA AATT------ ----------
Echinops_telfairi         GC--AGATAG ATT-TGAGAG AAAATTTACT TT---AAAAA ACTTCCTTTC AGGGCACATG
Micropotamogale           ---AAGCTGG ACT--TAGAG AAAATACCCT TT---AACAA AATC------ ----------
Myotis_lucifugus          TC-AGCTTCG ATTCTAAGAG AAAATACCC- ---AGAAAAA AATT------ ----------
Canis_familiaris          TC-AAAATAG ATTCTAAGAG AAAATACTCT T-AAAAAAAA AAAT------ ----------
Felis_catus               TC-AAAATAG ATTCGAAGAG AAAATACTCT T---AGAAAA AATT------ ----------
Ovis_aries                ---------- ---------- ---------- ---------- ---------- ----------
Equus_caballus            TC-GGAATGG ATTCTAAGTG AAAATACCTT TT-----AAA AAAT------ ----------
Tapirus_terrestris        ---ACTATGG ATTCTAAGAG AAAATACCTT T----GAAAA AATT------ ----------
Vicugna                   TC-AAAAAGG GCGCTAAGAG AAAATACCCT CT----AAAA ATTT------ ----------
Sus_scrofa                TCAAAAATTC ATGCTAAGAT AAAATACCCT TTAAAAAAAA AATT------ ----------
Bos_taurus                TC-AGAATGG ATGCT----- AAAACACCCT TT---AAAAA AATT------ ----------
Tursiops_truncatus        TC-AAAAAGG ATGCTAAGAG AAAACACCGT TT---AAAAA AATT------ ----------
Erinaceus                 TC-AAAATTG ATTGTCAGAG AAACTACCTT T-----AAAT TGTT------ ----------
Sorex_araneus             TC--GAATGC GTCGTCAGAG AAAATACCCT T---AAAAAA CGTT------ ----------
Neomys_anomalus           ---------- ---------- ---------- ---------- ---------- ----------
Choloepus_hoffmanni       TC-AAAATGG ATTCTAGGAG AAAATACCCT TT---AAAAA AATT------ ----------
Dasypus                   TC-AAAATGG ATTCTAGGAG AAAATACCCT ---AAAAAAG AATT------ ----------
```

```
                          121
Monodelphis               ---------- ---------- ---------- ---------- ---------- ----------
Macropus                  ---------- ---------- ---------- ---------- ---------- ----------
Microcebus_murinus        ---------- ---------- ---------- ---------- ---------- ----------
Tarsius_syrichta          ---------- ---------- ---------- ---------- ---------- ----------
Callithrix_jacchus        ---------- ---------- ---------- ---------- ---------- ----------
Macaca_mulatta            ---------- ---------- ---------- ---------- ---------- ----------
Pongo_pygmaeus            ---------- ---------- ---------- ---------- ---------- ----------
Gorilla_gorilla           ---------- ---------- ---------- ---------- ---------- ----------
Homo_sapiens              ---------- ---------- ---------- ---------- ---------- ----------
Pan_troglodytes           ---------- ---------- ---------- ---------- ---------- ----------
Ochotona_princeps         ---------- ---------- ---------- ---------- ---------- ----------
Oryctolagus_cuniculus     ---------- ---------- ---------- ---------- ---------- ----------
Marmota_monax             ---------- ---------- ---------- ---------- ---------- ----------
Aplodantia_rufa           ---------- ---------- ---------- ---------- ---------- ----------
Spermophilus              ---------- ---------- ---------- ---------- ---------- ----------
Anomamorus                ---------- ---------- ---------- ---------- ---------- ----------
Maxomys                   ---------- ---------- ---------- ---------- ---------- ----------
Rattus rattus             ---------- ---------- ---------- ---------- ---------- ----------
Rattus exulans            ---------- ---------- ---------- ---------- ---------- ----------
Rattus_norvegicus         ---------- ---------- ---------- ---------- ---------- ----------
Mus_musculus              ---------- ---------- ---------- ---------- ---------- ----------
Mus_spretus               ---------- ---------- ---------- ---------- ---------- ----------
Mus_pahari                ---------- ---------- ---------- ---------- ---------- ----------
Praomys                   ---------- ---------- ---------- ---------- ---------- ----------
Meriones                  ---------- ---------- ---------- ---------- ---------- ----------
Meriones_crassus          ---------- ---------- ---------- ---------- ---------- ----------
Acomys cahirinus          ---------- ---------- ---------- ---------- ---------- ----------
Lophuromys sikapusi       ---------- ---------- ---------- ---------- ---------- ----------
Mesocricetus_auratus      ---------- ---------- ---------- ---------- ---------- ----------
Oryzomys                  ---------- ---------- ---------- ---------- ---------- ----------
Trichys_fasciculata       ---------- ---------- ---------- ---------- ---------- ----------
Heterocephalus_glaber     ---------- ---------- ---------- ---------- ---------- ----------
Coendou_melanurus         ---------- ---------- ---------- ---------- ---------- ----------
Cavia_porcellus           ---------- ---------- ---------- ---------- ---------- ----------
Ctenomys_maulinus         ---------- ---------- ---------- ---------- ---------- ----------
Octodon_degus             ---------- ---------- ---------- ---------- ---------- ----------
Loxodonta_africana        ---------- ---------- ---------- ---------- ---------- ----------
Procavia_capensis         ---------- ---------- ---------- ---------- ---------- ----------
Echinops_telfairi         GAATCCAGGA ACAGAGATGG GAGTAGCGAT ACCAGGACTG TAGGGGAAAG GAGGGAAAGG
Micropotamogale           ---------- ---------- ---------- ---------- ---------- ----------
Myotis_lucifugus          ---------- ---------- ---------- ---------- ---------- ----------
Canis_familiaris          ---------- ---------- ---------- ---------- ---------- ----------
Felis_catus               ---------- ---------- ---------- ---------- ---------- ----------
Ovis_aries                ---------- ---------- ---------- ---------- ---------- ----------
Equus_caballus            ---------- ---------- ---------- ---------- ---------- ----------
Tapirus_terrestris        ---------- ---------- ---------- ---------- ---------- ----------
Vicugna                   ---------- ---------- ---------- ---------- ---------- ----------
Sus_scrofa                ---------- ---------- ---------- ---------- ---------- ----------
Bos_taurus                ---------- ---------- ---------- ---------- ---------- ----------
Tursiops_truncatus        ---------- ---------- ---------- ---------- ---------- ----------
Erinaceus                 ---------- ---------- ---------- ---------- ---------- ----------
Sorex_araneus             ---------- ---------- ---------- ---------- ---------- ----------
Neomys_anomalus           ---------- ---------- ---------- ---------- ---------- ----------
Choloepus_hoffmanni       ---------- ---------- ---------- ---------- ---------- ----------
Dasypus                   ---------- ---------- ---------- ---------- ---------- ----------
```

```
                         181
Monodelphis              ---------- ---------- ---------- ---------- ---------- ----------
Macropus                 ---------- ---------- ---------- ---------- ---------- ----------
Microcebus_murinus       ---------- ---------- ---------- ---------- ---------- ----------
Tarsius_syrichta         ---------- ---------- ---------- ---------- ---------- ----------
Callithrix_jacchus       ---------- ---------- ---------- ---------- ---------- ----------
Macaca_mulatta           ---------- ---------- ---------- ---------- ---------- ----------
Pongo_pygmaeus           ---------- ---------- ---------- ---------- ---------- ----------
Gorilla_gorilla          ---------- ---------- ---------- ---------- ---------- ----------
Homo_sapiens             ---------- ---------- ---------- ---------- ---------- ----------
Pan_troglodytes          ---------- ---------- ---------- ---------- ---------- ----------
Ochotona_princeps        ---------- ---------- ---------- ---------- ---------- ----------
Oryctolagus_cuniculus    ---------- ---------- ---------- ---------- ---------- ----------
Marmota_monax            ---------- ---------- ---------- ---------- ---------- ----------
Aplodantia_rufa          ---------- ---------- ---------- ---------- ---------- ----------
Spermophilus             ---------- ---------- ---------- ---------- ---------- ----------
Anomamorus               ---------- ---------- ---------- ---------- ---------- ----------
Maxomys                  ---------- ---------- ---------- ---------- ---------- ----------
Rattus rattus            ---------- ---------- ---------- ---------- ---------- ----------
Rattus exulans           ---------- ---------- ---------- ---------- ---------- ----------
Rattus_norvegicus        ---------- ---------- ---------- ---------- ---------- ----------
Mus_musculus             ---------- ---------- ---------- ---------- ---------- ----------
Mus_spretus              ---------- ---------- ---------- ---------- ---------- ----------
Mus_pahari               ---------- ---------- ---------- ---------- ---------- ----------
Praomys                  ---------- ---------- ---------- ---------- ---------- ----------
Meriones                 ---------- ---------- ---------- ---------- ---------- ----------
Meriones_crassus         ---------- ---------- ---------- ---------- ---------- ----------
Acomys cahirinus         ---------- ---------- ---------- ---------- ---------- ----------
Lophuromys sikapusi      ---------- ---------- ---------- ---------- ---------- ----------
Mesocricetus_auratus     ---------- ---------- ---------- ---------- ---------- ----------
Oryzomys                 ---------- ---------- ---------- ---------- ---------- ----------
Trichys_fasciculata      ---------- ---------- ---------- ---------- ---------- ----------
Heterocephalus_glaber    ---------- ---------- ---------- ---------- ---------- ----------
Coendou_melanurus        ---------- ---------- ---------- ---------- ---------- ----------
Cavia_porcellus          ---------- ---------- ---------- ---------- ---------- ----------
Ctenomys_maulinus        ---------- ---------- ---------- ---------- ---------- ----------
Octodon_degus            ---------- ---------- ---------- ---------- ---------- ----------
Loxodonta_africana       ---------- ---------- ---------- ---------- ---------- ----------
Procavia_capensis        ---------- ---------- ---------- ---------- ---------- ----------
Echinops_telfairi        AGGGGAGGAA GGGGAAACGG GTCCCAATTA AAACAAAAAA CAAGACAAAA CAAAAAAAAC
Micropotamogale          ---------- ---------- ---------- ---------- ---------- ----------
Myotis_lucifugus         ---------- ---------- ---------- ---------- ---------- ----------
Canis_familiaris         ---------- ---------- ---------- ---------- ---------- ----------
Felis_catus              ---------- ---------- ---------- ---------- ---------- ----------
Ovis_aries               ---------- ---------- ---------- ---------- ---------- ----------
Equus_caballus           ---------- ---------- ---------- ---------- ---------- ----------
Tapirus_terrestris       ---------- ---------- ---------- ---------- ---------- ----------
Vicugna                  ---------- ---------- ---------- ---------- ---------- ----------
Sus_scrofa               ---------- ---------- ---------- ---------- ---------- ----------
Bos_taurus               ---------- ---------- ---------- ---------- ---------- ----------
Tursiops_truncatus       ---------- ---------- ---------- ---------- ---------- ----------
Erinaceus                ---------- ---------- ---------- ---------- ---------- ----------
Sorex_araneus            ---------- ---------- ---------- ---------- ---------- ----------
Neomys_anomalus          ---------- ---------- ---------- ---------- ---------- ----------
Choloepus_hoffmanni      ---------- ---------- ---------- ---------- ---------- ----------
Dasypus                  ---------- ---------- ---------- ---------- ---------- ----------
```

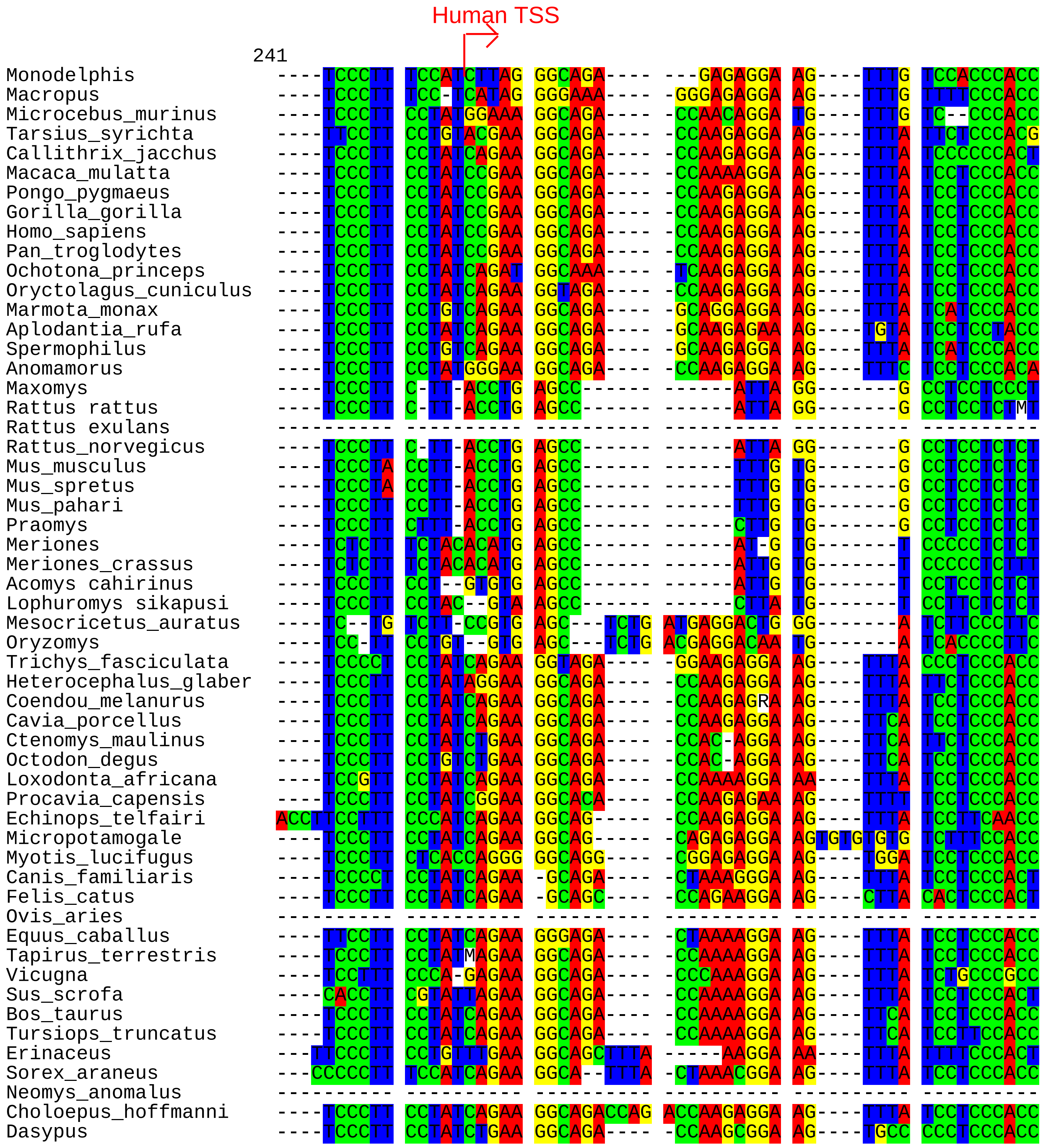
Human TSS
241
Monodelphis
Macropus
Microcebus_murinus
Tarsius_syrichta
Callithrix_jacchus
Macaca_mulatta
Pongo_pygmaeus
Gorilla_gorilla
Homo_sapiens
Pan_troglodytes
Ochotona_princeps
Oryctolagus_cuniculus
Marmota_monax
Aplodantia_rufa
Spermophilus
Anomamorus
Maxomys
Rattus rattus
Rattus exulans
Rattus_norvegicus
Mus_musculus
Mus_spretus
Mus_pahari
Praomys
Meriones
Meriones_crassus
Acomys cahirinus
Lophuromys sikapusi
Mesocricetus_auratus
Oryzomys
Trichys_fasciculata
Heterocephalus_glaber
Coendou_melanurus
Cavia_porcellus
Ctenomys_maulinus
Octodon_degus
Loxodonta_africana
Procavia_capensis
Echinops_telfairi
Micropotamogale
Myotis_lucifugus
Canis_familiaris
Felis_catus
Ovis_aries
Equus_caballus
Tapirus_terrestris
Vicugna
Sus_scrofa
Bos_taurus
Tursiops_truncatus
Erinaceus
Sorex_araneus
Neomys_anomalus
Choloepus_hoffmanni
Dasypus

```
                          301
Monodelphis               CACAGTTTCC C-CAGA---- GGTG-----T TTCATCTGGG C---TGTTT- TATTTTCCCT
Macropus                  CACAGTTTCC C-CAGA---- GATG-----G TTCATCTATG C---TGTTT- TAATTTCCCT
Microcebus_murinus        CACAAATTTC C-CAAA---- TAAC-----T TTTATATAGA G---GGTTT- CACTCGGATT
Tarsius_syrichta          CACACATTTC C-CAGA---- GAGT-----T TTCATCTAGA G---GGTTT- TGCGCTGCTG
Callithrix_jacchus        CACAAATTCT CACGGA---- GAGC----TT TTCATCTAGA A---GGTTT- GACTCCGGTT
Macaca_mulatta            CACAAATTCC CCCAGA---- GAGC-----T TTCATCTAGA A---GGTTT- GACTCTGGCT
Pongo_pygmaeus            TACAAATTCC C-CAGA---- GAGC-----T TTCATCTAGA A---GGTTT- GACTCTGGCT
Gorilla_gorilla           TACAAATTCC C-CAGA---- GAGC-----T TTCATCTAGA A---GGTTT- GACTCTGGCC
Homo_sapiens              TACAAATTCC C-CAGA---- GAGC-----T TTCATCTAGA A---GGTTT- GACTCTGGCC
Pan_troglodytes           TACAAATTCC C-CAGA---- GAGC-----T TTCATCTAGA A---GGTTT- GACTCTGGCC
Ochotona_princeps         CACAAATTCA C-CAGG---- --CC-----T TTCATCTAGA A---GGTTT- GGCTCTTGCT
Oryctolagus_cuniculus     CACAAATTTG C---AA---- AAGC-----T TTTGTCTTCA A---GTTTT- TGCTGTGGTT
Marmota_monax             AGCAAATTTC C-CAGA---- AAGG-----T TTCATTTACT C---GGGTG- --------GAC
Aplodantia_rufa           TGCAAACTTC C-TGGA---- AAGG-----T TTCATTTAGG A---GGCTT- TGCCACGCTC
Spermophilus              AGCAAATTTC C-CCGA---- AAAA-----T TTCATTAACT C---GGGTG- --------GAC
Anomamorus                CACAAGTTCC C-CAGA---- GAGT------ --TGGCCAGC A---GACCC- GG--------
Maxomys                   GAAGACCC-- ---------- ---------- ---------- ---------- ----------
Rattus rattus             GAAGACCC-- ---------- ---------- ---------- ---------- ----------
Rattus exulans            ---------- ---------- ---------- ---------- ---------- ----------
Rattus_norvegicus         GAAGACCC-- ---------- ---------- ---------- ---------- ----------
Mus_musculus              AAAGACCC-- ---------- ---------- ---------- ---------- ----------
Mus_spretus               AAAGACCC-- ---------- ---------- ---------- ---------- ----------
Mus_pahari                GAAGACCC-- ---------- ---------- ---------- ---------- ----------
Praomys                   GAAGACCC-- ---------- ---------- ---------- ---------- ----------
Meriones                  GAGGACCTGC ---------- ---------- ---------- ---------- ----------
Meriones_crassus          GAGGACCTGC ---------- ---------- ---------- ---------- ----------
Acomys cahirinus          GAAGACCCGG GTT------- ---------- ---------- ---------- ----------
Lophuromys sikapusi       GAAGACCTGG GTT------- ---------- ---------- ---------- ----------
Mesocricetus_auratus      CGCACAGTCC C-ACATTTTA TTGCAGCCTT TTCTCTGAAG ACCCAGTCCC ACCCCATAAT
Oryzomys                  CACACAGGCT C-AAGTTTTA TTGT-GGCTT ------CTAG TCTCAT---- ----------
Trichys_fasciculata       GACAAATTCC C-CAGA---- GAGCTGAACT TTGGGCTAGG A---AGTTT- TACTCTGGTC
Heterocephalus_glaber     CACAAATTCC C-CAGA---- AAGCTGAACT TTTGGCTAAA A---GGTTT- TCCTCTGGTC
Coendou_melanurus         -GCGAATTCC C-CAGAGCT- GAGC-----T TTGGGCTAGA A---GGTTG- TACTTTGGTC
Cavia_porcellus           -ACGAGTTTC T-TAGAGCT- GAAC-----T TCTGGCTAGA A---GGTCT- ----TTGGTC
Ctenomys_maulinus         -ACGAATTCC C-CAGA---- GATCTGAGGT TTTGGCTACA A---GGTTT- TGCTCTGGTC
Octodon_degus             -ACGAATTCC C-CAGA---- GAGCTGAGCT TTTGGCTAGA A---GGTTT- TGCTCTGGTC
Loxodonta_africana        CACAAATTCC C-CAGT---- AAGC-----T TTCATCTAGC G---GTTTT- CACTCTCTTT
Procavia_capensis         TACAAATTCC C-CAGT---- AAGC-----T TTCAACTAGA G---ATTTT- TACTCTCTTT
Echinops_telfairi         TGCAAATTCC C-CAGT---- AAGC-----T TTCAGCTA-A A---GGTTT- TACACTCTTT
Micropotamogale           CACAAATTCC C-CGGTGAAA AAGC-----G TCCATCTG-A A---GGTTG- TACCTTTCAG
Myotis_lucifugus          CACGAATTTC CCCGGA---- AAGC-----G TTCGTCTGGG A---GGTAC- TGTTCTCCTT
Canis_familiaris          TACAAATTCC C-CAGA---- AAGC------ TTCACCTAGA A---GGTTG- TGCTCTCTTC
Felis_catus               TACAAGTTCC C-CAGA---- AAGC------ TTCATCTAGA A---GGTTGT TACTTTCTTC
Ovis_aries                ---------- ---------- ---------- ---------- ---------- ----------
Equus_caballus            CACAAATTCC C-CAGA---- AACC-----T TCCATCTAGA A---GGTTT- TGCTCTCTTT
Tapirus_terrestris        CACAAATTCC C-CAGA---- AAGC-----T TTCATTTAGA A---CGTT-T TGCACTCTTT
Vicugna                   CGCAAATTCC C-CAGA---- GAGC-----T TTCCACCAGA A---GGTT-- ----------
Sus_scrofa                GACAAATTCC C-CAGA---- GACC-----T TTCTACCAGA A---GGTT-- ----------
Bos_taurus                CACAAATTCC C-CAGC---- AAGC-----T CTCCATCAGA A---GGTCC- ----------
Tursiops_truncatus        CACAGATTCC C-CAGA---- GAGC-----T TTCCACCGGA A---GGTC-- ----------
Erinaceus                 CACAAATTTC C-CAGA---- AAAT-----T TTCATGTAGC A---GATTT- TATTATTTTT
Sorex_araneus             CACAAATTTC C-CAGA---- ---------C CTTTTCATCG A---GATTG- TATTCTCTCT
Neomys_anomalus           ---------- ---------- ---------- ---------- ---------- ----------
Choloepus_hoffmanni       CACAAATTCC C-CTGA---- GAGC-----T TTCATCT--- A---GGTTT- TACTCTCTTT
Dasypus                   TGCTAATTTC C--AGA---- GAGC-----T TTCATCTAGA A---GGTT-- GGACCTCTTT
```

```
                          361
Monodelphis               ACACAGAGGA TCTGTTCCAT TTTCAA---- ---AGCCTCA T--------- ----------
Macropus                  ACTCAGAGGA TTTTTTCCAT TTTTAA---- ---AGCCTCA T--------- ----------
Microcebus_murinus        AGACACACAG CAAGCG---- ---------- ----CCTTCT T--------- ----------
Tarsius_syrichta          AGACATCCAG CCAGCATGAG ACATCCAGCC AGCATCTTCT T--------- ----------
Callithrix_jacchus        AGAT----AC CCAGCG---- ---------- ---AGCATCT T--------- ----------
Macaca_mulatta            AGAC----AA CCAGCG---- ---------- ---AGCATCT T--------- ----------
Pongo_pygmaeus            AGAC----AA CCAGTG---- ---------- ---AGCATCT T--------- ----------
Gorilla_gorilla           AGAC----AA CCAGCG---- ---------- ---AGCATCT T--------- ----------
Homo_sapiens              AGAC----AA CCAGCG---- ---------- ---AGCATCT T--------- ----------
Pan_troglodytes           AGAC----AA CCAGCG---- ---------- ---AGCATCT T--------- ----------
Ochotona_princeps         GGGCGCACAG TGAGC----- ---------- ---ACACTCC T--------- ----------
Oryctolagus_cuniculus     CAGC----AT ACAGTG---- ---------- ---AGCACCC T--------- ----------
Marmota_monax             ACAC---AAG CCAGCA---- ---------- ---ATCCTCT T--------- ----------
Aplodantia_rufa           AGCCACACAG CCAGC----- ---------- ---ACCCTCT CCTGAGAAAC CCTGGGGACT
Spermophilus              ACAC----AG CCAGCA---- ---------- ---ATCCTCT T--------- ----------
Anomamorus                ---------G CCAGC----- ---------- ---ATCCTCT C--------- ----------
Maxomys                   ---------- ---------- ---------- ---------- ---------- ----------
Rattus rattus             ---------- ---------- ---------- ---------- ---------- ----------
Rattus exulans            ---------- ---------- ---------- ---------- ---------- ----------
Rattus_norvegicus         ---------- ---------- ---------- ---------- ---------- ----------
Mus_musculus              ---------- ---------- ---------- ---------- ---------- ----------
Mus_spretus               ---------- ---------- ---------- ---------- ---------- ----------
Mus_pahari                ---------- ---------- ---------- ---------- ---------- ----------
Praomys                   ---------- ---------- ---------- ---------- ---------- ----------
Meriones                  ---------- ---------- ---------- ---------- ---------- ----------
Meriones_crassus          ---------- ---------- ---------- ---------- ---------- ----------
Acomys cahirinus          ---------- ---------- ---------- ---------- ---------- ----------
Lophuromys sikapusi       ---------- ---------- ---------- ---------- ---------- ----------
Mesocricetus_auratus      GCCCACATAC CCCAC----- ---------- ---AAACACT T--------- ----------
Oryzomys                  ---------- ---------- ---------- ---ACCTCCT C--------- ----------
Trichys_fasciculata       AGCCGCGCAG CCAGC----- ---------- ---AGCCTCT C--------- ----------
Heterocephalus_glaber     ACAC--ACAG TCAGT----- ---------- ---ACCCTGG T--------- ----------
Coendou_melanurus         CCAC-CGTAG CCAGC----- ---------- ---ACCCTCT T--------- ----------
Cavia_porcellus           CCACGCAGCA CTAGC----- ---------- ---ACCCTCT T--------- ----------
Ctenomys_maulinus         CCACACACAC CCAGC----- ---------- ---ACACTCT TCTCTGGTAC TGGGGATTGA
Octodon_degus             CCACGCACAG ACAGC----- ---------- ---ACCCTCT T--------- ----------
Loxodonta_africana        AGACAGGAAG CGGGC----- ---------- ---ACCCTCT T--------- ----------
Procavia_capensis         GCACAGGAAG CGGGC----- ---------- ---ATCCTCT T--------- ----------
Echinops_telfairi         AACCAGGCAG TGGGT----- ---------- ---ACCTTCT T--------- ----------
Micropotamogale           AATTAGACCG TGGGC----- ---------- ---AAGTTCT T--------- ----------
Myotis_lucifugus          AGACACAGGG CGGG------ ---------- ---ACCCTGC T--------- ----------
Canis_familiaris          AGACAC-TAG CAGG------ ---------- ---ACCCTCT T--------- ----------
Felis_catus               AGACACACAG CGGG------ ---------- ---TCACTCT T--------- ----------
Ovis_aries                ---------- ---------- ---------- ---------- ---------- ----------
Equus_caballus            AGACACCCGG CAGG------ ---------- ---ACCCTCT T--------- ----------
Tapirus_terrestris        AAACAC--AG CARG------ ---------- ---GCCCTCT T--------- ----------
Vicugna                   -GACACCCAG TTTG------ ---------- ---ACCCTCT T--------- ----------
Sus_scrofa                -GACACACAG CTCC------ ---------- ---ACCATCT T--------- ----------
Bos_taurus                --ACATTCAG CTTG------ ---------- ---ACCCTCT T--------- ----------
Tursiops_truncatus        -GACAGCCAG CCGG------ ---------- ---ACCCTCT T--------- ----------
Erinaceus                 AGACACAGAG CAGG------ ---------- ---GTCCTCT T--------- ----------
Sorex_araneus             TTACACACAG CAGG------ ---------- ---GTCTTCT ---------- ----------
Neomys_anomalus           ---------- ---------- ---------- ---------- ---------- ----------
Choloepus_hoffmanni       AGGTACACAG TGGGC----- ---------- ---ACCCTCT T--------- ----------
Dasypus                   ACACCCTCGG CAGGC----- ---------- ---ATCCTCT T--------- ----------
```

```
                         421
Monodelphis              ---------- ---------- ---------- ---------- -------CTC TGCGTTTAAC
Macropus                 ---------- ---------- ---------- ---------- -------CTC TTTGATTAAT
Microcebus_murinus       ---------- ---------- ---------- ---------- -------CTC TGAACC----
Tarsius_syrichta         ---------- ---------- ---------- ---------- -------CTT TGAACC----
Callithrix_jacchus       ---------- ---------- ---------- ---------- -------CTC TGAACC----
Macaca_mulatta           ---------- ---------- ---------- ---------- -------CTT GGAACC----
Pongo_pygmaeus           ---------- ---------- ---------- ---------- -------CTC CCAATC----
Gorilla_gorilla          ---------- ---------- ---------- ---------- -------CTC GCAATC----
Homo_sapiens             ---------- ---------- ---------- ---------- -------CTC GCAATC----
Pan_troglodytes          ---------- ---------- ---------- ---------- -------CTC GCAATC----
Ochotona_princeps        ---------- ---------- ---------- ---------- -------CTC TGAACC----
Oryctolagus_cuniculus    ---------- ---------- ---------- ---------- -------CT- ----TC----
Marmota_monax            ---------- ---------- ---------- ---------- -------CTC TGAAGC----
Aplodantia_rufa          TCCATGGCAT ATCCCAGAGA ATAAGGAGCC A--------- -------CTC T---------
Spermophilus             ---------- ---------- ---------- ---------- -------CTC TGAAGC----
Anomamorus               ---------- ---------- ---------- ---------- -------CTC TGAAAC----
Maxomys                  ---------- ---------- ---------- ---------- ---------- ----------
Rattus rattus            ---------- ---------- ---------- ---------- ---------- ----------
Rattus exulans           ---------- ---------- ---------- ---------- ---------- ----------
Rattus_norvegicus        ---------- ---------- ---------- ---------- ---------- ----------
Mus_musculus             ---------- ---------- ---------- ---------- ---------- ----------
Mus_spretus              ---------- ---------- ---------- ---------- ---------- ----------
Mus_pahari               ---------- ---------- ---------- ---------- ---------- ----------
Praomys                  ---------- ---------- ---------- ---------- ---------- ----------
Meriones                 ---------- ---------- ---------- ---------- ---------- ----------
Meriones_crassus         ---------- ---------- ---------- ---------- ---------- ----------
Acomys cahirinus         ---------- ---------- ---------- ---------- ---------- ----------
Lophuromys sikapusi      ---------- ---------- ---------- ---------- ---------- ----------
Mesocricetus_auratus     ---------- ---------- ---------- ---------- ---------- ----------
Oryzomys                 ---------- ---------- ---------- ---------- ---------- ----------
Trichys_fasciculata      ---------- ---------- ---------- ---------- -------CTC TGAACT----
Heterocephalus_glaber    ---------- ---------- ---------- ---------- -------CTC TAAACT----
Coendou_melanurus        ---------- ---------- ---------- ---------- -------CTC TGAACC----
Cavia_porcellus          ---------- ---------- ---------- ---------- -------CTC CGAAGC----
Ctenomys_maulinus        ACTCAGGACC CTACACTTCC CAGGCTGCTC TGAGCTTCTT GCGTATTCCC TGAACT----
Octodon_degus            ---------- ---------- -------CTC TGAGCTTCTT GTGTCTTCCC TAAACT----
Loxodonta_africana       ---------- ---------- ---------- ---------- -------CTC TGAACC----
Procavia_capensis        ---------- ---------- ---------- ---------- -------CTC TGAACC----
Echinops_telfairi        ---------- ---------- ---------- ---------- -------CTT TGAACCACAT
Micropotamogale          ---------- ---------- ---------- ---------- -------TTC TGAACG----
Myotis_lucifugus         ---------- ---------- ---------- ---------- -------CCG GGTGCC----
Canis_familiaris         ---------- ---------- ---------- ---------- -------TTC TGAGCC----
Felis_catus              ---------- ---------- ---------- ---------- -------TTC TGAGGC----
Ovis_aries               ---------- ---------- ---------- ---------- ---------- -GTGCC----
Equus_caballus           ---------- ---------- ---------- ---------- -------CTC TGAGCC----
Tapirus_terrestris       ---------- ---------- ---------- ---------- -------CTC TGAGTC----
Vicugna                  ---------- ---------- ---------- ---------- -------CTC TGAGCC----
Sus_scrofa               ---------- ---------- ---------- ---------- -------CTC TGAGCC----
Bos_taurus               ---------- ---------- ---------- ---------- -------CTC TGAGCC----
Tursiops_truncatus       ---------- ---------- ---------- ---------- -------CTC TGAGCC----
Erinaceus                ---------- ---------- ---------- ---------- -------ACC TGAGTC----
Sorex_araneus            ---------- ---------- ---------- ---------- -------ACG AGAGCC----
Neomys_anomalus          ---------- ---------- ---------- ---------- ---------- ----------
Choloepus_hoffmanni      ---------- ---------- ---------- ---------- -------CTC TTG-------
Dasypus                  ---------- ---------- ---------- ---------- -------CTC TGAA------
```

```
                       481
Monodelphis            A---TATTA- -----TTTCA T-CCATGGCA ---CATTT-C TGAGACTTAA GAAGA-----
Macropus               G---TATTG- -----TTGCA T-CCATGGCA ---GAGTT-C TGAGACTTAA GAAGA-----
Microcebus_murinus     ----TCATA- -CGTATTTCT T-CCTTGGCA ---TATTC-C AGAGAA-TAA -ATGC-----
Tarsius_syrichta       ----TTTTG- -----CTTCT T-CCGTGGCA ---TATTC-C AGAGAA-TAG GGAGC-----
Callithrix_jacchus     ----TCTTG- -----TTTCT T-CCATGGCA ---AACTC-C AGAGAA-TAA GAAGC-----
Macaca_mulatta         ----TCTTG- -----CTTCT T-CCATGGCA ---AACTC-C AGAGAA-TAA GAAGC-----
Pongo_pygmaeus         ----TGTTG- -----CTTCT T-CCATGGCA ---AACTC-C AGAGAATTAA GAAGC-----
Gorilla_gorilla        ----TGTTG- -----CTTCT T-CCATGGCA ---AACTC-C AGAGAATTAA GAAGC-----
Homo_sapiens           ----TGTTG- -----CTTCT T-CCATGGCA ---AACTC-C AGAGAATTAA GAAGC-----
Pan_troglodytes        ----TGTTG- -----CTTCT T-CCATGGCA ---AACTC-C AGAGAATTAA GAAGC-----
Ochotona_princeps      ----TCTTT- -----CTTCC --CTGCGACG ---CA----C AGAGAA-TAA GACAG-----
Oryctolagus_cuniculus  ----TCTTGA ACGTATTTCT T-CCATGGAA ---AATTC-C AGAGAA-TAA GACGG-----
Marmota_monax          ---------- -------TCT T-CTATGGCG ---TACTC-T GGAGAA-TAA GGAG------
Aplodantia_rufa        ---------- ---------- ---------- ---------- ---------- ----------
Spermophilus           ---------- -------TCT T-CTATGGCG ---TACTC-C GGAGAA-TAA GGAG------
Anomamorus             ----TCTTT- ----GCTTCT T-CCATGTTA ---TATTC-C AGAGAA-CAA GAAGC-----
Maxomys                ---------- -----AGTTT TTCCATGGCA ---TCATA-T GGAGAA-TAC AAGCC-----
Rattus rattus          ---------- -----ACTTT TCCCATGGCA ---TCTTA-T GGAGAA-TAC AAGCC-----
Rattus exulans         ---------- ---------- ---------- ---------- ---------- ----------
Rattus_norvegicus      ---------- -----ACTTT TTCCATGGCA ---TCTTA-T GGAGAA-TAC AAGCC-----
Mus_musculus           ---------- ----AACTCT T-CCATGGCA GCATCTTA-T GGAGAA-TAC AAACC-----
Mus_spretus            ---------- ----AACTCT T-CCATGGCA GCATCTTA-T GGAGAA-TAC AAACC-----
Mus_pahari             ---------- ----AACTCT T-CCATGGCA ---TCTTA-T GGAGAA-TAC AAACC-----
Praomys                ---------- ----AATTTT T-CTGTGGCA ---TCTTA-T GGATAA-TAC AAACC-----
Meriones               ---------- -------TTT TTCCATGGCA ---TCA---C GAGGAA-TAA AAATG-----
Meriones_crassus       ---------- -------TTT TTCCATGGCA ---TCA---T GAGGAA-TAA AAATG-----
Acomys cahirinus       ---------- ---------T T-CCACGGCA ---TCTTA-T GAAGAA-TAA AAATA-----
Lophuromys sikapusi    ---------- ---------T T-CCGTGGCA ---TCTAA-T GAAGAA-TAA AAATA-----
Mesocricetus_auratus   ---------- ------TCTT TT-------- ---------- ---------- -TCCAGGCTG
Oryzomys               ---------- ------CCT- -CAC-----A CACATTT-TT AAAATATAAA GTTCAGGCTG
Trichys_fasciculata    ----TCTTG- -----CCTCT T-CCATGGAA ---TATTT-C AGAGAC-TAA GAAGC-----
Heterocephalus_glaber  ----TCTCG- -----CCTCT T-TCATGGAG ---TATTT-C AGAGAC-TAA GAAGC-----
Coendou_melanurus      ----TCTTG- -----CCTCT T-CCATGGAC ---CATTT-C AGAGAG-TAA GAAGC-----
Cavia_porcellus        ----TCTTG- -----CCTCT T-TCCTGGAA ---TATTT-C AGAGAT-TAA GAAG------
Ctenomys_maulinus      ----TCTTG- -----CCTCT T-CCACAGAC ---TATTT-C AGAGAT-TAA GAAGC-----
Octodon_degus          ----TCTTG- -----CCTAT T-CCACGTAT ---TAATT-C AGAGAT-TAA GAAGC-----
Loxodonta_africana     ----ACTTG- -----TTTCT T-CAATGGCA ---CATTT-C AGAGAA-TAA GACAC-----
Procavia_capensis      ----ACCTG- -----TTTCT T-CA--GAC- ---TGTTT-T AGAGAC-TAA GACGC-----
Echinops_telfairi      TTTTTTTTG- -----TTAAT TTCAGGGGCA ---CAGTT-T GAAGAA-CAA TATAC-----
Micropotamogale        ----GCTTGG TTTTTCCTCT TTCC------ ---------C GAAGAA-CAA GAGTT-----
Myotis_lucifugus       ----TCATG- -------TCT T--CATAGCA ---CATTT-C AGAGAA-TAA --AGC-----
Canis_familiaris       ----TGTTA- -----TTTCT T-CCGTGGCA ---TGTTC-C AGAGAA-TAA GACCC-----
Felis_catus            ----TCTTG- -----CCTCT T-CCATGGCC ---TCTTC-C AGAGAC-TAA GACAC-----
Ovis_aries             ----TCTAG- -----TTCCT CTCCATGGCA ---TATTC-T AGAGAA-TAA GGCAACGCGT
Equus_caballus         ----TCTTG- -----CTGCT T-CCATGGCA ---CATTG-C AGAGAG-TAA GACGC-----
Tapirus_terrestris     ----TCTTG- -----TTTCT T-CCATGGCA ---TATTG-C AGAGAG-TAA GATGC-----
Vicugna                ----TCTTG- -----TTTCT T-CCATGTTA ---TACTC-C AGAGAA-TAA GCTGC-----
Sus_scrofa             ----TCCTG- -----TGCTC T-CCTTGGCA ---TATGC-C AGAGAG-TAA GGGGC-----
Bos_taurus             ----TCTAG- -----TTCCG C-CCATGGCA ---TATTC-T AGAGAA-TAA GGCAA-----
Tursiops_truncatus     ----TCCCG- -----TTTCT T-TCATGGCA ---TA----- -------TTA AGTGC-----
Erinaceus              ----TCTTG- -----TTTCT C-CCAGGGTA ---TATTC-T GGAAG--TGA TG--------
Sorex_araneus          ----TCTGA- -----TTTCT T-CCATGGCT ---AGCTC-G GGAGAA-TAA GAAGG-----
Neomys_anomalus        ---------- ---------- ---------- ---------G GGAGAA-TAA GAAGG-----
Choloepus_hoffmanni    ---------- -----TTTCT T-CCACAGGA ---TATTT-C AGAAAA-TAA GACAC-----
Dasypus                ---------- -----CCCCT T-CCATGGGC ---TGTTT-C AGAGAA-TAA GACTC-----
```

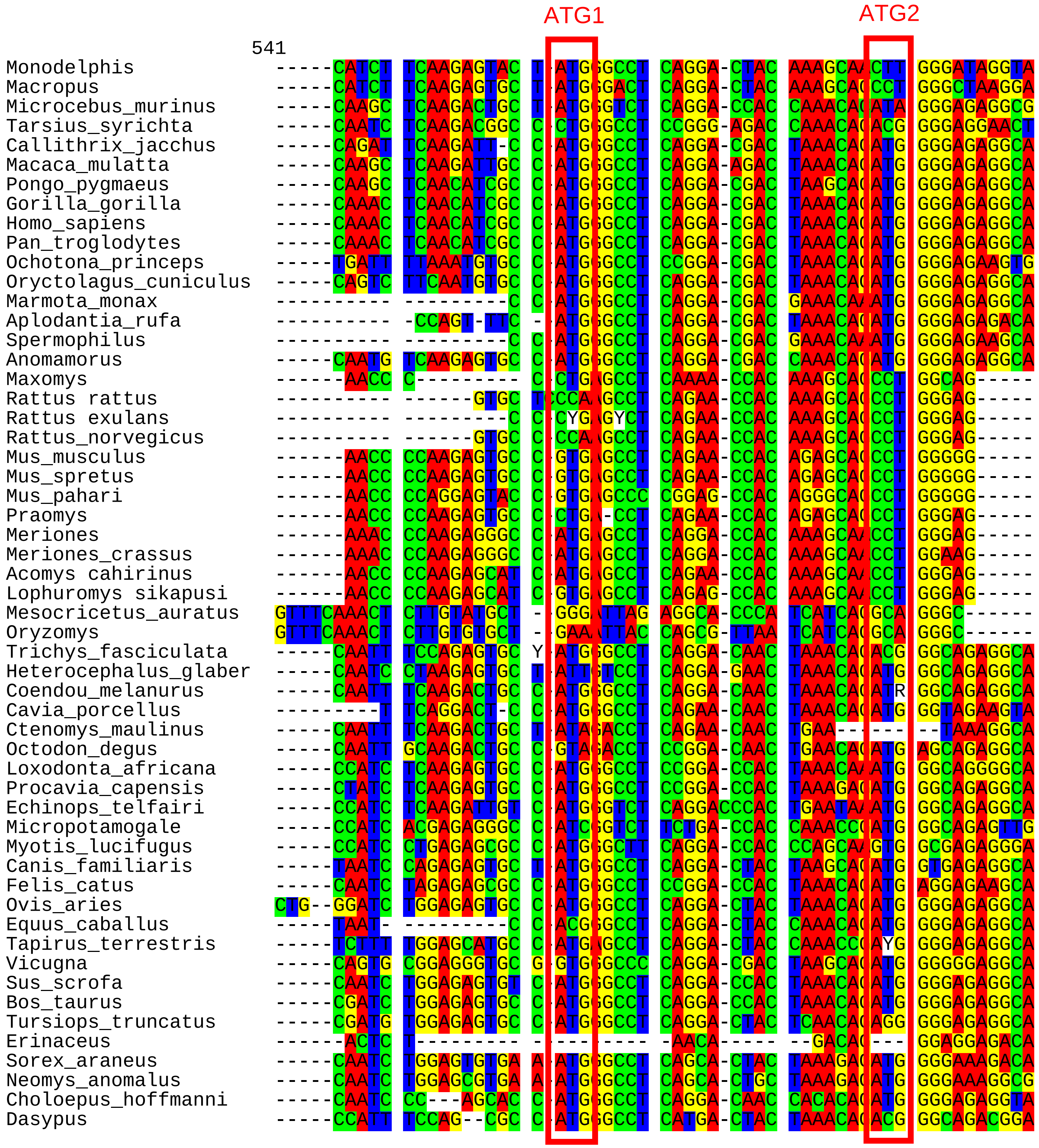
ATG1
ATG2
541
Monodelphis
Macropus
Microcebus_murinus
Tarsius_syrichta
Callithrix_jacchus
Macaca_mulatta
Pongo_pygmaeus
Gorilla_gorilla
Homo_sapiens
Pan_troglodytes
Ochotona_princeps
Oryctolagus_cuniculus
Marmota_monax
Aplodantia_rufa
Spermophilus
Anomamorus
Maxomys
Rattus rattus
Rattus exulans
Rattus_norvegicus
Mus_musculus
Mus_spretus
Mus_pahari
Praomys
Meriones
Meriones_crassus
Acomys cahirinus
Lophuromys sikapusi
Mesocricetus_auratus
Oryzomys
Trichys_fasciculata
Heterocephalus_glaber
Coendou_melanurus
Cavia_porcellus
Ctenomys_maulinus
Octodon_degus
Loxodonta_africana
Procavia_capensis
Echinops_telfairi
Micropotamogale
Myotis_lucifugus
Canis_familiaris
Felis_catus
Ovis_aries
Equus_caballus
Tapirus_terrestris
Vicugna
Sus_scrofa
Bos_taurus
Tursiops_truncatus
Erinaceus
Sorex_araneus
Neomys_anomalus
Choloepus_hoffmanni
Dasypus

```
                         601
Monodelphis              GACAGCT--- -------ACT TCCTGG-GCA CCA---AGAC G--------- ----------
Macropus                 GAGAGCC--- -------ACT CCCTGGTATG TCA---AGCG T--------- ----------
Microcebus_murinus       CACGGCA--- -------ACC TCCTGG-TCA CCA---AGCG G--------- ----------
Tarsius_syrichta         CAACACC--- -------GCC CCCTGC-TCA CCG---AGAG G--------- ----------
Callithrix_jacchus       CTAA------ -------A-- -CCTGG-TCA CCA---AGAG T--------- ----------
Macaca_mulatta           CTAA------ -------AGC TCCTGG-TCA CCA---AGAC G--------- ----------
Pongo_pygmaeus           CTAA------ -------AGC TCCTGG-TCA CCA---AGAG G--------- ----------
Gorilla_gorilla          CTAA------ -------AGC TCCTGG-TCA CCA---AGAG G--------- ----------
Homo_sapiens             CTAA------ -------AGC TCCTGG-TCA CCA---AGAG G--------- ----------
Pan_troglodytes          CTAA------ -------AGC TCCTGG-TCA CCA---AGAG G--------- ----------
Ochotona_princeps        CAAAGCC--- -------ACC TCCTGG-TCA CCA---CAAG G--------- ----------
Oryctolagus_cuniculus    CAAAGCA--- -------ACC TGCTGG-TCA CCA---AGAG G--------- ----------
Marmota_monax            CAAAGCA--- -------ACC TCCTGG-TCA ------CAAG G--------- ----------
Aplodantia_rufa          TAAAGCA--- -------ACC TCCTGG-TCA CCA---CAAG G--------- ----------
Spermophilus             CAAAGCA--- -------ACC TCCTGG-TCA ------CAAG G--------- ----------
Anomamorus               CAAAGCA--- -------CCC TCCTGC-TCA CCA---TGAG G--------- ----------
Maxomys                  ---------- ---------- -------TCA CCA---CAGA G--------- ----------
Rattus rattus            ---------- ---------- -------TCA CCA---CAGA G--------- ----------
Rattus exulans           ---------- ---------- -------TCA CCA---CAGA G--------- ----------
Rattus_norvegicus        ---------- ---------- -------TCA CCA---CAGA G--------- ----------
Mus_musculus             ---------- ---------- -------GCA CCA---CAAA G--------- ----------
Mus_spretus              ---------- ---------- -------GCA CCA---CAAA G--------- ----------
Mus_pahari               ---------- ---------- -------TTG CCA---CAAA G--------- ----------
Praomys                  ---------- ---------- -------TCA CCA---CAAA G--------- ----------
Meriones                 ---------- ---------- -------TCA CCA---CGAA G--------- ----------
Meriones_crassus         ---------- ---------- -------TCA CCA---CGAA G--------- ----------
Acomys cahirinus         ---------- ---------- -------TCA CCA---GGGA G--------- ----------
Lophuromys sikapusi      ---------- ---------- -------TCA CCA---CGAA G--------- ----------
Mesocricetus_auratus     ---------- ---------- -------TTA AAA------- ---------- ----------
Oryzomys                 ---------- ---------- -------TTA AAA------- ---------- ----------
Trichys_fasciculata      CAAAACA--- -------ATC TCCTGC-TGG CCA---AGAG G--------- ----------
Heterocephalus_glaber    CAAAACA--- -------ATC TCCTGG-TGA CCA---AGAG G--------- ----------
Coendou_melanurus        CAAAACA--- -------ACC TCCTGG-AAC CCA---AGAG G--------- ----------
Cavia_porcellus          CAAAACA--- -------ACC TCCTGG-TGG CCA---AGAA G--------- ----------
Ctenomys_maulinus        CAAAACA--- -------ACC TCCGGG-TGG CTG---AGAG G--------- ----------
Octodon_degus            CAAAACA--- -------ACC TCCGGG-TGG CTG---AGAG G--------- ----------
Loxodonta_africana       CCAAGCA--- -------GCC CCCTGC-TCA CCA---GGAG G--------- ----------
Procavia_capensis        CCAAGCA--- ---------- GTGTCC-TTA CCA---AGAG G--------- ----------
Echinops_telfairi        CCAAACCCAG AGCAGATACA CTATGC-ACA CCA---GAAT G--------- ----------
Micropotamogale          CCATACA--- -------TTC CTGTGC-CCG CCA---GAGT G--------- ----------
Myotis_lucifugus         CGCAGCC--- -------AGG CCCTGG-GCG CCA---ACAC G--------- ----------
Canis_familiaris         CCAAGCA--- -------AGC CTCTGG-TCA TCA---AGAG G--------- ----------
Felis_catus              CAAAACA--- -------AGC CTCTGG-TCA CCA---CGAG G--------- ----------
Ovis_aries               CAAAGCA--- -------GGC CTCTGG-TA- ---------- ---------- ----------
Equus_caballus           CGAAGCC--- -------AGC GCCCCC-TCA CCA---AGAG G--------- ----------
Tapirus_terrestris       CAGAGCA--- -------AGC CCCTGG-TCA CCA---AGAG G--------- ----------
Vicugna                  CAGATCA--- -------GGC CCCTGA-TCG CCC---AGAG G--------- ----------
Sus_scrofa               CAAAGCA--- -------AGC CCCTGG-TCA CCA---AGA- ---------- ----------
Bos_taurus               CTAAGCA--- -------GGC CTCTGG-TCG CCA---AGAG G--------- ----------
Tursiops_truncatus       CAAAGCA--- -------CGC CCCTGG-TCG CCA---AGAG G--------- ----------
Erinaceus                CAAAGCA--- -------AAC CACTGG-CCA TCA---GGAG GATAACTGCT GCTCCCCGCC
Sorex_araneus            CACAGGC--- -------AAC CCCTGG-TCA CCACCCGCAG G--------- ----------
Neomys_anomalus          C--------- -------AGC CCCTGG-CCA CCC---ACAG G--------- ----------
Choloepus_hoffmanni      CAAAGGA--- -------ACT CCCAGG-TCA CCA---AGAG G--------- ----------
Dasypus                  CAAAGCT--- -------AGC CCCTGG-CCA CCA---AGAG G--------- ----------
```

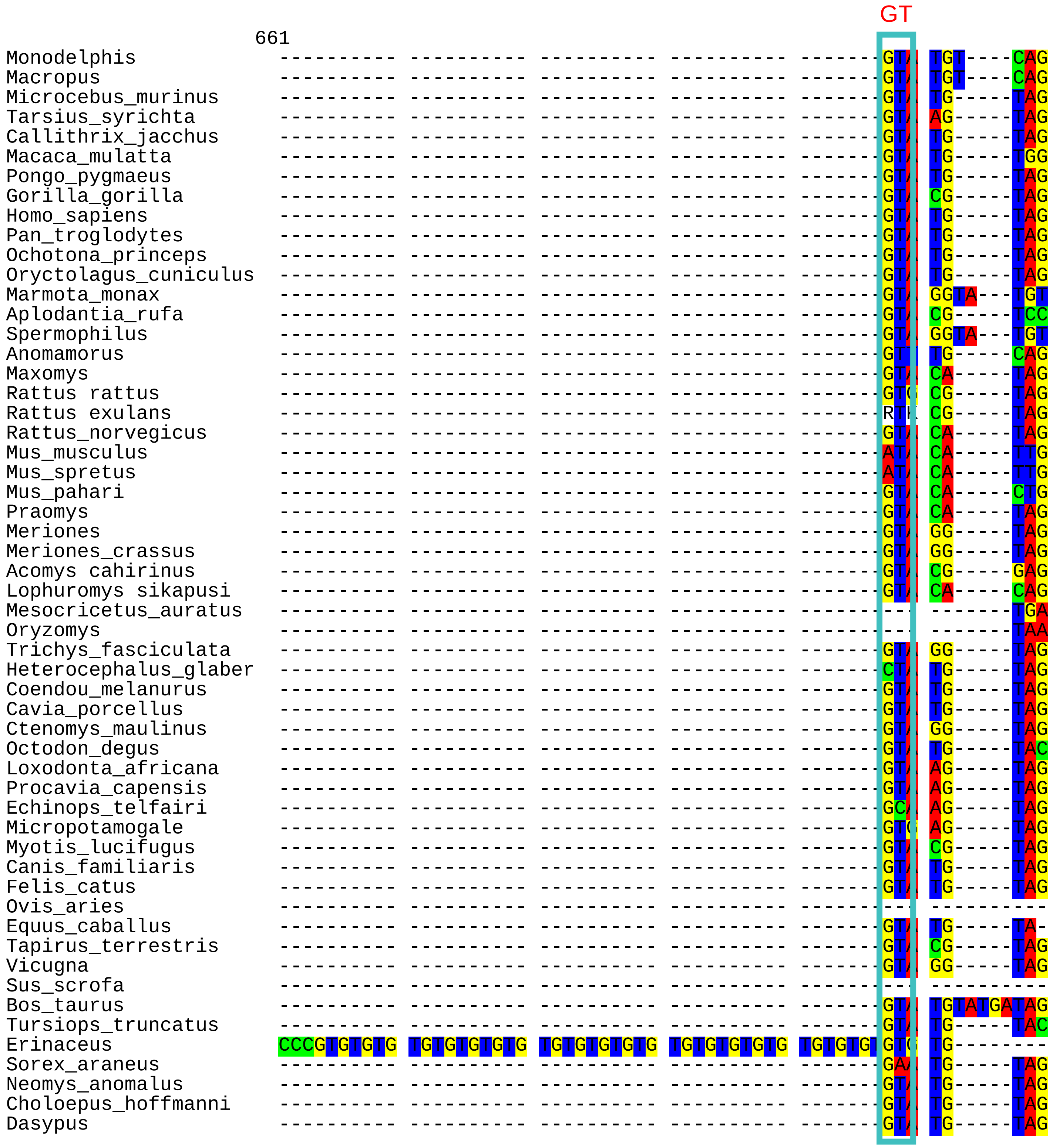
GT
661
Monodelphis
Macropus
Microcebus_murinus
Tarsius_syrichta
Callithrix_jacchus
Macaca_mulatta
Pongo_pygmaeus
Gorilla_gorilla
Homo_sapiens
Pan_troglodytes
Ochotona_princeps
Oryctolagus_cuniculus
Marmota_monax
Aplodantia_rufa
Spermophilus
Anomamorus
Maxomys
Rattus rattus
Rattus exulans
Rattus_norvegicus
Mus_musculus
Mus_spretus
Mus_pahari
Praomys
Meriones
Meriones_crassus
Acomys cahirinus
Lophuromys sikapusi
Mesocricetus_auratus
Oryzomys
Trichys_fasciculata
Heterocephalus_glaber
Coendou_melanurus
Cavia_porcellus
Ctenomys_maulinus
Octodon_degus
Loxodonta_africana
Procavia_capensis
Echinops_telfairi
Micropotamogale
Myotis_lucifugus
Canis_familiaris
Felis_catus
Ovis_aries
Equus_caballus
Tapirus_terrestris
Vicugna
Sus_scrofa
Bos_taurus
Tursiops_truncatus
Erinaceus
Sorex_araneus
Neomys_anomalus
Choloepus_hoffmanni
Dasypus

```
                          721
Monodelphis               TCATTTGCT- ---------- -----GAATA TTTTTGAGTA GTTCAG---- -------ACC
Macropus                  TCATTTGGTA TGTCAGTCAT TTGCTGGTTA TTTTTGAGCT GATCAG---- -------ACC
Microcebus_murinus        ACATTTGC-- ---------- ------AGTT TTCACGCATT GCTCAG---- -------AGC
Tarsius_syrichta          ACATTCGC-- ---------- ------TGTT --CTG-CAGT TTTCAG---- -------AGC
Callithrix_jacchus        ACATTTGT-- ---------- ------TGTT TTCCTACGTT TCTAAG---- -------AGC
Macaca_mulatta            GCATTTGC-- ---------- ------TGTC TTCCTGGATT TCTCAG---- -------AGC
Pongo_pygmaeus            GCATTTGC-- ---------- ------TGTC TTCCTGGATT TCTCAG---- -------AGC
Gorilla_gorilla           GCATTTGC-- ---------- ------TGTC TTCCTGGATT TCTCAG---- -------AGC
Homo_sapiens              GCATTTGC-- ---------- ------TGTC TTCCTGGATT TCTCAG---- -------AGC
Pan_troglodytes           GCATTTGC-- ---------- ------TGTC TTCCTGGATT TCTCAG---- -------AGC
Ochotona_princeps         ACACCTGC-- ---------- -----CACTC TCCTGCCACC TTTCAG---- -------AAC
Oryctolagus_cuniculus     ACACTTGC-- ---------- -----CACTC TCCTGAATCT TT-CAG---- -------GGC
Marmota_monax             TCACCTGC-- ---------- -----TGTT- --CTGCAG-T TTTCAG---- -------AGC
Aplodantia_rufa           ACTTGCTA-- ---------- -----TTCT- GCA-GTTTTT ATCTAG---- --------AG
Spermophilus              TCACCTGC-- ---------- --------AG TTCTGCAGTT TTTCAG---- -------AGC
Anomamorus                GCATTTGC-- ---------- -----CATTT TCCTG-CATT TTTCAG---- -------AGC
Maxomys                   GCACTTGC-- ---------- -----TGTTT CCCTG-AAAT TTTCAGTTTT T-----AATC
Rattus rattus             GCATTTGC-- ---------- -----TGTCT CCCTG-AAAA TTTCAGTTTT T-----AATA
Rattus exulans            GCATTTGC-- ---------- -----TGTCT CCCTG-AAAA TTTCAGTTTT T-----AATC
Rattus_norvegicus         GCATTTGC-- ---------- -----TGTCT CCCTG-AAAA TTTCAGTTTT T-----AATC
Mus_musculus              GCATTTGC-- ---------- ------TGTC TCCCTGAAAT GTTCAGTTTA TTAATTAATC
Mus_spretus               GCATTTGC-- ---------- -----TGTCT CCCTG-AAAT GTTCAGTTTA TTAATTAATC
Mus_pahari                GCATTTGC-- ---------- -----TGGTT CCCTG-AAAT GTTCAGTTGT TTA----ACC
Praomys                   GCATCTGC-- ---------- -----TGTTT CCCTG-AAAT TTTCAGTTTT T-----AATC
Meriones                  GCATTTGC-- ---------- -----TGTTT CCCTGACAAT TTTGAGTTTT -----CAGTC
Meriones_crassus          GCATTTGC-- ---------- -----TGTTT CCCTGACAAT TTTGAGTTTT -----CAGTC
Acomys cahirinus          GC-------- ---------- ---------- ---------- ---------- ----------
Lophuromys sikapusi       GC-------- ---------- ---------- ---------- ---------- ----------
Mesocricetus_auratus      ACTTTTAA-- ---------- ---TTAAACA TTTTTTATTT TTGCAG---- ------TACT
Oryzomys                  ACTTTCAA-- ---------- ---GTTAAGA TTTTT-ATTT TTGCAG---- ------TATT
Trichys_fasciculata       GCATTTGC-- ---------- -----TGTC- CCCCG-CGTT TCTCAG---- -------AGG
Heterocephalus_glaber     ACATTTGC-- ---------- -----TGTT- GCCTG--ATT TCTCAG---- -------GGC
Coendou_melanurus         ACATTTGT-- ---------- -----TGTT- GCTTC-CGTT TGTCAG---- -------AGC
Cavia_porcellus           ACTTTTG--- ---------- ----TTGCT- GCCTG-CGTT TCTCAG---- -------ACC
Ctenomys_maulinus         ACCTTTGC-- ---------- ------CTTT GCCTGCATTT TTTCAG---- -------AGC
Octodon_degus             ACCTTTGC-- ---------- ------CTTT GCCTGCA-TT TTTCAG---- -------TGC
Loxodonta_africana        TCATTTGCT- ---------- ---AAGTGTT TCCATGCGTT TTCCAG---- -------AGC
Procavia_capensis         TCATTTGCT- ---------- ---AAGTGTT CCCATGTG-- ---------- ----------
Echinops_telfairi         TCATTTGCT- ---------- ---CGGTGTT TTCATACATT TGTCAG---- -------AAC
Micropotamogale           TCATTCTCT- ---------- ---TAGTGTT TTCACACATT TTCCAG---- -------AAC
Myotis_lucifugus          ACCTTCGA-- ---------- ------TGCT TTCCGGCAAT GCTCAG---- -------AGC
Canis_familiaris          ACATTTGC-- ---------- ------TGCT TTTCTGCATT TTTCAA---- -------AAC
Felis_catus               GTGTTTGC-- ---------- ------TGCT TTTCTGCGTT TCTCAA---- -------AGC
Ovis_aries                ---------- ---------- ---------- ---------- ---------- ----------
Equus_caballus            ACATTCGC-- ---------- ------TGCT TTCCCGCATT TCTCAG---- -------AGC
Tapirus_terrestris        ACATTTGC-- ---------- ------TGCT TTCCCGCATC TTTCAG---- -------AGC
Vicugna                   ACTTTGGC-- ---------- ------TGCT TTCCTGCATT TTTCAC---- -------AGC
Sus_scrofa                ----CTAC-- ---------- ------TACT TTCCTGCATT TTTCAG---- -------ACC
Bos_taurus                ATACTTTC-- ---------- ------TGCT TTCCCACATT TTT-AG---- -------AGC
Tursiops_truncatus        ACACTTGC-- ---------- ------TGCT TTCCTGCATT TTTCGG---- -------AGC
Erinaceus                 ---------- ---------- ---------- ---TTTCGTC TCTCTG---- -------AAC
Sorex_araneus             AGCCTTGC-- ---------- ------CTCT TTCTGCATTT TCCCAG---- -------GAC
Neomys_anomalus           ACACTTCC-- ---------- ---------- ---TGCCTTT TCCCAG---- -------GAC
Choloepus_hoffmanni       TCATTTGCT- ---------- ---AAGTATT TTCATACATT TTTCAG---- -------AAC
Dasypus                   TCACTTGCT- ---------- ---AAGTGTG TTCATACATT TTTCAG---- -------ACT
```

```
                          781
Monodelphis               AGGCAGG--- ---------- -----CCT-T TGGTC--AAT GCCTGGCACA ----------
Macropus                  AGGTGA---- ---------- ---------- TATGGTCAAT GCCTAGAACA TGGTTAAGTG
Microcebus_murinus        TGAGTTGGTT TTTTTTTTTT TTTTTTTT-T GAGAC--AGA CTCTCA---- ----------
Tarsius_syrichta          TGAG------ ---------- -----TTT-T TGGCC--AGG GGAT------ ----------
Callithrix_jacchus        TGGG------ ---------- -----TTT-T TAGCC--AGA GGTT------ ----------
Macaca_mulatta            TGAG------ ---------- -----TTT-T TAGCC--AGA GGTT------ ----------
Pongo_pygmaeus            TGAG------ ---------- -----TTT-T TAGCC--AGA GGTT------ ----------
Gorilla_gorilla           TGAG------ ---------- -----TTT-T TAGCC--AGA GGTG------ ----------
Homo_sapiens              TGAG------ ---------- -----TTT-T TAGCC--AGA GGTT------ ----------
Pan_troglodytes           TGAG------ ---------- -----TTT-T TAGCC--AGA GGTT------ ----------
Ochotona_princeps         TGAG------ ---------- -----TGT-T TAGCT--AGA GG-------- ----------
Oryctolagus_cuniculus     TCAG------ ---------- -----TGT-T CAGCT--AGA GGTT------ ----------
Marmota_monax             TAGG------ ---------- -----GTT-T TACAC--AGA G-TC------ ----------
Aplodantia_rufa           TGGC------ ---------- ---------- ---------- ---------- ----------
Spermophilus              TAAG------ ---------- -----GTT-T TAGAC--AGA G-TCG----- ----------
Anomamorus                ---------- ---------- -------T-T TTGCC--AGA T-TT------ ----------
Maxomys                   TGAATT-GGG CAATTTGCTA ATGAAATTGT CCAAT--AAA TACTGAAATA CTCTCTTTTC
Rattus rattus             TGAATT-GAG TAATTTGTTA ATGAAATTGT CCAGT--AAA TACTCAAATA CTCTCTTTTC
Rattus exulans            TGAATT-GAG TAATTTGTTA ATGAAATTGT CCAGT--AAA TACTCAAATA CTCTCTTTTC
Rattus_norvegicus         TGAATT-GAG TAATTTGTTA ATGAAATTGT CCAGT--AAA TACTCAAATA CTCTCTTTTC
Mus_musculus              TGAATT-GGG TAATTTGTTA ATGAAATTGT CCAGC--AAA TACTGAAATA ATCTTTCTTT
Mus_spretus               TGAATT-GGG TAATTTGTTA ATGAAATTGT CCAGC--AAA TACTGAAATA ATCTTTCTTT
Mus_pahari                TGAATT-GGC TAATTTGTTA ATGAAATTGT CCAGT--AAA TACTGAAATA TTCTTTCTTT
Praomys                   TGAATT-GGG TAATTTGTTA ATGAAATTGT CCAGT--AAA TATTAAAATA CTCTTTCTTT
Meriones                  TGGATTTGGG TAATTTGTTG ATAAAAT--T GTGCGGTAAA TACTGTCTTT T--CTTTTCT
Meriones_crassus          TGGATTTGGG TAATTTGTTG GTAAAAT--T GTGCGGTAAA TACTGTCTTT TTTCTTTTYT
Acomys cahirinus          ---------- ---------- ---------- ---------- ---------- ----------
Lophuromys sikapusi       ---------- ---------- ---------- ---------- ---------- ----------
Mesocricetus_auratus      AGGC------ ---------- ---------- ---------- ---------- ----------
Oryzomys                  AGGT------ ---------- ---------- ---------- ---------- ----------
Trichys_fasciculata       TGG------- ---------- -----GTT-T TTGCC--AG- --TT------ ----------
Heterocephalus_glaber     TGA------- ---------- -----CTT-T TAGCC--AG- --TT------ ----------
Coendou_melanurus         TGA------- ---------- -----CTT-T TAGCT----- ---------- ----------
Cavia_porcellus           TGA------- ---------- -----TTT-T AGGCG----- ---------- ----------
Ctenomys_maulinus         TGA------- ---------- -----TTT-G TGGCC--AGT TACTTGTGTC AG--------
Octodon_degus             TGA------- ---------- -----CTT-G TGGCC--AGG TACTAGTGTC AG--------
Loxodonta_africana        CGAG------ ---------- -----TTT-T TAGCC--AGA GGT------- ----------
Procavia_capensis         ---------- ---------- -----TTT-T TAGCC--AGA GCT------- ----------
Echinops_telfairi         TGAGTT---- ---------- -----TTT-T GGGTC--AGA GTG------- ----------
Micropotamogale           TGAT------ ---------- -----ATT-T TAACA-ATGA CAT------- ----------
Myotis_lucifugus          TGAG------ ---------- -----GCT-T CAGCT--AGC GGTG------ ----------
Canis_familiaris          TGAG------ ---------- -----TTT-T TAGCT--GGA GGCT------ ----------
Felis_catus               TGAG------ ---------- -----CTT-T GGGCT--GGA GGCG------ ----------
Ovis_aries                ---------- ---------- ---------- ---------- ---------- ----------
Equus_caballus            TGAG------ ---------- -----CTT-T TAGCC--AGA GG-T------ ----------
Tapirus_terrestris        TGAG------ ---------- -----TTT-T TAGCC--AGA AGCT------ ----------
Vicugna                   TGGC------ ---------- -----TTT-T CAGCC--AGA GGCT------ ----------
Sus_scrofa                TGAG------ ---------- -----TTT-T TAGCC--AGA GACT------ ----------
Bos_taurus                TGG------- ---------- -----GTT-T TAGCT--AGA TGCT------ ----------
Tursiops_truncatus        TGAG------ ---------- -----TTT-T TAGCC--AGA GGCT------ ----------
Erinaceus                 TGAC------ ---------- -----ATT-T TC-------- ---------- ----------
Sorex_araneus             AGTG------ ---------- -----CTT-T AACGC--AGA GGCT------ ----------
Neomys_anomalus           AGTG------ ---------- -----CTG-T TAGCC--AGA GGCT------ ----------
Choloepus_hoffmanni       TGAA------ ---------- -----GTT-T TAGCC--AGT TGCT------ ----------
Dasypus                   AAAG------ ---------- -----TTC-T TAGCC--AGA GCT------- ----------
```

```
                          841
Monodelphis               -TTAAT---A AATGTTTGTT GAATGATTAA T--------- -----AATA- ----------
Macropus                  TTTAAT---A AATGCTTATG AGGTGATTGA T--------- ------ATT- ----------
Microcebus_murinus        ---------- ---CTTTGTT GCCC------ ---------- ------AGG- ----------
Tarsius_syrichta          ---------- ---ATTTAT- --TT------ ---------- ------ATG- ----------
Callithrix_jacchus        ---------- ---GCTTAT- --TT------ ---------- ------ATG- ----------
Macaca_mulatta            ---------- ---GCTTAT- --TT------ ---------- ------ATG- ----------
Pongo_pygmaeus            ---------- ---GCTTAT- --TT------ ---------- ------ATG- ----------
Gorilla_gorilla           ---------- ---GTTTAT- --TT------ ---------- ------ATG- ----------
Homo_sapiens              ---------- ---GCTTAT- --TT------ ---------- ------ACG- ----------
Pan_troglodytes           ---------- ---GCTTAT- --TT------ ---------- ------ACG- ----------
Ochotona_princeps         ---------- -----TTAT- --TT------ ---------- ------ATG- ----------
Oryctolagus_cuniculus     ---------- ---ACTTCC- --GT------ ---------- ------ACG- ----------
Marmota_monax             ---------- ---ACTTAT- CTGTG----- ---------- ---------- ----------
Aplodantia_rufa           ---------- -----TTAT- CT-------- ---------- ------ATG- ----------
Spermophilus              ---------- ----CTTTCT CTGTG----- ---------- ---------- ----------
Anomamorus                ---------- ---GCTTAT- --TT------ ---------- ------ACA- ----------
Maxomys                   TTTTCTTTTC TTTTTTCTTT TCTTTATCCC TCTTCCTCTT CCCCCTCCTA CTCTTTAATA
Rattus rattus             TTTCTC---- TTCCTCTTCT TTCTT----- ---------- -------CTA CTCTTTAATA
Rattus exulans            TTTATTTCTC TTCCTCTTCT TTCTT----- ---------- -------CTA CTCTTTAATA
Rattus_norvegicus         TTTATTTCTC TTCCTCTTCT TTCTT----- ---------- -------CTA CTCTTTAATA
Mus_musculus              TCTT------ --------AC TTCCTCCTCC TCCCCCC--- -TTCCTCCTC TTTTTTAATA
Mus_spretus               TCTT------ --------AC TTCCTCCTCC TCCCCCC--- -TTCCTCCTC TTTTTTAATA
Mus_pahari                T--------- -----CTTTC TTTCTCCTCC TCCCCCCCCT CTTCCTCCTC GTCTTTAATA
Praomys                   T--------- ----CTTTTT TTCTTTCTCC TCTTTTCCTT CTCCCTCCTC TTCTTTAATA
Meriones                  TT---TCTTT CTTTTTTTTT TTTTT----- ---------- --------AA AGATAAAATT
Meriones_crassus          TTYTTTYTTT YTTTTTTTTT TTTTT----- ---------- ---------A AGATAAAAGT
Acomys cahirinus          ---------- ---------- ---------- ---------- ---------- ----------
Lophuromys sikapusi       ---------- ---------- ---------- ---------- ---------- ----------
Mesocricetus_auratus      ---------- ---------- ---------- ---------- ---------- -TTTTAAACC
Oryzomys                  ---------- ---------- ---------- ---------- ---------- -TTTAAACCC
Trichys_fasciculata       ---------- ---AC-TGT- --GT------ ---------- ------ATG- ----------
Heterocephalus_glaber     ---------- ---ACTTAT- --GT------ ---------- ------ATG- ----------
Coendou_melanurus         ---------- ----CTTGT- --AT------ ---------- ------AAG- ----------
Cavia_porcellus           ---------- ---ATTACTT GTGT------ ---------- ------CAG- ----------
Ctenomys_maulinus         ----CTAACG TGCACTTGCT TTGT------ ---------- ------AAG- ----------
Octodon_degus             ----CTAACG TGTACTTGC- --GT------ ---------- ------AAG- ----------
Loxodonta_africana        ---------- ---ACTCTT- --GA------ ---------- ------ATG- ----------
Procavia_capensis         ---------- ---GCTCTT- --GT------ ---------- ------AGG- ----------
Echinops_telfairi         ---------- ---ACTCCT- --GA------ ---------- ------ATG- ----------
Micropotamogale           ---------- ---GCTGTT- --GA------ ---------- ------ATG- ----------
Myotis_lucifugus          ---------- ---GCTCCT- --TT------ ---------- ------CCA- ----------
Canis_familiaris          ---------- ---GCTCCT- --TT------ ---------- ------ATG- ----------
Felis_catus               ---------- ---GCTCCT- --TT------ ---------- ------A--- ----------
Ovis_aries                ---------- ---------- ---------- ---------- ---------- ----------
Equus_caballus            ---------- ---GTTCCT- --TT------ ---------- ------ATC- ----------
Tapirus_terrestris        ---------- ---GCTCCT- --TT------ ---------- ------ATG- ----------
Vicugna                   ---------- ---TCTCCT- --AT------ ---------- ------ATG- ----------
Sus_scrofa                ---------- ---GCTTCT- --TT------ ---------- ------GCG- ----------
Bos_taurus                ---------- ---ACTCCT- --TT------ ---------- ------CTG- ----------
Tursiops_truncatus        ---------- ---GCTCCT- --TC------ ---------- ------ACG- ----------
Erinaceus                 ---------- ---GCTTCT- --TT------ ---------- ------ATT- ----------
Sorex_araneus             ---------- ---GATCCT- ---------- ---------- -------TT- ----------
Neomys_anomalus           ---------- ---GATTCT- ---------- ---------- ------CTG- ----------
Choloepus_hoffmanni       ---------- ---CCTTTA- --GT------ ---------- ------ATG- ----------
Dasypus                   ---------- ---GCTCCTG TTTC------ ---------- ------ATG- ----------
```

```
                         901
Monodelphis              ---------- -AATATGTAC TTAGTTGGTG ACATT----- ---------- ----------
Macropus                 ---------- -AATATGTAC TTAGTCTGTG ACATT----- ---------- ----------
Microcebus_murinus       ---------- -CTAGACTG- --AGT----G CCGTG----- ---------- ----------
Tarsius_syrichta         ---------- -ATAATTTCC T-AAT----- ACAGT----- ---------- ----------
Callithrix_jacchus       ---------- -ATAATTTTC T-GAT----- ATATT----- ---------- ----------
Macaca_mulatta           ---------- -ATAATTTTC G-GAT----- ATATT----- ---------- ----------
Pongo_pygmaeus           ---------- -ATAATTCTT G-GAT----- ATATT----- ---------- ----------
Gorilla_gorilla          ---------- -ATAATTCTT G-GAT----- ATATT----- ---------- ----------
Homo_sapiens             ---------- -ATAATTCTT G-GAT----- ATATT----- ---------- ----------
Pan_troglodytes          ---------- -ATAATTCTT G-GAT----- ATATT----- ---------- ----------
Ochotona_princeps        ---------- -GTAACTGGT T-GAT----G ATATT----- ---------- ----------
Oryctolagus_cuniculus    ---------- -GTAACTGAC T-GAT----A ATATT----- ---------- ----------
Marmota_monax            ---------- -ATAATTTGT C-TAT----G ACATT----- ---------- ----------
Aplodantia_rufa          ---------- -ACAATTTGC T-GAT----G ACATT----- ---------- ----------
Spermophilus             ---------- -TTAATTTGC C-TAT----G ACATT----- ---------- ----------
Anomamorus               ---------- -ATCATTTGC T-GAT----G ACATT----- ---------- ----------
Maxomys                  TAGCATCATG CCATAAAGTT CTTGGT---C TCAAACTCCA GACTATCCTC CTGCCTCAAT
Rattus rattus            TAGCATCATG CCATAAAGTT CTTGGT---C TCAAACTCCA GACTATCCTC CTGCCTCAGT
Rattus exulans           TAGCATCATG CCATAAAGTT CTTGGT---C TCAAACTCCA GACTATCCTC CTGCCTCAGT
Rattus_norvegicus        TAGCATCATG CCATAAAGTT CTTGGT---C TCAAACTCCA GACTATCCTC CTGCCTCAGT
Mus_musculus             AAGCCCCACA CCATAAAGTT CTTGGTGGTC TCAAACTCCA GACTCCCCTC CTGCCTCAGT
Mus_spretus              AAGCCCCACA CCATAAAGTT CTTGGTGGTC TCAAACTCCA GACTCCCCTC CTGCCTCAGT
Mus_pahari               AAGCCTCACA CCATAAAGTT CTTGGTGGTC TCAAACTCCA GACTACCCTC CTGCCTCAGT
Praomys                  TAGCATCACA CCATAAAGTT CTTGGTGC-C TCAAACTCCA GACTACCATC CTGCCTCCAT
Meriones                 TATCACACCA TAAATTTCAG GCTGGTCTCA TTCTCCTGCC TCAGTCTCTT ATGTGCTGGG
Meriones_crassus         TATCACACTA TAAATTCCAG CCTGGTCTCA TT-TCCTGCC TCAGTCTCTT GTGTGCTGGG
Acomys cahirinus         ---------- ---------- ---------- ---------- ---------- ----------
Lophuromys sikapusi      ---------- ---------- ---------- ---------- ---------- ----------
Mesocricetus_auratus     CAGGGCCTAT CCATGTCAG- ---------- ---------- ---------- ----------
Oryzomys                 TAGAGCCTAT ACATGCCAA- ---------- ---------- ---------- ----------
Trichys_fasciculata      ---------- -ACAAGGTGC A-GAT----G GCATT----- ---------- ----------
Heterocephalus_glaber    ---------- -ATAATGTGC T-GAT----G GCACT----- ---------- ----------
Coendou_melanurus        ---------- -ATAACGCGC T-GAT----G ACATT----- ---------- ----------
Cavia_porcellus          ---------- -ATAATATGC T-GAT----G ACATT----- ---------- ----------
Ctenomys_maulinus        ---------- -CTAACGTGC T-GAT----G ACTTT----- ---------- ----------
Octodon_degus            ---------- -CTAACATGC T-GAC----G ACTTC----- ---------- ----------
Loxodonta_africana       ---------- -ATA--CGGC T-GC-ATTGT CCGCT----- ---------- ----------
Procavia_capensis        ---------- -ATACACCAC T-GCTATTAT ACACT----- ---------- ----------
Echinops_telfairi        ---------- -GTATATTGC T-GCTACTAA GTACT----- ---------- ----------
Micropotamogale          ---------- -GCAGATTGT T-GCGACTAA AAGAT----- ---------- ----------
Myotis_lucifugus         ---------- -ATGACTTAC TTGTT----A ATATT----- ---------- ----------
Canis_familiaris         ---------- -ACAAACTGC T-GAT----- --ACC----- ---------- ----------
Felis_catus              ---------- -ATGATCAGT T-GCT----G ACACT----- ---------- ----------
Ovis_aries               ---------- ---------- ---------- ---------- ---------- ----------
Equus_caballus           ---------- -ATAAATTGC ---------- ---------- ---------- ----------
Tapirus_terrestris       ---------- -ATAAATTGC T-GAT----- ATTGT----- ---------- ----------
Vicugna                  ---------- -ATAAATTG- ---------- ---------- ---------- ----------
Sus_scrofa               ---------- -ATAAATTG- ---------- ---------- ---------- ----------
Bos_taurus               ---------- -ATAAATTG- ---------- ---------- ---------- ----------
Tursiops_truncatus       ---------- -ATAAACTG- ---------- ---------- ---------- ----------
Erinaceus                ---------- -ATAAAGTGC T-AAT----- --ACT----- ---------- ----------
Sorex_araneus            ---------- -GTAACTTGC T-GCTA---- ACAGT----- ---------- ----------
Neomys_anomalus          ---------- -CAAGC-TGC T-GCT----G AGTGC----- ---------- ----------
Choloepus_hoffmanni      ---------- -ATAAACTAC A-GAT----- --TTT----- ---------- ----------
Dasypus                  ---------- -ACACATTGC G-GGT----- --GTT----- ---------- ----------
```

```
                         961
Monodelphis              ---------- ---------- ---------- ---------- ----AACAAT ATCATACACT
Macropus                 ---------- ---------- ---------- ---------- ----AACAAT ATCATGCGCT
Microcebus_murinus       ---------- ---------- ---------- ---------- -------GCG TCAGCCTG--
Tarsius_syrichta         ---------- ---------- ---------- ---------- -------GTG C---------
Callithrix_jacchus       ---------- ---------- ---------- ---------- -------GGA C---------
Macaca_mulatta           ---------- ---------- ---------- ---------- -------ATA C---------
Pongo_pygmaeus           ---------- ---------- ---------- ---------- -------ATA C---------
Gorilla_gorilla          ---------- ---------- ---------- ---------- -------ATA C---------
Homo_sapiens             ---------- ---------- ---------- ---------- -------ATA C---------
Pan_troglodytes          ---------- ---------- ---------- ---------- -------ATA C---------
Ochotona_princeps        ---------- ---------- ---------- ---------- -------TGA C---------
Oryctolagus_cuniculus    ---------- ---------- ---------- ---------- -------GTA C---------
Marmota_monax            ---------- ---------- ---------- ---------- -------GTA C---------
Aplodantia_rufa          ---------- ---------- ---------- ---------- -------GTA C---------
Spermophilus             ---------- ---------- ---------- ---------- -------GTA A---------
Anomamorus               ---------- ---------- ---------- ---------- -------GTA C---------
Maxomys                  CTCTTGTGTT GTGCTGGGAC TTATACAT-- ---------- ---------- ----GCCAGG
Rattus rattus            CTCTGGTGT- ---------- ---------- --------GC TGGGGCTTAT ACATGCCAGG
Rattus exulans           CTCTGGTGTG ---------- ---------- ---------C TGGGSCTTAT ACATGCCAGG
Rattus_norvegicus        CTTTGGTGT- ---------- ---------- --------GC TGGGGCTTAT ACATGCTAGG
Mus_musculus             CTCT--TGT- GTGCTGGGAT TACAACGTAT ATCATCAGGC TGGGGCTTAT GCATGCCAGG
Mus_spretus              CTCT--TGT- GTGCTGGGAT TACAACGTAT ATCATCAGGC TGGGGCTTAT GCATGCCAGG
Mus_pahari               CTCT--TGT- GTGCTAGGAT TACAACCTAT GTCATCAGGC TGGGGCTTAT ACATGCCAGG
Praomys                  CTCTCTTGT- GTGCTGAGAT TACAACATAT GTCATCAGGC TGGGGTTTAT ACATGCCAAC
Meriones                 ATTACCGGCG CATGCCATCA GGCTGGAGCC CATGCATGCC AGGGGCT--- ----------
Meriones_crassus         ATTACCGGCG CATGCCATCA GGCTGGAGCC CATGAATGCC AGGGGCT--- ----------
Acomys cahirinus         ---------- ---------- ---------- ---------- ---------- ----------
Lophuromys sikapusi      ---------- ---------- ---------- ---------- ---------- ----------
Mesocricetus_auratus     ---------- ---------- ---------- ---------- ---------- ----------
Oryzomys                 ---------- ---------- ---------- ---------- ---------- ----------
Trichys_fasciculata      ---------- ---------- ---------- ---------- -------GTA C---------
Heterocephalus_glaber    ---------- ---------- ---------- ---------- -------GTA T---------
Coendou_melanurus        ---------- ---------- ---------- ---------- -------GTA C---------
Cavia_porcellus          ---------- ---------- ---------- ---------- ---------- ----------
Ctenomys_maulinus        ---------- ---------- ---------- ---------- -------GCA C---------
Octodon_degus            ---------- ---------- ---------- ---------- -------RCA C---------
Loxodonta_africana       ---------- ---------- ---------- ---------- ---------- ----------
Procavia_capensis        ---------- ---------- ---------- ---------- ---------- ----------
Echinops_telfairi        ---------- ---------- ---------- ---------- -------GTA CACAAG----
Micropotamogale          ---------- ---------- ---------- ---------- ---------- ----------
Myotis_lucifugus         ---------- ---------- ---------- ---------- -------GTG C---------
Canis_familiaris         ---------- ---------- ---------- ---------- -------GTG C---------
Felis_catus              ---------- ---------- ---------- ---------- -------GCA T---------
Ovis_aries               ---------- ---------- ---------- ---------- ---------- ----------
Equus_caballus           ---------- ---------- ---------- ---------- ---------- ----------
Tapirus_terrestris       ---------- ---------- ---------- ---------- ---------- ----------
Vicugna                  ---------- ---------- ---------- ---------- ---------- ----------
Sus_scrofa               ---------- ---------- ---------- ---------- ---------- ----------
Bos_taurus               ---------- ---------- ---------- ---------- ---------- ----------
Tursiops_truncatus       ---------- ---------- ---------- ---------- ---------- ----------
Erinaceus                ---------- ---------- ---------- ---------- -------GCG T---------
Sorex_araneus            ---------- ---------- ---------- ---------- -------GCT NNN
Neomys_anomalus          ---------- ---------- ---------- ---------- -------GCT T---------
Choloepus_hoffmanni      ---------- ---------- ---------- ---------- -------GTA C---------
Dasypus                  ---------- ---------- ---------- ---------- -------GTA C---------
```

```
                              1021
Monodelphis                   GCTAGAAAAA TGGTACAAAT CCCATTGTTA GACAGTGTTC ATCCT----- -TTGTAGACA
Macropus                      GCTACAAAAA TGGTACAAAT CCCATTTTTA GGCAGTGGGC ATCCT----- -TTGTAAACA
Microcebus_murinus            GC-------- -----TCACA GCAAC---CT CAATCTCCTG ---------- GGCTCAAGCG
Tarsius_syrichta              GC-------- -----TAAAT AC-------- -GCTTCTCTC ---------- TTCCAAACCA
Callithrix_jacchus            AC-------- -----TAAAT AC-------- -TATTATCTC ---------- TTTCCACCCA
Macaca_mulatta                AC-------- -----TAAAT AATGT---G- TTATTATCTT ---------- TTTCGAGCCA
Pongo_pygmaeus                AC-------- -----TAAAT ACT------A TTATTATCTT ---------- TTTCCACCCG
Gorilla_gorilla               AC-------- -----TAAAT ACT------A TTATTATCTT ---------- TTTCGACCCG
Homo_sapiens                  AC-------- -----TAAAT ACT------A TTATTATCTT ---------- TTTCGACCCG
Pan_troglodytes               AC-------- -----TAAAT ACT------A TTATTATCTT ---------- TTTCGACCCG
Ochotona_princeps             TC-------- -----TAACT ACT------A TTCTTACCTC ---------- TTTCAAACCA
Oryctolagus_cuniculus         AC-------- -----TAACT ACT------A TTCTTAACTC ---------- TTTCAAACCA
Marmota_monax                 AC-------- -----TAAAA AC-------- -TCTTATCGC ---------- TTTTAAACCA
Aplodantia_rufa               AC-------- -----TAAAA ACTGT----- ----TATCGC ---------- TTTTAAACCA
Spermophilus                  AC-------- -----TAAAA AA-------- CTCTTTACCG C--------T TTTTAAACCA
Anomamorus                    AC-------- -----TAAAT AC-------- ---------- ----TATCTC TTTTAGA-CT
Maxomys                       CACTCTA--- ---------C TACSGACCCT ACATCCCTGG CTGTCATCTC CTTTAAACTC
Rattus rattus                 C--------- ---------- -ACTCTAC-T ACGGACCTGG CTGTTATCTC GTTTAAACTC
Rattus exulans                C--------- ---------- -ACTCTAC-T ACGGACCTGG CTGTTATCTC TTTTAAACTT
Rattus_norvegicus             C--------- ---------- -ACTCTAC-T ACAGACCTGG CTGTTATCTC TTTTAAACTC
Mus_musculus                  CACTCTA--- ---------C TACTGACC-T ACATCCTTGG CTGGCATCTC CTTTAAACTC
Mus_spretus                   CACTCTA--- ---------C TACTGACC-T ACATCCTTGG CTGGCATCTC CTTTAAACTC
Mus_pahari                    CACTCTA--- ---------C TACTGACC-T ACATCCCTGC CTGGCATCTC TTTTAAACTC
Praomys                       CACTCTA--- ---------C TACTGRCC-T AGATCCCTGG CTGTCATCTC CTTTAAACTC
Meriones                      ---------- ------CCGT CACTGGCC-G ACATCCCTGG CTGCCGTCTC CTTTAAACGC
Meriones_crassus              ---------- ------CCAT CACTGGCC-G ACATCCCTGG CTGCCGTCTC CTTTAAACGC
Acomys cahirinus              ---------- ---------- ---------- ---------- ---------- ----------
Lophuromys sikapusi           ---------- ---------- ---------- ---------- ---------- ----------
Mesocricetus_auratus          -------GCA AGCAACCTTC TACTGAAC-T ATCTCCCTGA CTGCTGGCTG CTTTAAACTT
Oryzomys                      -------ACA AGCACTCTAC TACTGAAC-T ACATCCCGGG CTGCTGYCTC CTTTAAGCTC
Trichys_fasciculata           AC-------- -----TAAAT ATTGT---TA CTGTTCTCCG ---------- TCTTAAA---
Heterocephalus_glaber         AC-------- -----TAAAT ACTGT---T- TTGTTAGCTC ---------- --TTAAA---
Coendou_melanurus             AC-------- -----CAAAT ACTGT---TA TTGTTATTGC ---------- TCTGAAA---
Cavia_porcellus               ---------- ------GAAC ACTGT---TA TTGTTTTTGC ---------- -TCTTAAA--
Ctenomys_maulinus             AC-------- -----TAAAT ACTGT---TA TTGTTACTGC ---------- TCTTAAA---
Octodon_degus                 AC-------- -----TAAAT ACCGT---TA TTGTTACTGC ---------- TCTTAAG---
Loxodonta_africana            ---------- --------AA GATGTC---- ---------- --------TC TTTCAGAC-T
Procavia_capensis             ---------- ------CAAA GATGCC---- ---------- -AGGAGTTTC TTTCAGAC-T
Echinops_telfairi             ------TGGA GACCGGTCAA GATGGC---- ---------- ---------- -TACAGG-TA
Micropotamogale               ---------- -----ACGGA GCTAGC---- ---------- ---------- TGTGACATGT
Myotis_lucifugus              AC-------- -----TAAAA GCCAT----T ACAAGACCTC ---------- TTTCAAACCA
Canis_familiaris              AC-------- -----TAAAG GCTAT----T ATACTAACTT AC-AAACCAA TTTCAAACCA
Felis_catus                   AC-------- -----TAACA GCGAT----T ATGTTGTCTC ---------- TTACATAC--
Ovis_aries                    ---------- ---------- ---------- ---------- ---------- ----------
Equus_caballus                ---------- ---------- ---------- ---------- ---------- TTTCAAAC--
Tapirus_terrestris            ACAY------ -----TAAAA GCCAT----T ACATTTTCTC ---------- TTTCAAGCCA
Vicugna                       AC-------- -----TAAGA GCTAT----T ACATTATCTA ---------- TTTCGAA---
Sus_scrofa                    AC-------- -----TAAAA AC-------- -TACTATCTC ---------- TTCCAAACCA
Bos_taurus                    AC-------- -----TAAAA GCTAT----- TAGTTATCTC ---------- TTTCCAGTTA
Tursiops_truncatus            AC-------- -----TAAAA GC-------T ATGTTATCTC ---------- TTTCAAATCA
Erinaceus                     GC-------- -----AAAAA GCTAT----T ATATGGTCTC ---------- TTTCAAACCA
Sorex_araneus
Neomys_anomalus               ---------- ---------- ---------- ---------- ---------- TCTCTCACCA
Choloepus_hoffmanni           AA-------- -----CAAAA AGATA---TT AAATTTTCTC ---------- TTTCAAATCA
Dasypus                       AC-------- -----CAAGG GATAG---G- AAGTTGTCGC ---------- TTTCGAATCC
```

```
                         1081
Monodelphis              ATTTCTTGTA GTAGTGGGCA AGAAATTACT TGCTTGTAAT CAGTTTCATC CTTCTC-TTC
Macropus                 ATTTCTTGTA GAAAAGGGCA AGAAATTAC- --CCAATAAT CTATTTCATC CTTGTC-TTC
Microcebus_murinus       A-TCCTC--- ---------- ---------- ---------- -------CTG CCTCAGCCTC
Tarsius_syrichta         G-TTTCT--- ---------- ---------- ---------- -------ATC TTTCTG-ATC
Callithrix_jacchus       ACTTTT---- ---------- ---------- ---------- -------ATC TTTCCG-TTC
Macaca_mulatta           ACTTTT---- ---------- ---------- ---------- -------ATC TTTCTG-TTC
Pongo_pygmaeus           ACTTTT---- ---------- ---------- ---------- -------ATC TTTCTG-TTC
Gorilla_gorilla          ACTAAN---- ---------- ---------- ---------- -------NN- ----------
Homo_sapiens             ACTTTT---- ---------- ---------- ---------- -------ATC TTTCTG-TTC
Pan_troglodytes          ACTTTT---- ---------- ---------- ---------- -------ATC TTTCTG-TTC
Ochotona_princeps        G-TTTTT--- ---------- ---------- ---------- -------GTG GTTGGG-TTC
Oryctolagus_cuniculus    A-TTTTT--- ---------- ---------- ---------- -------GTG ACTGAG-TTC
Marmota_monax            A-TTTTT--- ---------- ---------- ---------- -------GTG ATTCTG-ATC
Aplodantia_rufa          G-TTTTT--- ---------- ---------- ---------- -------GTG GTTTT-----
Spermophilus             ACTTTT---- ---------- ---------- ---------- -------GTG ATTCTG-ATC
Anomamorus               ACTTTTT--- ---------- ---------- ---------- -------ATA GTTTTG-TTC
Maxomys                  CTTTTTT--- ---------- ---------- ---------- -------ATA GTTCTA-TGC
Rattus rattus            CGGTTTT--- ---------- ---------- ---------- -------ACA GTTCTA-TGC
Rattus exulans           CGGTTTT--- ---------- ---------- ---------- -------ACA GTTCTA-TGC
Rattus_norvegicus        CGGTTTT--- ---------- ---------- ---------- -------ACA GTTCTA-TGC
Mus_musculus             CTTTTTTAAA AAGAAA---- ---------- ---------- -------ATA GTTCTA-TGC
Mus_spretus              CTTTTTTAAA AAGAAA---- ---------- ---------- -------ATA GTTCTA-TGC
Mus_pahari               CTTTTTT--- ---------- ---------- ---TTTTTTT TTTTTTAATA GTTCTA-TGC
Praomys                  TTTTTTT--- ---------- ---------- ---------- -------ATA GTTCTA-TGC
Meriones                 CCTTTTT--- ---------- ---------- ---------- -------ATG GTTCTA-TGC
Meriones_crassus         CCTTTTT--- ---------- ---------- ---------- -------ATA GTTCTA-TGC
Acomys cahirinus         ---------- ---------- ---------- ---------- ---------- ----------
Lophuromys sikapusi      ---------- ---------- ---------- ---------- ---------- ----------
Mesocricetus_auratus     CTTTTT---- ---------- ---------- ---------- -------ATA GTGCTA-TGA
Oryzomys                 CTTTTT---- ---------- ---------- ---------- -------ATA GTTCTA-TGG
Trichys_fasciculata      --TTTTT--- ---------- ---------- ---------- -------ATG ATTCTG-TTC
Heterocephalus_glaber    --TTTTT--- ---------- ---------- ---------- -------ATA TTTCTG-TTC
Coendou_melanurus        --TTTTT--- ---------- ---------- ---------- -------ATG TTTCTG-TTC
Cavia_porcellus          --TTTTT--- ---------- ---------- ---------- -------ATG TTTCTG-TTC
Ctenomys_maulinus        --TTTTT--- ---------- ---------- ---------- -------ACT TTTCTG-TTC
Octodon_degus            --TTTTT--- ---------- ---------- ---------- -------ACT TTTCTG-TTC
Loxodonta_africana       GATTTTT--- ---------- ---------- ---------- -------GTC CTTCTG-TTC
Procavia_capensis        GATTTTT--- ---------- ---------- ---------- -------ATT CTTCTG-TTC
Echinops_telfairi        GACTGCA--- ---------- ---------- ---------- --GGTCAGTT ACCGGGGCTC
Micropotamogale          GGACTTC--- ---------- ---------- ---------- -------AAA A-TCTC----
Myotis_lucifugus         A-TTCCT--- ---------- ---------- ---------- -------ATC TTTCTA-TTC
Canis_familiaris         A-T--CT--- ---------- ---------- ---------- -------GTC TTTCCT-TTC
Felis_catus              ---------- ---------- ---------- ---------- -------GTC TTTCCA-TCC
Ovis_aries               ---------- ---------- ---------- ---------- ---------- ----------
Equus_caballus           ---------- ---------- ---------- ---------- ---------- ----------
Tapirus_terrestris       A-TCCCT--- ---------- ---------- ---------- -------ATC CTTCTG-TTC
Vicugna                  --TTCCT--- ---------- ---------- ---------- -------ATC TTTCTG-TTC
Sus_scrofa               A-TTCCT--- ---------- ---------- ---------- -------AAC TTTCTC-TTC
Bos_taurus               A-TTTCT--- ---------- ---------- ---------- -------ACC TTTCAA-TTC
Tursiops_truncatus       G-TTCCT--- ---------- ---------- ---------- -------CTC TTTCAA-TTC
Erinaceus                A-TTCCT--- ---------- ---------- ---------- -------ATC TTTCAA-TGC
Sorex_araneus
Neomys_anomalus          G-TTTCT--- ---------- ---------- ---------- -------ACC TTTCTG-TTC
Choloepus_hoffmanni      A-TTTTT--- ---------- ---------- ---------- -------ATC TTTCTG-TTC
Dasypus                  A-TTTTC--- ---------- ---------- ---------- -------ATC TTTCCG-TGC
```

```
                         1141
Monodelphis              C--------- --TAAATATG AATGGCATGA AAGAA----- ---------- ----------
Macropus                 C--------- --TAAATATG AATGGCAATT AGGGA----- ---------- ----------
Microcebus_murinus       C--------- --CAAGTAGC TG-GGACTAC AGGCA----- ---------- ----------
Tarsius_syrichta         T--------- --CATGAGTC GA-GGCAGAT ACAGA----- ---------- ----------
Callithrix_jacchus       T--------- --TATGTGCG AA-GACAGAG AAAAA----- ---------- ----------
Macaca_mulatta           T--------- --TATGTGCG AA-GGCAGAG AAAGA----- ---------- ----------
Pongo_pygmaeus           T--------- --TATGTGTG AA-GGCAGAG AAAGA----- ---------- ----------
Gorilla_gorilla          ---------- ---------- ---------- ---------- ---------- ----------
Homo_sapiens             T--------- --TATGTGTG AA-GGCAGAG AAAGA----- ---------- ----------
Pan_troglodytes          T--------- --TATGTGTG AA-GGCAGAG AAAGA----- ---------- ----------
Ochotona_princeps        T--------- --TAAGTGTA AA-GACAGGT ATCAA----- ---------- ----------
Oryctolagus_cuniculus    T--------- --TAGGTATG AA-GGTAT-- --CAA----- ---------- ----------
Marmota_monax            T--------- --TATTTATG AA-GGCAGAC ATAGA----- ---------- ----------
Aplodantia_rufa          ---------- --CATATATA AA-GGCAGAT ATAGA----- ---------- ----------
Spermophilus             N--------- --NNNNNNNN NN-NNNNNNN NNNNN----- ---------- ----------
Anomamorus               T--------- --TATGTATG AA-GACATAT ATAGA----- ---------- ----------
Maxomys                  T--------- --TATGTCTA AA-GGCAGAT AGAGATTAGA ---------- ----------
Rattus rattus            T--------- --TATGTCTA AA-GGCAGAT ATTGA----- ---------- ----------
Rattus exulans           T--------- --TATGTCTA AA-GGCAGAT ATTGA----- ---------- ----------
Rattus_norvegicus        T--------- --TATGTCTA AA-GGCAGAT ATTAA----- ---------- ----------
Mus_musculus             T--------- --TATGTCCA A--GGCAGAT ATAGA----- ---------- ----------
Mus_spretus              T--------- --TATGTCCA A--GGCAGAT ATAGA----- ---------- ----------
Mus_pahari               T--------- --TATGTTCA AG-GGCAGAT ATAGA----- ---------- ----------
Praomys                  G--------- --TCTGTCCA AG-GACAGAT GTAGA----- ---------- ----------
Meriones                 T--------- --TATGTCCA AG-GTCATAT ACAGA----- ---------- ----------
Meriones_crassus         T--------- --TATGTCCA AG-GTCAGAT ACAGA----- ---------- ----------
Acomys cahirinus         ---------- ---------- ---------- ---------- ---------- ----------
Lophuromys sikapusi      ---------- ---------- ---------- ---------- ---------- ----------
Mesocricetus_auratus     T--------- --TCTGTTCA AG-GGCAGAT GTAGA----- ---------- ----------
Oryzomys                 C--------- --TATGTCCA AG-GGCAGCT ATAGA----- ---------- ----------
Trichys_fasciculata      C--------- --TCTGTATG AA-GGCAGAT ATGGA----- ---------- ----------
Heterocephalus_glaber    T--------- --C---TGTG AA-GGTGGAT ATAGA----- ---------- ----------
Coendou_melanurus        T--------- --TCTGTATG AA-GGCAGAT ATAGA----- ---------- ----------
Cavia_porcellus          T--------- --TCTGTATG AA-GGTAGAC ATAGA----- ---------- ----------
Ctenomys_maulinus        C--------- --TCTGTATG AA-AGCAG-- ATAGA----- ---------- ----------
Octodon_degus            C--------- --TCTGTATG AA-AGCTG-- ATAGA----- ---------- ----------
Loxodonta_africana       T--------- --TACCGAGG AA-GGCA--- ACAGA----- ---------- ----------
Procavia_capensis        T--------- --TATTTATG GA-GACA--- ACAGA----- ---------- ----------
Echinops_telfairi        TGGCTCAGAA CAGATGCATG GG-GGAGGGG GTAGT----- ---------- ----------
Micropotamogale          ---------- --------TC AAGGATT--- CCAGT----- ---------- ----------
Myotis_lucifugus         TGTTTTCTCT CATCAGTGTT AT-TGTGTCC ATTAANNNNN NNNNNNNNNN NNNNNNNNNN
Canis_familiaris         T--------- --CATATGCC GA-GGCAAAC ATGGA----- ---------- ----------
Felis_catus              T--------- --CACCTGCC AA-GGCCGAT GTGGA----- ---------- ----------
Ovis_aries               ---------- ---------- ---------- ---------- ---------- ----------
Equus_caballus           ---------- ---------- ---------- ---------- ---------- ----------
Tapirus_terrestris       T--------- --TATGTGTG AA-AGCAGAM ATAGT----- ---------- ----------
Vicugna                  T--------- --TCTGTGCG AA-GGCAGAC ATAGA----- ---------- ----------
Sus_scrofa               T--------- --TGGGTGCG AA-GACAT-- --AGA----- ---------- ----------
Bos_taurus               T--------- --TACCTGTG AA-GAAAGAC AGAGA----- ---------- ----------
Tursiops_truncatus       T--------- --TATGTGTG AA-GACAA-- --AGG----- ---------- ----------
Erinaceus                T--------- --TAGATTCA AA-GGCAG-- GAAGA
Sorex_araneus
Neomys_anomalus          TATGGGTGCA -GGCCATCAT AA-CTCAG-- ---------- ---------- ----------
Choloepus_hoffmanni      T--------- --T--GTGTG AA-GGCAGGC ATAGA----- ---------- ----------
Dasypus                  T--------- --T--GCGTG AA-GGCAGAC ATTGA
```

```
                    1201
Monodelphis           ---------- ---------- ---------- ---------- ---------- ----------
Macropus              ---------- ---------- ---------- ---------- ---------- ----------
Microcebus_murinus    ---------- ---------- ---------- ---------- ---------- ----------
Tarsius_syrichta      ---------- ---------- ---------- ---------- ---------- ----------
Callithrix_jacchus    ---------- ---------- ---------- ---------- ---------- ----------
Macaca_mulatta        ---------- ---------- ---------- ---------- ---------- ----------
Pongo_pygmaeus        ---------- ---------- ---------- ---------- ---------- ----------
Gorilla_gorilla       ---------- ---------- ---------- ---------- ---------- ----------
Homo_sapiens          ---------- ---------- ---------- ---------- ---------- ----------
Pan_troglodytes       ---------- ---------- ---------- ---------- ---------- ----------
Ochotona_princeps     ---------- ---------- ---------- ---------- ---------- ----------
Oryctolagus_cuniculus ---------- ---------- ---------- ---------- ---------- ----------
Marmota_monax         ---------- ---------- ---------- ---------- ---------- ----------
Aplodantia_rufa       ---------- ---------- ---------- ---------- ---------- ----------
Spermophilus          ---------- ---------- ---------- ---------- ---------- ----------
Anomamorus            ---------- ---------- ---------- ---------- ---------- ----------
Maxomys               ---------- ---------- ---------- ---------- ---------- ----------
Rattus rattus         ---------- ---------- ---------- ---------- ---------- ----------
Rattus exulans        ---------- ---------- ---------- ---------- ---------- ----------
Rattus_norvegicus     ---------- ---------- ---------- ---------- ---------- ----------
Mus_musculus          ---------- ---------- ---------- ---------- ---------- ----------
Mus_spretus           ---------- ---------- ---------- ---------- ---------- ----------
Mus_pahari            ---------- ---------- ---------- ---------- ---------- ----------
Praomys               ---------- ---------- ---------- ---------- ---------- ----------
Meriones              ---------- ---------- ---------- ---------- ---------- ----------
Meriones_crassus      ---------- ---------- ---------- ---------- ---------- ----------
Acomys cahirinus      ---------- ---------- ---------- ---------- ---------- ----------
Lophuromys sikapusi   ---------- ---------- ---------- ---------- ---------- ----------
Mesocricetus_auratus  ---------- ---------- ---------- ---------- ---------- ----------
Oryzomys              ---------- ---------- ---------- ---------- ---------- ----------
Trichys_fasciculata   ---------- ---------- ---------- ---------- ---------- ----------
Heterocephalus_glaber ---------- ---------- ---------- ---------- ---------- ----------
Coendou_melanurus     ---------- ---------- ---------- ---------- ---------- ----------
Cavia_porcellus       ---------- ---------- ---------- ---------- ---------- ----------
Ctenomys_maulinus     ---------- ---------- ---------- ---------- ---------- ----------
Octodon_degus         ---------- ---------- ---------- ---------- ---------- ----------
Loxodonta_africana    ---------- ---------- ---------- ---------- ---------- ----------
Procavia_capensis     ---------- ---------- ---------- ---------- ---------- ----------
Echinops_telfairi     ---------- ---------- ---------- ---------- ---------- ----------
Micropotamogale       ---------- ---------- ---------- ---------- ---------- ----------
Myotis_lucifugus      NNNNNNNNNN NNNNNNNNNN NNNNNNNNNN NNNNNNNNNN NNNNNNNNNN NNNNNNNNNN
Canis_familiaris      ---------- ---------- ---------- ---------- ---------- ----------
Felis_catus           ---------- ---------- ---------- ---------- ---------- ----------
Ovis_aries            ---------- ---------- ---------- ---------- ---------- ----------
Equus_caballus        ---------- ---------- ---------- ---------- ---------- ----------
Tapirus_terrestris    ---------- ---------- ---------- ---------- ---------- ----------
Vicugna               ---------- ---------- ---------- ---------- ---------- ----------
Sus_scrofa            ---------- ---------- ---------- ---------- ---------- ----------
Bos_taurus            ---------- ---------- ---------- ---------- ---------- ----------
Tursiops_truncatus    ---------- ---------- ---------- ---------- ---------- ----------
Erinaceus             ---------- ---------- ---------- ---------- ---------- ----------
Sorex_araneus
Neomys_anomalus       ---------- ---------- ---------- ---------- ---------- ----------
Choloepus_hoffmanni   ---------- ---------- ---------- ---------- ---------- ----------
Dasypus               ---------- ---------- ---------- ---------- ---------- ----------
```

```
                    1261
Monodelphis           ---------- ---------- ---------- ---------- ---------- ----------
Macropus              ---------- ---------- ---------- ---------- ---------- ----------
Microcebus_murinus    ---------- ---------- ---------- ---------- ---------- ----------
Tarsius_syrichta      ---------- ---------- ---------- ---------- ---------- ----------
Callithrix_jacchus    ---------- ---------- ---------- ---------- ---------- ----------
Macaca_mulatta        ---------- ---------- ---------- ---------- ---------- ----------
Pongo_pygmaeus        ---------- ---------- ---------- ---------- ---------- ----------
Gorilla_gorilla       ---------- ---------- ---------- ---------- ---------- ----------
Homo_sapiens          ---------- ---------- ---------- ---------- ---------- ----------
Pan_troglodytes       ---------- ---------- ---------- ---------- ---------- ----------
Ochotona_princeps     ---------- ---------- ---------- ---------- ---------- ----------
Oryctolagus_cuniculus ---------- ---------- ---------- ---------- ---------- ----------
Marmota_monax         ---------- ---------- ---------- ---------- ---------- ----------
Aplodantia_rufa       ---------- ---------- ---------- ---------- ---------- ----------
Spermophilus          ---------- ---------- ---------- ---------- ---------- ----------
Anomamorus            ---------- ---------- ---------- ---------- ---------- ----------
Maxomys               ---------- ---------- ---------- ---------- ---------- ----------
Rattus rattus         ---------- ---------- ---------- ---------- ---------- ----------
Rattus exulans        ---------- ---------- ---------- ---------- ---------- ----------
Rattus_norvegicus     ---------- ---------- ---------- ---------- ---------- ----------
Mus_musculus          ---------- ---------- ---------- ---------- ---------- ----------
Mus_spretus           ---------- ---------- ---------- ---------- ---------- ----------
Mus_pahari            ---------- ---------- ---------- ---------- ---------- ----------
Praomys               ---------- ---------- ---------- ---------- ---------- ----------
Meriones              ---------- ---------- ---------- ---------- ---------- ----------
Meriones_crassus      ---------- ---------- ---------- ---------- ---------- ----------
Acomys cahirinus      ---------- ---------- ---------- ---------- ---------- ----------
Lophuromys sikapusi   ---------- ---------- ---------- ---------- ---------- ----------
Mesocricetus_auratus  ---------- ---------- ---------- ---------- ---------- ----------
Oryzomys              ---------- ---------- ---------- ---------- ---------- ----------
Trichys_fasciculata   ---------- ---------- ---------- ---------- ---------- ----------
Heterocephalus_glaber ---------- ---------- ---------- ---------- ---------- ----------
Coendou_melanurus     ---------- ---------- ---------- ---------- ---------- ----------
Cavia_porcellus       ---------- ---------- ---------- ---------- ---------- ----------
Ctenomys_maulinus     ---------- ---------- ---------- ---------- ---------- ----------
Octodon_degus         ---------- ---------- ---------- ---------- ---------- ----------
Loxodonta_africana    ---------- ---------- ---------- ---------- ---------- ----------
Procavia_capensis     ---------- ---------- ---------- ---------- ---------- ----------
Echinops_telfairi     ---------- ---------- ---------- ---------- ---------- ----------
Micropotamogale       ---------- ---------- ---------- ---------- ---------- ----------
Myotis_lucifugus      NNNNNNNNNN NNNNNNNNNN NNNNNNNNNN NNNNNNNNNN NNNNNNNNNN NNNNNNNNNN
Canis_familiaris      ---------- ---------- ---------- ---------- ---------- ----------
Felis_catus           ---------- ---------- ---------- ---------- ---------- ----------
Ovis_aries            ---------- ---------- ---------- ---------- ---------- ----------
Equus_caballus        ---------- ---------- ---------- ---------- ---------- ----------
Tapirus_terrestris    ---------- ---------- ---------- ---------- ---------- ----------
Vicugna               ---------- ---------- ---------- ---------- ---------- ----------
Sus_scrofa            ---------- ---------- ---------- ---------- ---------- ----------
Bos_taurus            ---------- ---------- ---------- ---------- ---------- ----------
Tursiops_truncatus    ---------- ---------- ---------- ---------- ---------- ----------
Erinaceus             ---------- ---------- ---------- ---------- ---------- ----------
Sorex_araneus
Neomys_anomalus       ---------- ---------- ---------- ---------- ---------- ----------
Choloepus_hoffmanni   ---------- ---------- ---------- ---------- ---------- ----------
Dasypus               ---------- ---------- ---------- ---------- ---------- ----------
```

```
                    1321
Monodelphis             ---------- ---------- ---------- ---------- ---------- ----------
Macropus                ---------- ---------- ---------- ---------- ---------- ----------
Microcebus_murinus      ---------- ---------- ---------- ---------- ---------- ----------
Tarsius_syrichta        ---------- ---------- ---------- ---------- ---------- ----------
Callithrix_jacchus      ---------- ---------- ---------- ---------- ---------- ----------
Macaca_mulatta          ---------- ---------- ---------- ---------- ---------- ----------
Pongo_pygmaeus          ---------- ---------- ---------- ---------- ---------- ----------
Gorilla_gorilla         ---------- ---------- ---------- ---------- ---------- ----------
Homo_sapiens            ---------- ---------- ---------- ---------- ---------- ----------
Pan_troglodytes         ---------- ---------- ---------- ---------- ---------- ----------
Ochotona_princeps       ---------- ---------- ---------- ---------- ---------- ----------
Oryctolagus_cuniculus   ---------- ---------- ---------- ---------- ---------- ----------
Marmota_monax           ---------- ---------- ---------- ---------- ---------- ----------
Aplodantia_rufa         ---------- ---------- ---------- ---------- ---------- ----------
Spermophilus            ---------- ---------- ---------- ---------- ---------- ----------
Anomamorus              ---------- ---------- ---------- ---------- ---------- ----------
Maxomys                 ---------- ---------- ---------- ---------- ---------- ----------
Rattus rattus           ---------- ---------- ---------- ---------- ---------- ----------
Rattus exulans          ---------- ---------- ---------- ---------- ---------- ----------
Rattus_norvegicus       ---------- ---------- ---------- ---------- ---------- ----------
Mus_musculus            ---------- ---------- ---------- ---------- ---------- ----------
Mus_spretus             ---------- ---------- ---------- ---------- ---------- ----------
Mus_pahari              ---------- ---------- ---------- ---------- ---------- ----------
Praomys                 ---------- ---------- ---------- ---------- ---------- ----------
Meriones                ---------- ---------- ---------- ---------- ---------- ----------
Meriones_crassus        ---------- ---------- ---------- ---------- ---------- ----------
Acomys cahirinus        ---------- ---------- ---------- ---------- ---------- ----------
Lophuromys sikapusi     ---------- ---------- ---------- ---------- ---------- ----------
Mesocricetus_auratus    ---------- ---------- ---------- ---------- ---------- ----------
Oryzomys                ---------- ---------- ---------- ---------- ---------- ----------
Trichys_fasciculata     ---------- ---------- ---------- ---------- ---------- ----------
Heterocephalus_glaber   ---------- ---------- ---------- ---------- ---------- ----------
Coendou_melanurus       ---------- ---------- ---------- ---------- ---------- ----------
Cavia_porcellus         ---------- ---------- ---------- ---------- ---------- ----------
Ctenomys_maulinus       ---------- ---------- ---------- ---------- ---------- ----------
Octodon_degus           ---------- ---------- ---------- ---------- ---------- ----------
Loxodonta_africana      ---------- ---------- ---------- ---------- ---------- ----------
Procavia_capensis       ---------- ---------- ---------- ---------- ---------- ----------
Echinops_telfairi       ---------- ---------- ---------- ---------- ---------- ----------
Micropotamogale         ---------- ---------- ---------- ---------- ---------- ----------
Myotis_lucifugus        NNNNNNNNNN NNNNNNNNNN NNNNNNNNNN NNNNNNNNNN NNNNNNNNNN NNNNNNNNNN
Canis_familiaris        ---------- ---------- ---------- ---------- ---------- ----------
Felis_catus             ---------- ---------- ---------- ---------- ---------- ----------
Ovis_aries              ---------- ---------- ---------- ---------- ---------- ----------
Equus_caballus          ---------- ---------- ---------- ---------- ---------- ----------
Tapirus_terrestris      ---------- ---------- ---------- ---------- ---------- ----------
Vicugna                 ---------- ---------- ---------- ---------- ---------- ----------
Sus_scrofa              ---------- ---------- ---------- ---------- ---------- ----------
Bos_taurus              ---------- ---------- ---------- ---------- ---------- ----------
Tursiops_truncatus      ---------- ---------- ---------- ---------- ---------- ----------
Erinaceus               ---------- ---------- ---------- ---------- ---------- ----------
Sorex_araneus
Neomys_anomalus         ---------- ---------- ---------- ---------- ---------- ----------
Choloepus_hoffmanni     ---------- ---------- ---------- ---------- ---------- ----------
Dasypus                 ---------- ---------- ---------- ---------- ---------- ----------
```

```
                    1381
Monodelphis            ---------- ---------- ---------- ---------- ---------- ----------
Macropus               ---------- ---------- ---------- ---------- ---------- ----------
Microcebus_murinus     ---------- ---------- ---------- ---------- ---------- ----------
Tarsius_syrichta       ---------- ---------- ---------- ---------- ---------- ----------
Callithrix_jacchus     ---------- ---------- ---------- ---------- ---------- ----------
Macaca_mulatta         ---------- ---------- ---------- ---------- ---------- ----------
Pongo_pygmaeus         ---------- ---------- ---------- ---------- ---------- ----------
Gorilla_gorilla        ---------- ---------- ---------- ---------- ---------- ----------
Homo_sapiens           ---------- ---------- ---------- ---------- ---------- ----------
Pan_troglodytes        ---------- ---------- ---------- ---------- ---------- ----------
Ochotona_princeps      ---------- ---------- ---------- ---------- ---------- ----------
Oryctolagus_cuniculus  ---------- ---------- ---------- ---------- ---------- ----------
Marmota_monax          ---------- ---------- ---------- ---------- ---------- ----------
Aplodantia_rufa        ---------- ---------- ---------- ---------- ---------- ----------
Spermophilus           ---------- ---------- ---------- ---------- ---------- ----------
Anomamorus             ---------- ---------- ---------- ---------- ---------- ----------
Maxomys                ---------- ---------- ---------- ---------- ---------- ----------
Rattus rattus          ---------- ---------- ---------- ---------- ---------- ----------
Rattus exulans         ---------- ---------- ---------- ---------- ---------- ----------
Rattus_norvegicus      ---------- ---------- ---------- ---------- ---------- ----------
Mus_musculus           ---------- ---------- ---------- ---------- ---------- ----------
Mus_spretus            ---------- ---------- ---------- ---------- ---------- ----------
Mus_pahari             ---------- ---------- ---------- ---------- ---------- ----------
Praomys                ---------- ---------- ---------- ---------- ---------- ----------
Meriones               ---------- ---------- ---------- ---------- ---------- ----------
Meriones_crassus       ---------- ---------- ---------- ---------- ---------- ----------
Acomys cahirinus       ---------- ---------- ---------- ---------- ---------- ----------
Lophuromys sikapusi    ---------- ---------- ---------- ---------- ---------- ----------
Mesocricetus_auratus   ---------- ---------- ---------- ---------- ---------- ----------
Oryzomys               ---------- ---------- ---------- ---------- ---------- ----------
Trichys_fasciculata    ---------- ---------- ---------- ---------- ---------- ----------
Heterocephalus_glaber  ---------- ---------- ---------- ---------- ---------- ----------
Coendou_melanurus      ---------- ---------- ---------- ---------- ---------- ----------
Cavia_porcellus        ---------- ---------- ---------- ---------- ---------- ----------
Ctenomys_maulinus      ---------- ---------- ---------- ---------- ---------- ----------
Octodon_degus          ---------- ---------- ---------- ---------- ---------- ----------
Loxodonta_africana     ---------- ---------- ---------- ---------- ---------- ----------
Procavia_capensis      ---------- ---------- ---------- ---------- ---------- ----------
Echinops_telfairi      ---------- ---------- ---------- ---------- ---------- ----------
Micropotamogale        ---------- ---------- ---------- ---------- ---------- ----------
Myotis_lucifugus       NNNNNNNNNN NNNNNNNNNN NNNNNNNNNN NNNNNNNNNN NNNNNNNNNN NNNNNNNNNN
Canis_familiaris       ---------- ---------- ---------- ---------- ---------- ----------
Felis_catus            ---------- ---------- ---------- ---------- ---------- ----------
Ovis_aries             ---------- ---------- ---------- ---------- ---------- ----------
Equus_caballus         ---------- ---------- ---------- ---------- ---------- ----------
Tapirus_terrestris     ---------- ---------- ---------- ---------- ---------- ----------
Vicugna                ---------- ---------- ---------- ---------- ---------- ----------
Sus_scrofa             ---------- ---------- ---------- ---------- ---------- ----------
Bos_taurus             ---------- ---------- ---------- ---------- ---------- ----------
Tursiops_truncatus     ---------- ---------- ---------- ---------- ---------- ----------
Erinaceus              ---------- ---------- ---------- ---------- ---------- ----------
Sorex_araneus
Neomys_anomalus        ---------- ---------- ---------- ---------- ---------- ----------
Choloepus_hoffmanni    ---------- ---------- ---------- ---------- ---------- ----------
Dasypus                ---------- ---------- ---------- ---------- ---------- ----------
```

```
                        1441
Monodelphis             ---------- ---------- ---------- ---------- ---------- ----------
Macropus                ---------- ---------- ---------- ---------- ---------- ----------
Microcebus_murinus      ---------- ---------- ---------- ---------- ---------- ----------
Tarsius_syrichta        ---------- ---------- ---------- ---------- ---------- ----------
Callithrix_jacchus      ---------- ---------- ---------- ---------- ---------- ----------
Macaca_mulatta          ---------- ---------- ---------- ---------- ---------- ----------
Pongo_pygmaeus          ---------- ---------- ---------- ---------- ---------- ----------
Gorilla_gorilla         ---------- ---------- ---------- ---------- ---------- ----------
Homo_sapiens            ---------- ---------- ---------- ---------- ---------- ----------
Pan_troglodytes         ---------- ---------- ---------- ---------- ---------- ----------
Ochotona_princeps       ---------- ---------- ---------- ---------- ---------- ----------
Oryctolagus_cuniculus   ---------- ---------- ---------- ---------- ---------- ----------
Marmota_monax           ---------- ---------- ---------- ---------- ---------- ----------
Aplodantia_rufa         ---------- ---------- ---------- ---------- ---------- ----------
Spermophilus            ---------- ---------- ---------- ---------- ---------- ----------
Anomamorus              ---------- ---------- ---------- ---------- ---------- ----------
Maxomys                 ---------- ---------- ---------- ---------- ---------- ----------
Rattus rattus           ---------- ---------- ---------- ---------- ---------- ----------
Rattus exulans          ---------- ---------- ---------- ---------- ---------- ----------
Rattus_norvegicus       ---------- ---------- ---------- ---------- ---------- ----------
Mus_musculus            ---------- ---------- ---------- ---------- ---------- ----------
Mus_spretus             ---------- ---------- ---------- ---------- ---------- ----------
Mus_pahari              ---------- ---------- ---------- ---------- ---------- ----------
Praomys                 ---------- ---------- ---------- ---------- ---------- ----------
Meriones                ---------- ---------- ---------- ---------- ---------- ----------
Meriones_crassus        ---------- ---------- ---------- ---------- ---------- ----------
Acomys cahirinus        ---------- ---------- ---------- ---------- ---------- ----------
Lophuromys sikapusi     ---------- ---------- ---------- ---------- ---------- ----------
Mesocricetus_auratus    ---------- ---------- ---------- ---------- ---------- ----------
Oryzomys                ---------- ---------- ---------- ---------- ---------- ----------
Trichys_fasciculata     ---------- ---------- ---------- ---------- ---------- ----------
Heterocephalus_glaber   ---------- ---------- ---------- ---------- ---------- ----------
Coendou_melanurus       ---------- ---------- ---------- ---------- ---------- ----------
Cavia_porcellus         ---------- ---------- ---------- ---------- ---------- ----------
Ctenomys_maulinus       ---------- ---------- ---------- ---------- ---------- ----------
Octodon_degus           ---------- ---------- ---------- ---------- ---------- ----------
Loxodonta_africana      ---------- ---------- ---------- ---------- ---------- ----------
Procavia_capensis       ---------- ---------- ---------- ---------- ---------- ----------
Echinops_telfairi       ---------- ---------- ---------- ---------- ---------- ----------
Micropotamogale         ---------- ---------- ---------- ---------- ---------- ----------
Myotis_lucifugus        NNNNNNNNNN NNNNNNNNNN NNNNNNNNNN NNNNNNNNNN NNNNNNNNNN NNNNNNNNNN
Canis_familiaris        ---------- ---------- ---------- ---------- ---------- ----------
Felis_catus             ---------- ---------- ---------- ---------- ---------- ----------
Ovis_aries              ---------- ---------- ---------- ---------- ---------- ----------
Equus_caballus          ---------- ---------- ---------- ---------- ---------- ----------
Tapirus_terrestris      ---------- ---------- ---------- ---------- ---------- ----------
Vicugna                 ---------- ---------- ---------- ---------- ---------- ----------
Sus_scrofa              ---------- ---------- ---------- ---------- ---------- ----------
Bos_taurus              ---------- ---------- ---------- ---------- ---------- ----------
Tursiops_truncatus      ---------- ---------- ---------- ---------- ---------- ----------
Erinaceus               ---------- ---------- ---------- ---------- ---------- ----------
Sorex_araneus
Neomys_anomalus         ---------- ---------- ---------- ---------- ---------- ----------
Choloepus_hoffmanni     ---------- ---------- ---------- ---------- ---------- ----------
Dasypus                 ---------- ---------- ---------- ---------- ---------- ----------
```

```
                    1501
Monodelphis            ---------- ---------- ---------- ---------- ---------- ----------
Macropus               ---------- ---------- ---------- ---------- ---------- ----------
Microcebus_murinus     ---------- ---------- ---------- ---------- ---------- ----------
Tarsius_syrichta       ---------- ---------- ---------- ---------- ---------- ----------
Callithrix_jacchus     ---------- ---------- ---------- ---------- ---------- ----------
Macaca_mulatta         ---------- ---------- ---------- ---------- ---------- ----------
Pongo_pygmaeus         ---------- ---------- ---------- ---------- ---------- ----------
Gorilla_gorilla        ---------- ---------- ---------- ---------- ---------- ----------
Homo_sapiens           ---------- ---------- ---------- ---------- ---------- ----------
Pan_troglodytes        ---------- ---------- ---------- ---------- ---------- ----------
Ochotona_princeps      ---------- ---------- ---------- ---------- ---------- ----------
Oryctolagus_cuniculus  ---------- ---------- ---------- ---------- ---------- ----------
Marmota_monax          ---------- ---------- ---------- ---------- ---------- ----------
Aplodantia_rufa        ---------- ---------- ---------- ---------- ---------- ----------
Spermophilus           ---------- ---------- ---------- ---------- ---------- ----------
Anomamorus             ---------- ---------- ---------- ---------- ---------- ----------
Maxomys                ---------- ---------- ---------- ---------- ---------- ----------
Rattus rattus          ---------- ---------- ---------- ---------- ---------- ----------
Rattus exulans         ---------- ---------- ---------- ---------- ---------- ----------
Rattus_norvegicus      ---------- ---------- ---------- ---------- ---------- ----------
Mus_musculus           ---------- ---------- ---------- ---------- ---------- ----------
Mus_spretus            ---------- ---------- ---------- ---------- ---------- ----------
Mus_pahari             ---------- ---------- ---------- ---------- ---------- ----------
Praomys                ---------- ---------- ---------- ---------- ---------- ----------
Meriones               ---------- ---------- ---------- ---------- ---------- ----------
Meriones_crassus       ---------- ---------- ---------- ---------- ---------- ----------
Acomys cahirinus       ---------- ---------- ---------- ---------- ---------- ----------
Lophuromys sikapusi    ---------- ---------- ---------- ---------- ---------- ----------
Mesocricetus_auratus   ---------- ---------- ---------- ---------- ---------- ----------
Oryzomys               ---------- ---------- ---------- ---------- ---------- ----------
Trichys_fasciculata    ---------- ---------- ---------- ---------- ---------- ----------
Heterocephalus_glaber  ---------- ---------- ---------- ---------- ---------- ----------
Coendou_melanurus      ---------- ---------- ---------- ---------- ---------- ----------
Cavia_porcellus        ---------- ---------- ---------- ---------- ---------- ----------
Ctenomys_maulinus      ---------- ---------- ---------- ---------- ---------- ----------
Octodon_degus          ---------- ---------- ---------- ---------- ---------- ----------
Loxodonta_africana     ---------- ---------- ---------- ---------- ---------- ----------
Procavia_capensis      ---------- ---------- ---------- ---------- ---------- ----------
Echinops_telfairi      ---------- ---------- ---------- ---------- ---------- ----------
Micropotamogale        ---------- ---------- ---------- ---------- ---------- ----------
Myotis_lucifugus       NNNNNNNNNN NNNNNNNNNN NNNNNNNNNN NNNNNNNNNN NNNNNNNNNN NNNNNNNNNN
Canis_familiaris       ---------- ---------- ---------- ---------- ---------- ----------
Felis_catus            ---------- ---------- ---------- ---------- ---------- ----------
Ovis_aries             ---------- ---------- ---------- ---------- ---------- ----------
Equus_caballus         ---------- ---------- ---------- ---------- ---------- ----------
Tapirus_terrestris     ---------- ---------- ---------- ---------- ---------- ----------
Vicugna                ---------- ---------- ---------- ---------- ---------- ----------
Sus_scrofa             ---------- ---------- ---------- ---------- ---------- ----------
Bos_taurus             ---------- ---------- ---------- ---------- ---------- ----------
Tursiops_truncatus     ---------- ---------- ---------- ---------- ---------- ----------
Erinaceus              ---------- ---------- ---------- ---------- ---------- ----------
Sorex_araneus
Neomys_anomalus        ---------- ---------- ---------- ---------- ---------- ----------
Choloepus_hoffmanni    ---------- ---------- ---------- ---------- ---------- ----------
Dasypus                ---------- ---------- ---------- ---------- ---------- ----------
```

```
                        1561
Monodelphis             ---------- ---------- ---------- ---------- ---------- ----------
Macropus                ---------- ---------- ---------- ---------- ---------- ----------
Microcebus_murinus      ---------- ---------- ---------- ---------- ---------- ----------
Tarsius_syrichta        ---------- ---------- ---------- ---------- ---------- ----------
Callithrix_jacchus      ---------- ---------- ---------- ---------- ---------- ----------
Macaca_mulatta          ---------- ---------- ---------- ---------- ---------- ----------
Pongo_pygmaeus          ---------- ---------- ---------- ---------- ---------- ----------
Gorilla_gorilla         ---------- ---------- ---------- ---------- ---------- ----------
Homo_sapiens            ---------- ---------- ---------- ---------- ---------- ----------
Pan_troglodytes         ---------- ---------- ---------- ---------- ---------- ----------
Ochotona_princeps       ---------- ---------- ---------- ---------- ---------- ----------
Oryctolagus_cuniculus   ---------- ---------- ---------- ---------- ---------- ----------
Marmota_monax           ---------- ---------- ---------- ---------- ---------- ----------
Aplodantia_rufa         ---------- ---------- ---------- ---------- ---------- ----------
Spermophilus            ---------- ---------- ---------- ---------- ---------- ----------
Anomamorus              ---------- ---------- ---------- ---------- ---------- ----------
Maxomys                 ---------- ---------- ---------- ---------- ---------- ----------
Rattus rattus           ---------- ---------- ---------- ---------- ---------- ----------
Rattus exulans          ---------- ---------- ---------- ---------- ---------- ----------
Rattus_norvegicus       ---------- ---------- ---------- ---------- ---------- ----------
Mus_musculus            ---------- ---------- ---------- ---------- ---------- ----------
Mus_spretus             ---------- ---------- ---------- ---------- ---------- ----------
Mus_pahari              ---------- ---------- ---------- ---------- ---------- ----------
Praomys                 ---------- ---------- ---------- ---------- ---------- ----------
Meriones                ---------- ---------- ---------- ---------- ---------- ----------
Meriones_crassus        ---------- ---------- ---------- ---------- ---------- ----------
Acomys cahirinus        ---------- ---------- ---------- ---------- ---------- ----------
Lophuromys sikapusi     ---------- ---------- ---------- ---------- ---------- ----------
Mesocricetus_auratus    ---------- ---------- ---------- ---------- ---------- ----------
Oryzomys                ---------- ---------- ---------- ---------- ---------- ----------
Trichys_fasciculata     ---------- ---------- ---------- ---------- ---------- ----------
Heterocephalus_glaber   ---------- ---------- ---------- ---------- ---------- ----------
Coendou_melanurus       ---------- ---------- ---------- ---------- ---------- ----------
Cavia_porcellus         ---------- ---------- ---------- ---------- ---------- ----------
Ctenomys_maulinus       ---------- ---------- ---------- ---------- ---------- ----------
Octodon_degus           ---------- ---------- ---------- ---------- ---------- ----------
Loxodonta_africana      ---------- ---------- ---------- ---------- ---------- ----------
Procavia_capensis       ---------- ---------- ---------- ---------- ---------- ----------
Echinops_telfairi       ---------- ---------- ---------- ---------- ---------- ----------
Micropotamogale         ---------- ---------- ---------- ---------- ---------- ----------
Myotis_lucifugus        NNNNNNNNNN NNNNNNNNNN NNNNNNNNNN NNNNNNNNNN NNNNNNNNNN NNNNNNNNNN
Canis_familiaris        ---------- ---------- ---------- ---------- ---------- ----------
Felis_catus             ---------- ---------- ---------- ---------- ---------- ----------
Ovis_aries              ---------- ---------- ---------- ---------- ---------- ----------
Equus_caballus          ---------- ---------- ---------- ---------- ---------- ----------
Tapirus_terrestris      ---------- ---------- ---------- ---------- ---------- ----------
Vicugna                 ---------- ---------- ---------- ---------- ---------- ----------
Sus_scrofa              ---------- ---------- ---------- ---------- ---------- ----------
Bos_taurus              ---------- ---------- ---------- ---------- ---------- ----------
Tursiops_truncatus      ---------- ---------- ---------- ---------- ---------- ----------
Erinaceus               ---------- ---------- ---------- ---------- ---------- ----------
Sorex_araneus
Neomys_anomalus         ---------- ---------- ---------- ---------- ---------- ----------
Choloepus_hoffmanni     ---------- ---------- ---------- ---------- ---------- ----------
Dasypus                 ---------- ---------- ---------- ---------- ---------- ----------
```

```
                    1621
Monodelphis            ---------- ---------- ---------- ---------- ---------- ----------
Macropus               ---------- ---------- ---------- ---------- ---------- ----------
Microcebus_murinus     ---------- ---------- ---------- ---------- ---------- ----------
Tarsius_syrichta       ---------- ---------- ---------- ---------- ---------- ----------
Callithrix_jacchus     ---------- ---------- ---------- ---------- ---------- ----------
Macaca_mulatta         ---------- ---------- ---------- ---------- ---------- ----------
Pongo_pygmaeus         ---------- ---------- ---------- ---------- ---------- ----------
Gorilla_gorilla        ---------- ---------- ---------- ---------- ---------- ----------
Homo_sapiens           ---------- ---------- ---------- ---------- ---------- ----------
Pan_troglodytes        ---------- ---------- ---------- ---------- ---------- ----------
Ochotona_princeps      ---------- ---------- ---------- ---------- ---------- ----------
Oryctolagus_cuniculus  ---------- ---------- ---------- ---------- ---------- ----------
Marmota_monax          ---------- ---------- ---------- ---------- ---------- ----------
Aplodantia_rufa        ---------- ---------- ---------- ---------- ---------- ----------
Spermophilus           ---------- ---------- ---------- ---------- ---------- ----------
Anomamorus             ---------- ---------- ---------- ---------- ---------- ----------
Maxomys                ---------- ---------- ---------- ---------- ---------- ----------
Rattus rattus          ---------- ---------- ---------- ---------- ---------- ----------
Rattus exulans         ---------- ---------- ---------- ---------- ---------- ----------
Rattus_norvegicus      ---------- ---------- ---------- ---------- ---------- ----------
Mus_musculus           ---------- ---------- ---------- ---------- ---------- ----------
Mus_spretus            ---------- ---------- ---------- ---------- ---------- ----------
Mus_pahari             ---------- ---------- ---------- ---------- ---------- ----------
Praomys                ---------- ---------- ---------- ---------- ---------- ----------
Meriones               ---------- ---------- ---------- ---------- ---------- ----------
Meriones_crassus       ---------- ---------- ---------- ---------- ---------- ----------
Acomys cahirinus       ---------- ---------- ---------- ---------- ---------- ----------
Lophuromys sikapusi    ---------- ---------- ---------- ---------- ---------- ----------
Mesocricetus_auratus   ---------- ---------- ---------- ---------- ---------- ----------
Oryzomys               ---------- ---------- ---------- ---------- ---------- ----------
Trichys_fasciculata    ---------- ---------- ---------- ---------- ---------- ----------
Heterocephalus_glaber  ---------- ---------- ---------- ---------- ---------- ----------
Coendou_melanurus      ---------- ---------- ---------- ---------- ---------- ----------
Cavia_porcellus        ---------- ---------- ---------- ---------- ---------- ----------
Ctenomys_maulinus      ---------- ---------- ---------- ---------- ---------- ----------
Octodon_degus          ---------- ---------- ---------- ---------- ---------- ----------
Loxodonta_africana     ---------- ---------- ---------- ---------- ---------- ----------
Procavia_capensis      ---------- ---------- ---------- ---------- ---------- ----------
Echinops_telfairi      ---------- ---------- ---------- ---------- ---------- ----------
Micropotamogale        ---------- ---------- ---------- ---------- ---------- ----------
Myotis_lucifugus       NNNNNNNNNN NNNNNNNNNN NNNNNNNNNN NNNNNNNNNN NNNNNNNNNN NNNNNNNNNN
Canis_familiaris       ---------- ---------- ---------- ---------- ---------- ----------
Felis_catus            ---------- ---------- ---------- ---------- ---------- ----------
Ovis_aries             ---------- ---------- ---------- ---------- ---------- ----------
Equus_caballus         ---------- ---------- ---------- ---------- ---------- ----------
Tapirus_terrestris     ---------- ---------- ---------- ---------- ---------- ----------
Vicugna                ---------- ---------- ---------- ---------- ---------- ----------
Sus_scrofa             ---------- ---------- ---------- ---------- ---------- ----------
Bos_taurus             ---------- ---------- ---------- ---------- ---------- ----------
Tursiops_truncatus     ---------- ---------- ---------- ---------- ---------- ----------
Erinaceus              ---------- ---------- ---------- ---------- ---------- ----------
Sorex_araneus
Neomys_anomalus        ---------- ---------- ---------- ---------- ---------- ----------
Choloepus_hoffmanni    ---------- ---------- ---------- ---------- ---------- ----------
Dasypus                ---------- ---------- ---------- ---------- ---------- ----------
```

```
                        1681
Monodelphis                ---------- ---------- ---------- ---------- ---------- ----------
Macropus                   ---------- ---------- ---------- ---------- ---------- ----------
Microcebus_murinus         ---------- ---------- ---------- ---------- ---------- ----------
Tarsius_syrichta           ---------- ---------- ---------- ---------- ---------- ----------
Callithrix_jacchus         ---------- ---------- ---------- ---------- ---------- ----------
Macaca_mulatta             ---------- ---------- ---------- ---------- ---------- ----------
Pongo_pygmaeus             ---------- ---------- ---------- ---------- ---------- ----------
Gorilla_gorilla            ---------- ---------- ---------- ---------- ---------- ----------
Homo_sapiens               ---------- ---------- ---------- ---------- ---------- ----------
Pan_troglodytes            ---------- ---------- ---------- ---------- ---------- ----------
Ochotona_princeps          ---------- ---------- ---------- ---------- ---------- ----------
Oryctolagus_cuniculus      ---------- ---------- ---------- ---------- ---------- ----------
Marmota_monax              ---------- ---------- ---------- ---------- ---------- ----------
Aplodantia_rufa            ---------- ---------- ---------- ---------- ---------- ----------
Spermophilus               ---------- ---------- ---------- ---------- ---------- ----------
Anomamorus                 ---------- ---------- ---------- ---------- ---------- ----------
Maxomys                    ---------- ---------- ---------- ---------- ---------- ----------
Rattus rattus              ---------- ---------- ---------- ---------- ---------- ----------
Rattus exulans             ---------- ---------- ---------- ---------- ---------- ----------
Rattus_norvegicus          ---------- ---------- ---------- ---------- ---------- ----------
Mus_musculus               ---------- ---------- ---------- ---------- ---------- ----------
Mus_spretus                ---------- ---------- ---------- ---------- ---------- ----------
Mus_pahari                 ---------- ---------- ---------- ---------- ---------- ----------
Praomys                    ---------- ---------- ---------- ---------- ---------- ----------
Meriones                   ---------- ---------- ---------- ---------- ---------- ----------
Meriones_crassus           ---------- ---------- ---------- ---------- ---------- ----------
Acomys cahirinus           ---------- ---------- ---------- ---------- ---------- ----------
Lophuromys sikapusi        ---------- ---------- ---------- ---------- ---------- ----------
Mesocricetus_auratus       ---------- ---------- ---------- ---------- ---------- ----------
Oryzomys                   ---------- ---------- ---------- ---------- ---------- ----------
Trichys_fasciculata        ---------- ---------- ---------- ---------- ---------- ----------
Heterocephalus_glaber      ---------- ---------- ---------- ---------- ---------- ----------
Coendou_melanurus          ---------- ---------- ---------- ---------- ---------- ----------
Cavia_porcellus            ---------- ---------- ---------- ---------- ---------- ----------
Ctenomys_maulinus          ---------- ---------- ---------- ---------- ---------- ----------
Octodon_degus              ---------- ---------- ---------- ---------- ---------- ----------
Loxodonta_africana         ---------- ---------- ---------- ---------- ---------- ----------
Procavia_capensis          ---------- ---------- ---------- ---------- ---------- ----------
Echinops_telfairi          ---------- ---------- ---------- ---------- ---------- ----------
Micropotamogale            ---------- ---------- ---------- ---------- ---------- ----------
Myotis_lucifugus           NNNNNNNNNN NNNNNNNNNN NNNNNNNNNN NNNNNNNNNN NNNNNNGCCT TCCGCCCCCC
Canis_familiaris           ---------- ---------- ---------- ---------- ---------- ----------
Felis_catus                ---------- ---------- ---------- ---------- ---------- ----------
Ovis_aries                 ---------- ---------- ---------- ---------- ---------- ----------
Equus_caballus             ---------- ---------- ---------- ---------- ---------- ----------
Tapirus_terrestris         ---------- ---------- ---------- ---------- ---------- ----------
Vicugna                    ---------- ---------- ---------- ---------- ---------- ----------
Sus_scrofa                 ---------- ---------- ---------- ---------- ---------- ----------
Bos_taurus                 ---------- ---------- ---------- ---------- ---------- ----------
Tursiops_truncatus         ---------- ---------- ---------- ---------- ---------- ----------
Erinaceus                  ---------- ---------- ---------- ---------- ---------- ----------
Sorex_araneus
Neomys_anomalus            ---------- ---------- ---------- ---------- ---------- ----------
Choloepus_hoffmanni        ---------- ---------- ---------- ---------- ---------- ----------
Dasypus                    ---------- ---------- ---------- ---------- ---------- ----------
```

```
                        1741
Monodelphis             ---------- ---------- ----CTATTA CA-GTCCT-- -------CTA AA---CTTCT
Macropus                ---------- ---------- ----ATATTA CA-ATCCC-- ------GCCA AA---CTTCT
Microcebus_murinus      ---------- ---------- ----TTCGCC AC-CATGC-- ------CCGG CT-AATTTTT
Tarsius_syrichta        ---------- ---------- ----TTATTC AA-AGCCC-- ------TTCA AA-GCTTCCT
Callithrix_jacchus      ---------- ---------- ----TTATTT AG-AGCTC-- ------TTCA AA-GATTCCT
Macaca_mulatta          ---------- ---------- --------TT AG-AGCTC-- ------TTCA AA-GATTCCT
Pongo_pygmaeus          ---------- ---------- ----TTATTT AG-AGCTC-- ------TTCA AA-GATTCCT
Gorilla_gorilla         ---------- ---------- ---------- ---------- ---------- ----------
Homo_sapiens            ---------- ---------- ----TTATTT AG-AGCTC-- ------TTCA AA-GATTCCT
Pan_troglodytes         ---------- ---------- ----TTATTT AG-AGCTC-- ------TTCA AA-GATTCCT
Ochotona_princeps       ---------- ---------- ----TTGTTA AA--ATTC-- ------TTGA GA-GATTCCC
Oryctolagus_cuniculus   ---------- ---------- ----TGATTT AA-AACTC-- ------TTTA GA-GATTCCT
Marmota_monax           ---------- ---------- ----TTATTT A--------- ---------- ----ATACCT
Aplodantia_rufa         ---------- ---------- ----TTATTT AA-TATCC-- ------TTCG AC-GGTTACT
Spermophilus            ---------- ---------- ----NNNNNN N--------- ---------- ----NNNNNN
Anomamorus              ---------- ---------- ----TTGTTT AW-TACCC-- ------TTCC ----------
Maxomys                 ---------- ---------- ----TTATGT GC-TTCCT-- ------TTCA GA-GATTCCT
Rattus rattus           ---------- ---------- ----TTATGT GC-TTCCT-- ------TTCA GA-GATTCCT
Rattus exulans          ---------- ---------- ----TTATGT GC-TTCCT-- ------TTCA GA-GATTCCT
Rattus_norvegicus       ---------- ---------- ----TTATGT GC-TTCCT-- ------TTCA GA-GATTCCT
Mus_musculus            ---------- ---------- ----TTATGT GC-TTCCT-- ------TTCA GA-GATTCCT
Mus_spretus             ---------- ---------- ----TTATGT GC-TTCCT-- ------TT-- -----TTCCT
Mus_pahari              ---------- ---------- ----TTTTGT GC-ATCCT-- ------TTCA GA-GATTCCT
Praomys                 ---------- ---------- ----TTATGT GC-TTCCT-- ------CTCA GA-GATTCCT
Meriones                ---------- ---------- ----TTATGT GA-GACCT-- ------TTCA GA-GATTCCT
Meriones_crassus        ---------- ---------- ----TTA--- ---------- ---------- ----------
Acomys cahirinus        ---------- ---------- ---------- ---------- ---------- ----------
Lophuromys sikapusi     ---------- ---------- ---------- ---------- ---------- ----------
Mesocricetus_auratus    ---------- ---------- ----TTATGT GA-TACCT-- ------TTCA GA-TATTCCT
Oryzomys                ---------- ---------- ----TTGTGT GA-TACCT-- ------TTCA GA-GAGTCCT
Trichys_fasciculata     ---------- ---------- ----TTATCT GA-TATGC-- ------TTCA TA-GAATGAT
Heterocephalus_glaber   ---------- ---------- ----TTACTG GA-TACAC-- ------TTCA GA-GATTCAT
Coendou_melanurus       ---------- ---------- ----GTACTG CA-TACAC-- ------TTCA GA-GATTCAT
Cavia_porcellus         ---------- ---------- ----GTACTT GA-TACAT-- ------TTCA GA-GATTCAT
Ctenomys_maulinus       ---------- ---------- ----GTACTG AA-TACAC-- ------TTCA GA-GATTCAT
Octodon_degus           ---------- ---------- ----GTACTG GA-TACAC-- ------TTTA GA-GATTCAT
Loxodonta_africana      ---------- ---------- ----TTATTT AAAAACAT-- ------TTCA AA-GATTCCC
Procavia_capensis       ---------- ---------- ----TTATTT AAGAAC---- ---------- ----------
Echinops_telfairi       ---------- ---------- ---GTTGTTC TGGAGCATT- ---------- ----------
Micropotamogale         ---------- ---------- ---AACACTT AGG------- ---------- ----------
Myotis_lucifugus        TCCGCCCCCA CCTCCCCAGC GCTTTTGTTT GCGCTCCTGC GCCAGATTGA GACAAGTCCT
Canis_familiaris        ---------- ---------- ----TTATTC AA-AACCC-- ------TGAA GA-GAGTGCT
Felis_catus             ---------- ---------- ----TTCTTT AAGAACCC-- ------TTAG GA-GAGTCCT
Ovis_aries              ---------- ---------- ---------- ---------- ---------- ----------
Equus_caballus          ---------- ---------- ---------- ---------- ---------- -------CCT
Tapirus_terrestris      ---------- ---------- ----TTATTT CA-AACCC-- ------TGAA GA-GATTCCT
Vicugna                 ---------- ---------- ----TTATTT AAAAACCT-- ------TTAA G-----TCCT
Sus_scrofa              ---------- ---------- ----TTACTT AA-AACCC-- ------TTAA GA-GAGTCCT
Bos_taurus              ---------- ---------- ----TTATTT TA-AACCC-- ------TTAA GA-GATTCCT
Tursiops_truncatus      ---------- ---------- ----TTATTT TA-AACCC-- ------TTAA GA-GATTACT
Erinaceus               ---------- ---------- ----TTATTT AGAATCCT-- ------CTTA GAGGATTCCT
Sorex_araneus
Neomys_anomalus         ---------- ---------- ----CTCACC TAAGCCCT-- --------TA AGGAATTCCT
Choloepus_hoffmanni     ---------- ---------- ----TTTCTC AA-AACCC-- ------TTCT AA-GCTTTCC
Dasypus                 ---------- ---------- ----TTCATT AA-AACCC-- ------TTCT CA-GTTTTCT
```

```
                          1801
Monodelphis               GCTTACTTCA ---------- ---------- ---------- ---------- ----------
Macropus                  ACTTATTTCA ---------- ---------- ---------- ---------- ----------
Microcebus_murinus        TGTATATATA TTAGTTGCTC AATTAATT-- ---------- ---------- ----------
Tarsius_syrichta          ATGATATTTA ---------- ----AATT-- ---------- ---------- ----------
Callithrix_jacchus        ACTAAGTTTA ---------- ----AAAT-- ---------- ---------- ----------
Macaca_mulatta            ATTTAATTTA ---------- ----AAAT-- ---------- ---------- ----------
Pongo_pygmaeus            ATTTAATTTA ---------- ----AAAT-- ---------- ---------- ----------
Gorilla_gorilla           ---------- ---------- ---------- ---------- ---------- ----------
Homo_sapiens              ATTTAATTTA ---------- ----AAAT-- ---------- ---------- ----------
Pan_troglodytes           ATTTAATTTA ---------- ----AAAT-- ---------- ---------- ----------
Ochotona_princeps         ATTAAATTTG ---------- ----AATT-- ---------- ---------- ----------
Oryctolagus_cuniculus     AGTAAATTTA ---------- ----AATT-- ---------- ---------- ----------
Marmota_monax             ATTAAATTTA ---------- ----AATT-- ---------- ---------- ----------
Aplodantia_rufa           ATTAAATTCA ---------- ----AATT-- ---------- ---------- ----------
Spermophilus              NNNNNNNNNN ---------- ----NNNN-- ---------- ---------- ----------
Anomamorus                ---------- ---------- ---------- ---------- ---------- ----------
Maxomys                   ATTAAATTTA ---------- ----ACTT-- ---------- ---------- ----------
Rattus rattus             ATTAAATTT- ---------- ---------- ---------- ---------- ----------
Rattus exulans            ATTAAATTT- ---------- ---------- ---------- ---------- ----------
Rattus_norvegicus         ATTAAATTT- ---------- ---------- ---------- ---------- ----------
Mus_musculus              ATTAAATGTA ---------- ----ACGT-- ---------- ---------- ----------
Mus_spretus               TT-------- ---------- ---------- ---------- ---------- ----------
Mus_pahari                ATTAAATGTA ---------- ----ACTT-- ---------- ---------- ----------
Praomys                   ATTAAATGTA ---------- ----ACTT-- ---------- ---------- ----------
Meriones                  ATTAAATTTA ---------- ----AGTT-- ---------- ---------- ----------
Meriones_crassus          ---------- ---------- ---------- ---------- ---------- ----------
Acomys cahirinus          ---------- ---------- ---------- ---------- ---------- ----------
Lophuromys sikapusi       ---------- ---------- ---------- ---------- ---------- ----------
Mesocricetus_auratus      ATTAAATTTA ---------- ----AATT-- ---------- ---------- ----------
Oryzomys                  ATTAAACTTA ---------- ----AATT-- ---------- ---------- ----------
Trichys_fasciculata       ATTAAATTTA ---------- ----AATT-- ---------- ---------- ----------
Heterocephalus_glaber     ATTAAATTTA ---------- ----AATT-- ---------- ---------- ----------
Coendou_melanurus         ATTAAATTTA ---------- ----AATT-- ---------- ---------- ----------
Cavia_porcellus           ATTGAATTTA ---------- ----AATT-- ---------- ---------- ----------
Ctenomys_maulinus         ATT--ATTTA ---------- ----AATT-- ---------- ---------- ----------
Octodon_degus             ATT--ATTTA ---------- ----AATT-- ---------- ---------- ----------
Loxodonta_africana        ATTAAATT-- ---------- ---------- ---------- ---------- ----------
Procavia_capensis         ---------- ---------- ---------- ---------- ---------- ----------
Echinops_telfairi         ---------- ---------- ---------- ---------- ---------- ----------
Micropotamogale           ---------- ---------- ---------- ---------- ---------- ----------
Myotis_lucifugus          CCCACCTCTA GTGAAGAAGA AGGTGGATTC CCCTGCGCCT CG-------- ----------
Canis_familiaris          ATTAAATTCA ---------- ----AACT-- ---------- ---------- ----------
Felis_catus               GTTACATTTA ---------- ----AATT-- ---------- ---------- ----------
Ovis_aries                ---------- ---------- ---------- ---------- ---------- ----------
Equus_caballus            ATTAAATTTC ---------- ----ACTC-- ---------- ---------- ----------
Tapirus_terrestris        ATTCAATTTA ---------- ----ACCT-- ---------- ---------- ----------
Vicugna                   ATTACATTTA ---------- ----AATT-- ---------- ---------- ----------
Sus_scrofa                ATTGAATTTA ---------- ----AATT-- ---------- ---------- ----------
Bos_taurus                ------TTTA ---------- ----AATT-- ---------- ---------- ----------
Tursiops_truncatus        ATTAAATTTA ---------- ----AATT-- ---------- ---------- ----------
Erinaceus                 ATGACATTCA ---------- ----AATT-- ---------- ---------- ----------
Sorex_araneus
Neomys_anomalus           ATTACGTTTA ---------- ----AATT-- ---------- ---------- ----------
Choloepus_hoffmanni       ATTAAATTTA ---------- ----AATT-- ---------- ---------- ----------
Dasypus                   GTTACATTTA ---------- ----AAGT-- ---------- ---------- ----------
```

```
                    1861
Monodelphis             ---------- ---------- ---------- ---------- ---------- ----------
Macropus                ---------- ---------- ---------- ---------- ---------- ----------
Microcebus_murinus      ---------- ---------- ---------- ---------- ---------- ----------
Tarsius_syrichta        ---------- ---------- ---------- ---------- ---------- ----------
Callithrix_jacchus      ---------- ---------- ---------- ---------- ---------- ----------
Macaca_mulatta          ---------- ---------- ---------- ---------- ---------- ----------
Pongo_pygmaeus          ---------- ---------- ---------- ---------- ---------- ----------
Gorilla_gorilla         ---------- ---------- ---------- ---------- ---------- ----------
Homo_sapiens            ---------- ---------- ---------- ---------- ---------- ----------
Pan_troglodytes         ---------- ---------- ---------- ---------- ---------- ----------
Ochotona_princeps       ---------- ---------- ---------- ---------- ---------- ----------
Oryctolagus_cuniculus   ---------- ---------- ---------- ---------- ---------- ----------
Marmota_monax           ---------- ---------- ---------- ---------- ---------- ----------
Aplodantia_rufa         ---------- ---------- ---------- ---------- ---------- ----------
Spermophilus            ---------- ---------- ---------- ---------- ---------- ----------
Anomamorus              ---------- ---------- ---------- ---------- ---------- ----------
Maxomys                 ---------- ---------- ---------- ---------- ---------- ----------
Rattus rattus           ---------- ---------- ---------- ---------- ---------- ----------
Rattus exulans          ---------- ---------- ---------- ---------- ---------- ----------
Rattus_norvegicus       ---------- ---------- ---------- ---------- ---------- ----------
Mus_musculus            ---------- ---------- ---------- ---------- ---------- ----------
Mus_spretus             ---------- ---------- ---------- ---------- ---------- ----------
Mus_pahari              ---------- ---------- ---------- ---------- ---------- ----------
Praomys                 ---------- ---------- ---------- ---------- ---------- ----------
Meriones                ---------- ---------- ---------- ---------- ---------- ----------
Meriones_crassus        ---------- ---------- ---------- ---------- ---------- ----------
Acomys cahirinus        ---------- ---------- ---------- ---------- ---------- ----------
Lophuromys sikapusi     ---------- ---------- ---------- ---------- ---------- ----------
Mesocricetus_auratus    ---------- ---------- ---------- ---------- ---------- ----------
Oryzomys                ---------- ---------- ---------- ---------- ---------- ----------
Trichys_fasciculata     ---------- ---------- ---------- ---------- ---------- ----------
Heterocephalus_glaber   ---------- ---------- ---------- ---------- ---------- ----------
Coendou_melanurus       ---------- ---------- ---------- ---------- ---------- ----------
Cavia_porcellus         ---------- ---------- ---------- ---------- ---------- ----------
Ctenomys_maulinus       ---------- ---------- ---------- ---------- ---------- ----------
Octodon_degus           ---------- ---------- ---------- ---------- ---------- ----------
Loxodonta_africana      ---------- ---------- ---------- ---------- ---------- ----------
Procavia_capensis       ---------- ---------- ---------- ---------- ---------- ----------
Echinops_telfairi       ---------- ---------- ---------- ---------- ---------- ----------
Micropotamogale         ---------- ---------- ---------- ---------- ---------- ----------
Myotis_lucifugus        ---------- ---------- ---------- ---------- ---------- ----------
Canis_familiaris        ---------- ---------- ---------- ---------- ---------- ----------
Felis_catus             ---------- ---------- ---------- ---------- ---------- ----------
Ovis_aries              ---------- ---------- ---------- ---------- ---------- ----------
Equus_caballus          ---------- ---------- ---------- ---------- ---------- ----------
Tapirus_terrestris      ---------- ---------- ---------- ---------- ---------- ----------
Vicugna                 ---------- ---------- ---------- ---------- ---------- ----------
Sus_scrofa              ---------- ---------- ---------- ---------- ---------- ----------
Bos_taurus              ---------- ---------- ---------- ---------- ---------- ----------
Tursiops_truncatus      ---------- ---------- ---------- ---------- ---------- ----------
Erinaceus               ---------- ---------- ---------- ---------- ---------- ----------
Sorex_araneus
Neomys_anomalus         ---------- ---------- ---------- ---------- ---------- ----------
Choloepus_hoffmanni     ---------- ---------- ---------- ---------- ---------- ----------
Dasypus                 ---------- ---------- ---------- ---------- ---------- ----------
```

```
                    1921
Monodelphis            ---------- ---------- ---------- ---------- ---------- ----------
Macropus               ---------- ---------- ---------- ---------- ---------- ----------
Microcebus_murinus     ---------- ---------- ---------- ---------- ---------- ----------
Tarsius_syrichta       ---------- ---------- ---------- ---------- ---------- ----------
Callithrix_jacchus     ---------- ---------- ---------- ---------- ---------- ----------
Macaca_mulatta         ---------- ---------- ---------- ---------- ---------- ----------
Pongo_pygmaeus         ---------- ---------- ---------- ---------- ---------- ----------
Gorilla_gorilla        ---------- ---------- ---------- ---------- ---------- ----------
Homo_sapiens           ---------- ---------- ---------- ---------- ---------- ----------
Pan_troglodytes        ---------- ---------- ---------- ---------- ---------- ----------
Ochotona_princeps      ---------- ---------- ---------- ---------- ---------- ----------
Oryctolagus_cuniculus  ---------- ---------- ---------- ---------- ---------- ----------
Marmota_monax          ---------- ---------- ---------- ---------- ---------- ----------
Aplodantia_rufa        ---------- ---------- ---------- ---------- ---------- ----------
Spermophilus           ---------- ---------- ---------- ---------- ---------- ----------
Anomamorus             ---------- ---------- ---------- ---------- ---------- ----------
Maxomys                ---------- ---------- ---------- ---------- ---------- ----------
Rattus rattus          ---------- ---------- ---------- ---------- ---------- ----------
Rattus exulans         ---------- ---------- ---------- ---------- ---------- ----------
Rattus_norvegicus      ---------- ---------- ---------- ---------- ---------- ----------
Mus_musculus           ---------- ---------- ---------- ---------- ---------- ----------
Mus_spretus            ---------- ---------- ---------- ---------- ---------- ----------
Mus_pahari             ---------- ---------- ---------- ---------- ---------- ----------
Praomys                ---------- ---------- ---------- ---------- ---------- ----------
Meriones               ---------- ---------- ---------- ---------- ---------- ----------
Meriones_crassus       ---------- ---------- ---------- ---------- ---------- ----------
Acomys cahirinus       ---------- ---------- ---------- ---------- ---------- ----------
Lophuromys sikapusi    ---------- ---------- ---------- ---------- ---------- ----------
Mesocricetus_auratus   ---------- ---------- ---------- ---------- ---------- ----------
Oryzomys               ---------- ---------- ---------- ---------- ---------- ----------
Trichys_fasciculata    ---------- ---------- ---------- ---------- ---------- ----------
Heterocephalus_glaber  ---------- ---------- ---------- ---------- ---------- ----------
Coendou_melanurus      ---------- ---------- ---------- ---------- ---------- ----------
Cavia_porcellus        ---------- ---------- ---------- ---------- ---------- ----------
Ctenomys_maulinus      ---------- ---------- ---------- ---------- ---------- ----------
Octodon_degus          ---------- ---------- ---------- ---------- ---------- ----------
Loxodonta_africana     ---------- ---------- ---------- ---------- ---------- ----------
Procavia_capensis      ---------- ---------- ---------- ---------- ---------- ----------
Echinops_telfairi      ---------- ---------- ---------- ---------- ---------- ----------
Micropotamogale        ---------- ---------- ---------- ---------- ---------- ----------
Myotis_lucifugus       ---------- ---------- ---------- ---------- ---------- ----------
Canis_familiaris       ---------- ---------- ---------- ---------- ---------- ----------
Felis_catus            ---------- ---------- ---------- ---------- ---------- ----------
Ovis_aries             ---------- ---------- ---------- ---------- ---------- ----------
Equus_caballus         ---------- ---------- ---------- ---------- ---------- ----------
Tapirus_terrestris     ---------- ---------- ---------- ---------- ---------- ----------
Vicugna                ---------- ---------- ---------- ---------- ---------- ----------
Sus_scrofa             ---------- ---------- ---------- ---------- ---------- ----------
Bos_taurus             ---------- ---------- ---------- ---------- ---------- ----------
Tursiops_truncatus     ---------- ---------- ---------- ---------- ---------- ----------
Erinaceus              ---------- ---------- ---------- ---------- ---------- ----------
Sorex_araneus
Neomys_anomalus        ---------- ---------- ---------- ---------- ---------- ----------
Choloepus_hoffmanni    ---------- ---------- ---------- ---------- ---------- ----------
Dasypus                ---------- ---------- ---------- ---------- ---------- ----------
```

```
                         1981
Monodelphis              ---------A TGTCATCACA T--------- ---------- ---------- ----------
Macropus                 ---------A TGTCACCACA TAT------- ---------- ---------- ----------
Microcebus_murinus       -------TCT TTCTATTTTT ATA------- ---------- ---------- ----------
Tarsius_syrichta         -------GCC TGTAACCTGC ---------- ---------- ---------- ----------
Callithrix_jacchus       -------GCC CGTAGCCTTC ---------- ---------- ---------- ----------
Macaca_mulatta           -------GCC CGTCGCCTTC ---------- ---------- ---------- ----------
Pongo_pygmaeus           -------GCC TGTCGCCTTC ---------- ---------- ---------- ----------
Gorilla_gorilla          ---------- ---------- ---------- ---------- ---------- ----------
Homo_sapiens             -------GCC TGTCGCCTTC ---------- ---------- ---------- ----------
Pan_troglodytes          -------GCC TGTCGCCTTC ---------- ---------- ---------- ----------
Ochotona_princeps        -------GCA TGTAACATT- ---------- ---------- ---------- ----------
Oryctolagus_cuniculus    -------GCC TGTAACATTC ---------- ---------- ---------- ----------
Marmota_monax            -------GCC TGTAACCTTC ---------- ---------- ---------- ----------
Aplodantia_rufa          -------GCC TGCAGCCTTA GGA------- ---------- ---------- ----------
Spermophilus             -------NNN NNNNNNNNNN ---------- ---------- ---------- ----------
Anomamorus               ---------- ---------- ---------- ---------- ---------- ----------
Maxomys                  -------GTC TGTAACCTTT GTAAATGGCA GAT------A GTGAAGATAC AGCCACTAGA
Rattus rattus            -------GTC TGTAACCTTT GTAAATGGCA GAT------A GTGAAAATAC AGCCACTAGA
Rattus exulans           -------GTC TGTAACCTTT GTAAATGGCA GAT------A GTGAAAATAC AGCCACTAGA
Rattus_norvegicus        -------GTC TGTAACCTTT GTAAATGGCA GAT------A GTGAAAATAC AGCCACTAGA
Mus_musculus             -------GTC TGTAACCTTT GTATATGGCA CAT------A GTGAAGATGC TGCCACTAGA
Mus_spretus              ---------- ---------- ---------- ---------- ---------- ----------
Mus_pahari               -------GTC TGTAACCTTT GTATATGGCA GAT------A GTGAAGAGAA GGCCACTAGA
Praomys                  -------GTC TGTAACCTTT GTGTAAGGCA GAT------T GTGAAAACAC AGCTACTGGA
Meriones                 -------GTC TGTGACCTTT TGTATAGGGC ATA-CAGTGA ATGAAAATTC AGCCATTAGA
Meriones_crassus         ---------- ---------- ---------- ---------- ---------- ----------
Acomys cahirinus         ---------- ---------- ---------- ---------- ---------- ---CACTAGA
Lophuromys sikapusi      ---------- ---------- ---------- ---------- ---------- ---CACTAGA
Mesocricetus_auratus     -------GTC TGTAACCTTT GTACATGGCA GATACAGTTA GTGAAAA-AC CGCCACTAAA
Oryzomys                 -------GTC TGTAACCTTT GTATACGGCA GAAACAGTCA GTGGGAGTAT AGCCATTAGA
Trichys_fasciculata      -------ACA TGTGACCTTC GTA------- ---------- ---------- ----------
Heterocephalus_glaber    -------ACC TCTGACCTTC ACA------- ---------- ---------- ----------
Coendou_melanurus        -------ACC TGTGACCTTC ATA------- ---------- ---------- ----------
Cavia_porcellus          -------ACC TGTGACCTTC ATA------- ---------- ---------- ----------
Ctenomys_maulinus        -------ACC TGTGACCTTC ATA------- ---------- ---------- ----------
Octodon_degus            -------ACC TGTGGCCTTC ATA------- ---------- ---------- ----------
Loxodonta_africana       -------GCC TGTAACCCTT ---------- ---------- ---------- ----------
Procavia_capensis        --------CC TGCAGCCCTT ---------- ---------- ---------- ----------
Echinops_telfairi        ------CTCC TACTCACC-- ---------- ---------- ---------- ----------
Micropotamogale          -----TTACC TACAGGCCTT ---------- ---------- ---------- ----------
Myotis_lucifugus         -------GCC TCTCCCTTCC TCC------- ---------- ---------- ----------
Canis_familiaris         -------GCC TGTAACCTTT ---------- ---------- ---------- ----------
Felis_catus              -------GCC TATAACGTTT ---------- ---------- ---------- ----------
Ovis_aries               ---------- ---------- ---------- ---------- ---------- ----------
Equus_caballus           -------GCC TGTAACCTTT ---------- ---------- ---------- ----------
Tapirus_terrestris       -------GCC TGTAACMTTT ---------- ---------- ---------- ----------
Vicugna                  -------GCC TGTAACCTTT GTA------- ---------- ---------- ----------
Sus_scrofa               -------GCC TCTCATTTTT GTA------- ---------- ---------- ----------
Bos_taurus               -------ACC TGTAACCACT GTA------- ---------- ---------- ----------
Tursiops_truncatus       -------GCC TGTAACCCTC GTA------- ---------- ---------- ----------
Erinaceus                -------GTC TGTAACTTTG ---------- ---------- ---------- ----------
Sorex_araneus
Neomys_anomalus          -------GCT TGTGTTTGCT ---------- ---------- ---------- ----------
Choloepus_hoffmanni      -------GCC TGTAACTGTT ---------- ---------- ---------- ----------
Dasypus                  -------GCC TGTAGCCTTT ---------- ---------- ---------- ----------
```

```
                          2041
Monodelphis               ---GTGTGGA AGC------- ---TTTGTGA TTAATAGTGG GTTTT----- ----------
Macropus                  ---GTGTGGA AGC------- ---TATGTGA TTAATAATGG CTTTT----- ----------
Microcebus_murinus        ----GTAGAG ACGG------ ----GGTCTC GCTCTTGCTC AGGCTG---- ----------
Tarsius_syrichta          ----ACATAA TAGA------ ----CTCAGG ACTGATGATA GGTTTA---- ----------
Callithrix_jacchus        ----CTATAA TAGC------ ----TTCAAG GTTGATGATA GCTTTA---- ----------
Macaca_mulatta            ----CTATAA TAGA------ ----CTTATG ATGGATGATA GTTTTA---- ----------
Pongo_pygmaeus            ----CTATAA TAGA------ ----CTTATG ATGGATGATA GCTTTA---- ----------
Gorilla_gorilla           ---------- ---------- ---------- ---------- ---------- ----------
Homo_sapiens              ----CTATAA TAGG------ ----CTTATG ATGGATGATA GCTTTA---- ----------
Pan_troglodytes           ----CTATAA TAGA------ ----CTTATG ATGGATGATA GCTTTA---- ----------
Ochotona_princeps         ---------- ---------- -------AGG ATTGATGATG GGTTTG---- ----------
Oryctolagus_cuniculus     ----CAATAA TAAA------ ----TTAAGT TTTAATTAAG TGTTTA---- ----------
Marmota_monax             ----CTGGAA TGGA------ ---------- ---CTTGATA AGTGTA---- ----------
Aplodantia_rufa           ----TAATA- --GA------ ---------- ---CTT---A CGTTTA---- ----------
Spermophilus              ----NNNNNN NNNN------ ---------- ---NNNNNNN NNNNNN---- ----------
Anomamorus                ---------- ---------- ---------- ---------- ---------- ----------
Maxomys                   GCTGGAGGGG TGG----CTT AGCATTAAGA GTGC-TATCA CGTTTGCAAA AGACCCAAGT
Rattus rattus             GCTGGAGGGG TGG----CTT AGCATTAAGA GC---TATCG CGTTTGCAAA AGACCCAATT
Rattus exulans            GCTGGAGGGG TGG----CTT AGCATTAAGA GCGC-TATCG CGTTTGCAAA AGACCCAATT
Rattus_norvegicus         GCTGGAGGGG TGG----CTT AGCATTAAGA GCGC-TATCG CGTTTGCAAA AGACCCAATT
Mus_musculus              GCTGGAGAGG CAG----CTT AGCCTTGAA- AGTGCTACCA CTTTTGCAAA AGACCCAAGC
Mus_spretus               ---------- ---------- ---------- ---------- ---------- ----------
Mus_pahari                GCTGGAGAGG CAGGCACCTT AGCCTCGAGA GTGC-CATCA CTTTTGCAAA AGACCCAAGT
Praomys                   GCTGGAGAGG CGG----CTT AGCATTGAGA GTGC-TATCA CTTTTGCAAA AGCCTCAAGT
Meriones                  GCTGGAAACG TGG----CTT AGCATTCAGA GTGC-TATTG CTTTTGCAAA CGACCCAAGT
Meriones_crassus          ---------- ---------- ---------- ---------- ---------- ----------
Acomys cahirinus          GCTGGGGAAG TGG----CTT AGCACTGAGT GGTGCTGTTG CTTTTGCAAA GGAC------
Lophuromys sikapusi       GCTGGGGAAG TGG----CTT AGCACTGAGT GGAGCTGTTG CTTTTGCAAA GGATCC----
Mesocricetus_auratus      GTTGGAGAGG TGG----CTT AACATT--GA GTGCTTATTG TTCTTGCAGA GGACCCAAGT
Oryzomys                  GTTGGAGRGG TGG----CTT AGCCTTTAGA GTGCTTATTG YTCTTGCGGA GGRCCCAGGT
Trichys_fasciculata       ----TAACAC TTA------- ---------G ATTGATGATA CGTTTA---- ----------
Heterocephalus_glaber     ----TAACA- ---------- ----TTTATG CTTGATGATA TAGTTT---- ----------
Coendou_melanurus         ----TAACGA TCGA------ ----TG---- ------ATTA CATTTA---- ----------
Cavia_porcellus           ----TAACGA TTGG------ ---------- ---GTGATTA CATTTA---- ----------
Ctenomys_maulinus         ----TAACGC T--------- ----TG---- ---------- -----A---- ----------
Octodon_degus             ----TAACGA TTGA------ ----TG---- ---------- -----A---- ----------
Loxodonta_africana        -----GTTTC TTAGA----- ----CTTATG ATTGACAATG GGCTTC---- ----------
Procavia_capensis         -----GT--- ---------- ---------- ---------- ---------- ----------
Echinops_telfairi         ---------- --AGA----- -------ATC CT-TCCACTA GGCCAC---- ----------
Micropotamogale           -----GTAA- -TAGA----- -----GCTGA CTGGTAACAG GTCTG----- ----------
Myotis_lucifugus          ----CTCTCT CAGGCCTCCT CGCTTCTCCG CTTCTCACTT CACTTG---- ----------
Canis_familiaris          ----GTGTAA TGGA------ ----TTTCTG ACTGATGATG AGTTTA---- ----------
Felis_catus               ----GTGTAA TAGA------ ----CTGATG ACGGACGATA GGTTTA---- ----------
Ovis_aries                ---------- ---------- ---------- ---------- ---------- ----------
Equus_caballus            ----GTATAA TTTA------ --------TG ATTGATGATA GGTTTA---- ----------
Tapirus_terrestris        ----GTATAA TTTA------ --------TG CTTGATGATA GGTTTA---- ----------
Vicugna                   ----TAATAA TAGA------ ----TT--TG ATTGATGACA AGCTTA---- ----------
Sus_scrofa                ----TAATAA TAGG------ ----TTTATG ACTGATGCTA GGTTTA---- ----------
Bos_taurus                ----TAATAA AAGA------ ----TTTATG ATTGATGCTA AATTTA---- ----------
Tursiops_truncatus        ----TAGTAA TAGA------ ----TTTATG GTGGATGATA GGTTTA---- ----------
Erinaceus                 ---------- ---------- -------TAC AGTGATGATA AGTTGA---- ----------
Sorex_araneus
Neomys_anomalus           ----GCATAA TGAA------ ----TTGATG ACTGATGATA GGTTTA---- ----------
Choloepus_hoffmanni       ----GTATAA TAGA------ ----CCTGTG ACTGGTATTA ---------- ----------
Dasypus                   ----GAAGAA TAGG------ ----TTTCTG ATCGACATTA AGACTA---- ----------
```

```
                        2101
Monodelphis                ---------- ---------- ---------- ---------- ---------- ----------
Macropus                   ---------- ---------- ---------- ---------- ---------- ----------
Microcebus_murinus         ---------- ---------- ---------- ---------- ---------- ----------
Tarsius_syrichta           ---------- ---------- ---------- ---------- ---------- ----------
Callithrix_jacchus         ---------- ---------- ---------- ---------- ---------- ----------
Macaca_mulatta             ---------- ---------- ---------- ---------- ---------- ----------
Pongo_pygmaeus             ---------- ---------- ---------- ---------- ---------- ----------
Gorilla_gorilla            ---------- ---------- ---------- ---------- ---------- ----------
Homo_sapiens               ---------- ---------- ---------- ---------- ---------- ----------
Pan_troglodytes            ---------- ---------- ---------- ---------- ---------- ----------
Ochotona_princeps          ---------- ---------- ---------- ---------- ---------- ----------
Oryctolagus_cuniculus      ---------- ---------- ---------- ---------- ---------- ----------
Marmota_monax              ---------- ---------- ---------- ---------- ---------- ----------
Aplodantia_rufa            ---------- ---------- ---------- ---------- ---------- ----------
Spermophilus               ---------- ---------- ---------- ---------- ---------- ----------
Anomamorus                 ---------- ---------- ---------- ---------- ---------- ----------
Maxomys                    CCAGTTCCTA GCATGGCCAC AAACAGCAGT AACTCTAGCT TTAGGGGGGC CACCACCCTC
Rattus rattus              TCAGTTCCTA GCATAGCCAC AACCACCAGC AACTCCAGCT TTAGGGGAGC CACCACTTTC
Rattus exulans             TCAGTTCCTA GCATGGCCAC AACCACCAGC AACTCCAGCT TTAGGGGAGC CACCACTTTC
Rattus_norvegicus          TCAGTTCCTA GCATGGCCAC AACCACCAGC AACTCCAGCT TTAGGGGAGC CACCACCTTC
Mus_musculus               TCAGTTCCTA GCAAGGCCAC AACCACCAGC AACTCCACCT TTAGGGGAGC TACCACCGTC
Mus_spretus                ---------- ---------- ---------- ---------- ---------- ----------
Mus_pahari                 TCAGTTCCTA GCAAGGCCAC AACCACCAGC AACTCCAGCT TCGGGGGAGC TATCACCTTY
Praomys                    TCAATTCCTA GCAAGGCCAC AACCACCAGC AACTTCAGCT TTCGGGGAGC TACAACCCTC
Meriones                   TCAGTTCCTA GCATGGCCAC AAACACCCAC AACTCCAGCT CTAGGGGACC CAACGCCCTA
Meriones_crassus           ---------- ---------- ---------- ---------- ---------- ----------
Acomys cahirinus           ---------- ---------- ---------- ---------- ---------- ----------
Lophuromys sikapusi        ---------- ---------- ---------- ---------- ---------- ----------
Mesocricetus_auratus       TCAGGTCTTA GC-------- ---------- AACTCCAACT CCAGGGGATC CAACACCCTC
Oryzomys                   TCGGTTCCTA GCATGGCCGC AAGCACCTGC AACTCCAACT CCAGGGAACC CAACGCCCTC
Trichys_fasciculata        ---------- ---------- ---------- ---------- ---------- ----------
Heterocephalus_glaber      ---------- ---------- ---------- ---------- ---------- ----------
Coendou_melanurus          ---------- ---------- ---------- ---------- ---------- ----------
Cavia_porcellus            ---------- ---------- ---------- ---------- ---------- ----------
Ctenomys_maulinus          ---------- ---------- ---------- ---------- ---------- ----------
Octodon_degus              ---------- ---------- ---------- ---------- ---------- ----------
Loxodonta_africana         ---------- ---------- ---------- ---------- ---------- ----------
Procavia_capensis          ---------- ---------- ---------- ---------- ---------- ----------
Echinops_telfairi          ---------- ---------- ---------- ---------- ---------- ----------
Micropotamogale            ---------- ---------- ---------- ---------- ---------- ----------
Myotis_lucifugus           ---------- ---------- ---------- ---------- ---------- ----------
Canis_familiaris           ---------- ---------- ---------- ---------- ---------- ----------
Felis_catus                ---------- ---------- ---------- ---------- ---------- ----------
Ovis_aries                 ---------- ---------- ---------- ---------- ---------- ----------
Equus_caballus             ---------- ---------- ---------- ---------- ---------- ----------
Tapirus_terrestris         ---------- ---------- ---------- ---------- ---------- ----------
Vicugna                    ---------- ---------- ---------- ---------- ---------- ----------
Sus_scrofa                 ---------- ---------- ---------- ---------- ---------- ----------
Bos_taurus                 ---------- ---------- ---------- ---------- ---------- ----------
Tursiops_truncatus         ---------- ---------- ---------- ---------- ---------- ----------
Erinaceus                  ---------- ---------- ---------- ---------- ---------- ----------
Sorex_araneus
Neomys_anomalus            ---------- ---------- ---------- ---------- ---------- ----------
Choloepus_hoffmanni        ---------- ---------- ---------- ---------- ---------- ----------
Dasypus                    ---------- ---------- ---------- ---------- ---------- ----------
```

```
                    2161
Monodelphis               ---------- ---------- ---------- ---------- ---------- ----------
Macropus                  ---------- ---------- ---------- ---------- ---------- ----------
Microcebus_murinus        ---------- ---------- ---------- ---------- ---------- ----------
Tarsius_syrichta          ---------- ---------- ---------- ---------- ---------- ----------
Callithrix_jacchus        ---------- ---------- ---------- ---------- ---------- ----------
Macaca_mulatta            ---------- ---------- ---------- ---------- ---------- ----------
Pongo_pygmaeus            ---------- ---------- ---------- ---------- ---------- ----------
Gorilla_gorilla           ---------- ---------- ---------- ---------- ---------- ----------
Homo_sapiens              ---------- ---------- ---------- ---------- ---------- ----------
Pan_troglodytes           ---------- ---------- ---------- ---------- ---------- ----------
Ochotona_princeps         ---------- ---------- ---------- ---------- ---------- ----------
Oryctolagus_cuniculus     ---------- ---------- ---------- ---------- ---------- ----------
Marmota_monax             ---------- ---------- ---------- ---------- ---------- ----------
Aplodantia_rufa           ---------- ---------- ---------- ---------- ---------- ----------
Spermophilus              ---------- ---------- ---------- ---------- ---------- ----------
Anomamorus                ---------- ---------- ---------- ---------- ---------- ----------
Maxomys                   TTCTAGATTC TGTAGGCACT ATACTCATGC GCTCGCACCC TATCCCCCCA -TATAATTT-
Rattus rattus             TTCTTGATTC TGTAGGCACT GTACTCATGT GCTCACACCC CATCCCCCAA -TACGATTT-
Rattus exulans            TTCTTGATTC TGTAGGCACT GTACTCATGT GCTCACACCC CATCCCCCGA -TACGATTT-
Rattus_norvegicus         TTCTTGACTC TGTAGGCACT GTACTCATGT GCTCACACCC CATCCCCCGA -TACGATTT-
Mus_musculus              TTCTAGACCC TGTAGGCACT GTACTTGTGT GTTCATACCC CTCACCCCCA ATATGATTTA
Mus_spretus               ---------- ---------- ---------- ---------- ---------- ----------
Mus_pahari                TTYTAGATTC YGTAGGCACT GTACTC-TGT GTACATACCC ----TCCCCA -TATGATTT-
Praomys                   TTCTAGACTC TGTAGGCACT GTACTCRTGT GCACATATCC CATGCCCCCA -TGTAACTT-
Meriones                  TTCTGGACTC TGTAGGCACT GCATTCATGT ATGCATAACC --CACCCCAC ATATGATTTT
Meriones_crassus          ---------- ---------- ---------- ---------- ---------- ----------
Acomys cahirinus          ---------- ---------- ---------- ---------- --CACTCCAC AT--GATTT-
Lophuromys sikapusi       ---------- ---------- ---------- ---------- --CACCCCGC AT--GATTT-
Mesocricetus_auratus      TTCTGGACTC CGTAGGCACT GCGTTCACGT GTAAATAAC- ----GCCCCA -TGTGGTTT-
Oryzomys                  TTCTGGATTC TGTAGGCACT GCATTTACAT GAACACAAC- ---TTCCCCA -CGTAGTTT-
Trichys_fasciculata       ---------- ---------- ---------- ---------- ---------- ----------
Heterocephalus_glaber     ---------- ---------- ---------- ---------- ---------- ----------
Coendou_melanurus         ---------- ---------- ---------- ---------- ---------- ----------
Cavia_porcellus           ---------- ---------- ---------- ---------- ---------- ----------
Ctenomys_maulinus         ---------- ---------- ---------- ---------- ---------- ----------
Octodon_degus             ---------- ---------- ---------- ---------- ---------- ----------
Loxodonta_africana        ---------- ---------- ---------- ---------- ---------- ----------
Procavia_capensis         ---------- ---------- ---------- ---------- ---------- ----------
Echinops_telfairi         ---------- ---------- ---------- ---------- ---------- ----------
Micropotamogale           ---------- ---------- ---------- ---------- ---------- ----------
Myotis_lucifugus          ---------- ---------- ---------- ---------- ---------- ----------
Canis_familiaris          ---------- ---------- ---------- ---------- ---------- ----------
Felis_catus               ---------- ---------- ---------- ---------- ---------- ----------
Ovis_aries                ---------- ---------- ---------- ---------- ---------- ----------
Equus_caballus            ---------- ---------- ---------- ---------- ---------- ----------
Tapirus_terrestris        ---------- ---------- ---------- ---------- ---------- ----------
Vicugna                   ---------- ---------- ---------- ---------- ---------- ----------
Sus_scrofa                ---------- ---------- ---------- ---------- ---------- ----------
Bos_taurus                ---------- ---------- ---------- ---------- ---------- ----------
Tursiops_truncatus        ---------- ---------- ---------- ---------- ---------- ----------
Erinaceus                 ---------- ---------- ---------- ---------- ---------- ----------
Sorex_araneus
Neomys_anomalus           ---------- ---------- ---------- ---------- ---------- ----------
Choloepus_hoffmanni       ---------- ---------- ---------- ---------- ---------- ----------
Dasypus                   ---------- ---------- ---------- ---------- ---------- ----------
```

```
                         2221
Monodelphis              ---------- ---------- ---------- -------ATT AATA-CTTGG AAATATAACT
Macropus                 ---------- ---------- ---------- -------ATG AATA-CTTGG AAATATAACT
Microcebus_murinus       ---------- ---------- ---------- --------GT TT-------- CCAACTCCTG
Tarsius_syrichta         ---------- ---------- ---------- --------GT GC-------- AAGTATAGCC
Callithrix_jacchus       ---------- ---------- ---------- --------GT TA-------- AAATATAGCA
Macaca_mulatta           ---------- ---------- ---------- --------GT TA-------- AAATGTAGCA
Pongo_pygmaeus           ---------- ---------- ---------- --------GT TA-------- AAATGTAGCA
Gorilla_gorilla          ---------- ---------- ---------- ---------- ---------- ----------
Homo_sapiens             ---------- ---------- ---------- --------GT TA-------- AAATGTAGCA
Pan_troglodytes          ---------- ---------- ---------- --------GT TA-------- AAATGTAGCA
Ochotona_princeps        ---------- ---------- ---------- --------TT TA-------- AAATGTAGCC
Oryctolagus_cuniculus    ---------- ---------- ---------- --------GT TA-------- AAATGTAGCA
Marmota_monax            ---------- ---------- ---------- --------GT TA-------- AAATACAGTA
Aplodantia_rufa          ---------- ---------- ---------- ---------T AAATTAATTA AAATACGGCA
Spermophilus             ---------- ---------- ---------- --------NN NN-------- NNNNNNNNNN
Anomamorus               ---------- ---------- ---------- ---------- ---------- ----------
Maxomys                  AAAAATAATA GAAATAA-CT T-------AA ACTTTTAAAT AAAA------ ---TATAGCC
Rattus rattus            AAAAATAATA GAAATAA-CT T-------AG ACTTTTAAAT AAAA------ ---TATAGTC
Rattus exulans           AAAAATAATA GAAATAA-CT T-------AG ACTTTTAAAT AAAA------ ---CATAGTC
Rattus_norvegicus        AAAAATAATA GAAATAA-CT T-------AG ACTTTAAAAT AAAA------ ---TATAGTC
Mus_musculus             AAAAATAATA GAAATAA-CT T-------AA ACTTTTAAAT AAAA------ ---TATAGCC
Mus_spretus              ---------- ---------- ---------- ---------- ---------- ----------
Mus_pahari               TAAAATAATA GAAATAA-CT T-------AA ACTTTTAAAT AAAA------ ---TATAGCC
Praomys                  AAAAATAATA GAAATAA-TT T-------AA ACTTTTAAAT A--------- -----TAGCC
Meriones                 ----AAAATA GAAATA--CT TTTAAA---A GTTTTTAAAT AAAA----TA AAA-ATATCC
Meriones_crassus         ---------- ---------- ---------- ---------- ---------- ----------
Acomys cahirinus         AAAAATAATA GAAATAAACT TTTAAATTAA AATTTT-AAT AAAG----TA AAATATAGCC
Lophuromys sikapusi      AAAAATAATA GAAATAA-CT TTTAAATTAA A-TTTTTAAC AAAG----TA AAATATAGCC
Mesocricetus_auratus     AAAAAT---- ------AA-CT TTT---TTAA GTTTTAAAAT AAAGATATAG AAATAAAGCC
Oryzomys                 GAAAATAAT- -----AA-CT TTT----TAA ATTATAAAAT AAAA------ ---TATAGCC
Trichys_fasciculata      ---------- ---------- ---------- --------GC TT-------- AATTACAGCA
Heterocephalus_glaber    ---------- ---------- ---------- --------GC TT-------- AAATACAGCA
Coendou_melanurus        ---------- ---------- ---------- --------GC TC-------- AAATACAGCA
Cavia_porcellus          ---------- ---------- ---------- --------GC TC-------- GAATACAGCA
Ctenomys_maulinus        ---------- ---------- ---------- --------TT AC-------- AAATACAGAA
Octodon_degus            ---------- ---------- ---------- --------TT AA-------- AAATACAG-A
Loxodonta_africana       ---------- ---------- ---------- --------GT TA-------- AAATATAACA
Procavia_capensis        ---------- ---------- ---------- ---------- ---------- --------CA
Echinops_telfairi        ---------- ---------- ---------- --------CT GG-------- ----------
Micropotamogale          ---------- ---------- ---------- --------GT TC-------- --AAACTTCA
Myotis_lucifugus         ---------- ---------- ---------- --------TG CT-------- GAATGGGTTT
Canis_familiaris         ---------- ---------- ---------- --------GT TA-------- AAATAGATCT
Felis_catus              ---------- ---------- ---------- --------TT TA-------- AAATACAACT
Ovis_aries               ---------- ---------- ---------- ---------- ---------- ----------
Equus_caballus           ---------- ---------- ---------- --------AT TA-------- GAGTATAACC
Tapirus_terrestris       ---------- ---------- ---------- --------AT TA-------- GAGTATAACC
Vicugna                  ---------- ---------- ---------- --------GT TA-------- AAATACAACG
Sus_scrofa               ---------- ---------- ---------- --------GT GC-------- AAATGTAGCA
Bos_taurus               ---------- ---------- ---------- --------GT TA-------- AAATATAACC
Tursiops_truncatus       ---------- ---------- ---------- --------GT TA-------- AAATATAACA
Erinaceus                ---------- ---------- ---------- --------GT AA-------- AAGCAGAACA
Sorex_araneus
Neomys_anomalus          ---------- ---------- ---------- --------GT TG-------- TGATGTAACA
Choloepus_hoffmanni      ---------- ---------- ---------- ---------- -A-------- GAATACATTT
Dasypus                  ---------- ---------- ---------- --------TT TT-------- -AATATA---
```

```
                          2281
Monodelphis               AATTTAAG-- ---------- ---------- ---------- ---------- ----------
Macropus                  AATTTAAG-- ---------- ---------- ---------- ---------- ----------
Microcebus_murinus        ACCTCGAG-- ---------- ---------- ---------- ---------- ----------
Tarsius_syrichta          AGCTTAAC-- ---------- ---------- ---------- ---------- ----------
Callithrix_jacchus        ATCTTAAA-- ---------- ---------- ---------- ---------- ----------
Macaca_mulatta            ATCTTAAA-- ---------- ---------- ---------- ---------- ----------
Pongo_pygmaeus            ATCTTAAA-- ---------- ---------- ---------- ---------- ----------
Gorilla_gorilla           ---------- ---------- ---------- ---------- ---------- ----------
Homo_sapiens              ATCTTAAA-- ---------- ---------- ---------- ---------- ----------
Pan_troglodytes           ATCTTAAA-- ---------- ---------- ---------- ---------- ----------
Ochotona_princeps         ATTTTAAA-- ---------- ---------- ---------- ---------- ----------
Oryctolagus_cuniculus     ATATTAAA-- ---------- ---------- ---------- ---------- ----------
Marmota_monax             ATTTTAAA-- ---------- ---------- ---------- ---------- ----------
Aplodantia_rufa           A--------- ---------- ---------- ---------- ---------- ----------
Spermophilus              ---------- ---------- ---------- ---------- ---------- ----------
Anomamorus                ---------- ---------- ---------- ---------- ---------- ----------
Maxomys                   GTTTAAAA-- ---------- ---------- ---------- ---------- ----------
Rattus rattus             ATTTGAAA-- ---------- ---------- ---------- ---------- ----------
Rattus exulans            ATTTGAAA-- ---------- ---------- ---------- ---------- ----------
Rattus_norvegicus         ATTTGAAA-- ---------- ---------- ---------- ---------- ----------
Mus_musculus              ATTTTAAAAG ATCAGATTCG CCATGCAGTG GTGGCACATG CCTTTAACCC CAGCACTTGG
Mus_spretus               ---------- ---------- ---------- ---------- ---------- ----------
Mus_pahari                ATTTTAAAAG ATCAGATTCG CTGGGCAGTG GTGGCCCATG CCTTTAACCC CAGCACTTGG
Praomys                   ATTTTAAA-- ---------- ---------- ---------- ---------- ----------
Meriones                  ATTTTAAA-- ---------- ---------- ---------- ---------- ----------
Meriones_crassus          ---------- ---------- ---------- ---------- ---------- ----------
Acomys cahirinus          GTTTTAAA-- ---------- ---------- ---------- ---------- ----------
Lophuromys sikapusi       ATTTTAAA-- ---------- ---------- ---------- ---------- ----------
Mesocricetus_auratus      ATTTTAAA-- ---------- ---------- ---------- ---------- ----------
Oryzomys                  GTTTTAAA-- ---------- ---------- ---------- ---------- ----------
Trichys_fasciculata       ATATTAAA-- ---------- ---------- ---------- ---------- ----------
Heterocephalus_glaber     ATATTAAA-- ---------- ---------- ---------- ---------- ----------
Coendou_melanurus         ATATTAAA-- ---------- ---------- ---------- ---------- ----------
Cavia_porcellus           ATATTAAA-- ---------- ---------- ---------- ---------- ----------
Ctenomys_maulinus         ATAATAAA-- ---------- ---------- ---------- ---------- ----------
Octodon_degus             ATATTAAA-- ---------- ---------- ---------- ---------- ----------
Loxodonta_africana        ATTTAAAA-- ---------- ---------- ---------- ---------- ----------
Procavia_capensis         ATTTAATA-- ---------- ---------- ---------- ---------- ----------
Echinops_telfairi         ----ACAC-- ---------- ---------- ---------- ---------- ----------
Micropotamogale           AGTTAAAA-- ---------- ---------- ---------- ---------- ----------
Myotis_lucifugus          GTCCTAAA-- ---------- ---------- ---------- ---------- ----------
Canis_familiaris          ATTTTAAA-- ---------- ---------- ---------- ---------- ----------
Felis_catus               GTCTTTGG-- ---------- ---------- ---------- ---------- ----------
Ovis_aries                ---------- ---------- ---------- ---------- ---------- ----------
Equus_caballus            ATCTTAAA-- ---------- ---------- ---------- ---------- ----------
Tapirus_terrestris        ATCTTAAA-- ---------- ---------- ---------- ---------- ----------
Vicugna                   ATCTTAAA-- ---------- ---------- ---------- ---------- ----------
Sus_scrofa                ATCTTAAA-- ---------- ---------- ---------- ---------- ----------
Bos_taurus                ATCAT--A-- ---------- ---------- ---------- ---------- ----------
Tursiops_truncatus        TTCTTAAA-- ---------- ---------- ---------- ---------- ----------
Erinaceus                 GCTTTAAG-- ---------- ---------- ---------- ---------- ----------
Sorex_araneus
Neomys_anomalus           ATCTTAAG-- ---------- ---------- ---------- ---------- ----------
Choloepus_hoffmanni       TTAATACA-- ---------- ---------- ---------- ---------- ----------
Dasypus                   ----TAAA-- ---------- ---------- ---------- ---------- ----------
```

```
                          2341
Monodelphis               ---------- ---------- ---------- ---------- ---------- ---------T
Macropus                  ---------- ---------- ---------- ---------- ---------- ---------T
Microcebus_murinus        ---------- ------CA-- ---------- ---------- ---------- ----------
Tarsius_syrichta          ---------- ------TATA TTCTTTTTTT TTTTTTTTGG AAGCAGGATT CACGGAGAGG
Callithrix_jacchus        ---------- ------TATA TTCTT----- ---------- ---------- ----------
Macaca_mulatta            ---------- ------TATA TTCTT----- ---------- ---------- ----------
Pongo_pygmaeus            ---------- ------TATA TTCTT----- ---------- ---------- ----------
Gorilla_gorilla           ---------- ---------- ---------- ---------- ---------- ----------
Homo_sapiens              ---------- ------TATA TTCTT----- ---------- ---------- ----------
Pan_troglodytes           ---------- ------TATA TTCTT----- ---------- ---------- ----------
Ochotona_princeps         ---------- ------TATA TCCTT----- ---------- ---------- ----------
Oryctolagus_cuniculus     ---------- ------TATA TTCTT----- ---------- ---------- ----------
Marmota_monax             ---------- ---------- ---------- ---------- ---------- ----------
Aplodantia_rufa           ---------- ---------- ---------- ---------- ---------- ----------
Spermophilus              ---------- ---------- ---------- ---------- ---------- ----------
Anomamorus                ---------- ---------- ---------- ---------- ---------- ----------
Maxomys                   ---------- ---------- ---------- ---------- ---------- ----------
Rattus rattus             ---------- ---------- ---------- ---------- ---------- ----------
Rattus exulans            ---------- ---------- ---------- ---------- ---------- ----------
Rattus_norvegicus         ---------- ---------- ---------- ---------- ---------- ----------
Mus_musculus              CAGGCAGAGG CAGGTGGATT TCTGAGTTCT ACAGAGTGAG TTCCAGGACA GCCAGGGCTA
Mus_spretus               ---------- ---------- ---------- ---------- ---------- ----------
Mus_pahari                GAGGGAGAGG CAGGCGGATT TYTGAGTTYT ACAGAGTGAG TTCCAGGACA GCCAGGGCTA
Praomys                   ---------- ---------- ---------- ---------- ---------- ----------
Meriones                  ---------- ---------- ---------- ---------- ---------- ----------
Meriones_crassus          ---------- ---------- ---------- ---------- ---------- ----------
Acomys cahirinus          ---------- ---------- ---------- ---------- ---------- ----------
Lophuromys sikapusi       ---------- ---------- ---------- ---------- ---------- ----------
Mesocricetus_auratus      ---------- ---------- ---------- ---------- ---------- ----------
Oryzomys                  ---------- ---------- ---------- ---------- ---------- ----------
Trichys_fasciculata       ---------- ------TATA ATCTT----- ---------- ---------- ----------
Heterocephalus_glaber     ---------- ------TATA ATCGT----- ---------- ---------- ----------
Coendou_melanurus         ---------- ------TATA ATCTT----- ---------- ---------- ----------
Cavia_porcellus           ---------- ------TATA ATTTC----- ---------- ---------- ----------
Ctenomys_maulinus         ---------- ------TATA ATCTT----- ---------- ---------- ----------
Octodon_degus             ---------- ------TATA ATCTT----- ---------- ---------- ----------
Loxodonta_africana        ---------- ------TGTA TTCTT----- ---------- ---------- ----------
Procavia_capensis         ---------- ------TGCA TTCTT----- ---------- ---------- ----------
Echinops_telfairi         ---------- ------TAGA CCTGC----- ---------- ---------- ----------
Micropotamogale           ---------- ------TGCA TCCGT----- ---------- ---------- ----------
Myotis_lucifugus          ---------- ------TA-- ---------- ---------- ---------- ----------
Canis_familiaris          ---------- ------TATA TTCTT----- ---------- ---------- ----------
Felis_catus               ---------- ------GA-- ---------- ---------- ---------- ----------
Ovis_aries                ---------- ---------- ---------- ---------- ---------- ----------
Equus_caballus            ---------- ------TATA CTCTT----- ---------- ---------- ----------
Tapirus_terrestris        ---------- ------TATA TTCTT----- ---------- ---------- ----------
Vicugna                   ---------- ------TATA TTCTT----- ---------- ---------- ----------
Sus_scrofa                ---------- ------TACA TTCTT----- ---------- ---------- ----------
Bos_taurus                ---------- ------TATA TTCTG----- ---------- ---------- ----------
Tursiops_truncatus        ---------- ------AATA TTCTT----- ---------- ---------- ----------
Erinaceus                 ---------- ------TAGA TGCTT----- ---------- ---------- ----------
Sorex_araneus
Neomys_anomalus           ---------- ------TATC TTCTT----- ---------- ---------- ----------
Choloepus_hoffmanni       ---------- ------CATT ---------- ---------- ---------- ----------
Dasypus                   ---------- ------TATA T--------- ---------- ---------- ----------
```

```
                         2401
Monodelphis                 ATATTCTTTA TATGTACATA TAGA------ -------ATA GCTCATGTAC ATACATATCT
Macropus                    ATATCTTCTT TATGTACATA CATACATATG TACTACTACA GCTTCTATAC ATACATTCAC
Microcebus_murinus          ---------- ---------- ---------- ---------- ---------- ----------
Tarsius_syrichta            ACAGAAAAGC TCCCACAGTG GGAGGGGACC CGAAGGGGCT GCCCTT---- ------GACT
Callithrix_jacchus          ---------- ---------- ---------- ---------- ---------- ----------
Macaca_mulatta              ---------- ---------- ---------- ---------- ---------- ----------
Pongo_pygmaeus              ---------- ---------- ---------- ---------- ---------- ----------
Gorilla_gorilla             ---------- ---------- ---------- ---------- ---------- ----------
Homo_sapiens                ---------- ---------- ---------- ---------- ---------- ----------
Pan_troglodytes             ---------- ---------- ---------- ---------- ---------- ----------
Ochotona_princeps           ---------- ---------- ---------- ---------- ---------- ----------
Oryctolagus_cuniculus       ---------- ---------- ---------- ---------- ---------- ----------
Marmota_monax               ---------- ---------- ---------- ---------- ---------- ----------
Aplodantia_rufa             ---------- ---------- ---------- ---------- ---------- ----------
Spermophilus                ---------- ---------- ---------- ---------- ---------- ----------
Anomamorus                  ---------- ---------- ---------- ---------- ---------- ----------
Maxomys                     ---------- ---------- ---------- ---------- ---------- ----------
Rattus rattus               ---------- ---------- ---------- ---------- ---------- ----------
Rattus exulans              ---------- ---------- ---------- ---------- ---------- ----------
Rattus_norvegicus           ---------- ---------- ---------- ---------- ---------- ----------
Mus_musculus                TACAGAGAAA CCCTATCTCG AAAAAACCCA AA-AAACAAA AAACAAAAAA ATAAAAAAAT
Mus_spretus                 ---------- ---------- ---------- ---------- ---------- ----------
Mus_pahari                  TACAGAGAAA CCCTGTYTCA GAAAAAAACA AACAAACAAA CAAACAAACC AAACAAACAA
Praomys                     ---------- ---------- ---------- ---------- ---------- ----------
Meriones                    ---------- ---------- ---------- ---------- ---------- ----------
Meriones_crassus            ---------- ---------- ---------- ---------- ---------- ----------
Acomys cahirinus            ---------- ---------- ---------- ---------- ---------- ----------
Lophuromys sikapusi         ---------- ---------- ---------- ---------- ---------- ----------
Mesocricetus_auratus        ---------- ---------- ---------- ---------- ---------- ----------
Oryzomys                    ---------- ---------- ---------- ---------- ---------- ----------
Trichys_fasciculata         ---------- ---------- ---------- ---------- ---------- ----------
Heterocephalus_glaber       ---------- ---------- ---------- ---------- ---------- ----------
Coendou_melanurus           ---------- ---------- ---------- ---------- ---------- ----------
Cavia_porcellus             ---------- ---------- ---------- ---------- ---------- ----------
Ctenomys_maulinus           ---------- ---------- ---------- ---------- ---------- ----------
Octodon_degus               ---------- ---------- ---------- ---------- ---------- ----------
Loxodonta_africana          ---------- ---------- ---------- ---------- ---------- ----------
Procavia_capensis           ---------- ---------- ---------- ---------- ---------- ----------
Echinops_telfairi           ---------- ---------- ---------- ---------- ---------- ----------
Micropotamogale             ---------- ---------- ---------- ---------- ---------- ----------
Myotis_lucifugus            ---------- ---------- ---------- ---------- ---------- ----------
Canis_familiaris            ---------- ---------- ---------- ---------- ---------- ----------
Felis_catus                 ---------- ---------- ---------- ---------- ---------- ----------
Ovis_aries                  ---------- ---------- ---------- ---------- ---------- ----------
Equus_caballus              ---------- ---------- ---------- ---------- ---------- ----------
Tapirus_terrestris          ---------- ---------- ---------- ---------- ---------- ----------
Vicugna                     ---------- ---------- ---------- ---------- ---------- ----------
Sus_scrofa                  ---------- ---------- ---------- ---------- ---------- ----------
Bos_taurus                  ---------- ---------- ---------- ---------- ---------- ----------
Tursiops_truncatus          ---------- ---------- ---------- ---------- ---------- ----------
Erinaceus                   ---------- ---------- ---------- ---------- ---------- ----------
Sorex_araneus
Neomys_anomalus             ---------- ---------- ---------- ---------- ---------- ----------
Choloepus_hoffmanni         ---------- ---------- ---------- ---------- ---------- ----------
Dasypus                     ---------- ---------- ---------- ---------- ---------- ----------
```

```
                              2461
Monodelphis                   ATATATGTAT GTGTA----- -----TGTGT CTATTTTT-- CTATCTCTC- CATATCTTTC
Macropus                      ATATATATGC ACATATACAT AAATATGTGT GTCTGTCTCT CTATCTACC- TATGCCTTTC
Microcebus_murinus            ---------- ---------- ---------- -------ATC CACCCGCCTC TGCCTCCCAG
Tarsius_syrichta              ATACTCTTAA GAACC----- ---------- -----GACTC CGATCAG--- TGCTCACTTA
Callithrix_jacchus            --------CA TAACC----- ---------- -----AATTC CAACGTG--- TTCTCACTTA
Macaca_mulatta                --------CA TAACC----- ---------- -----AATTC CAACGTG--- TTCCCCCTTA
Pongo_pygmaeus                --------CA TAACC----- ---------- -----AATTC CAATGTG--- TTCTCACTTA
Gorilla_gorilla               ---------- ---------- ---------- ---------- ---------- ----------
Homo_sapiens                  --------CA TAACC----- ---------- -----AATTC CAACGTG--- TTCTCACTTA
Pan_troglodytes               --------CA TAACC----- ---------- -----AATTC CAACGTG--- TTCTCACTTA
Ochotona_princeps             --------AA TAAAT----- ---------- -----GATTG CAATC----- TGCTTATTTA
Oryctolagus_cuniculus         --------AA TAAAT----- ---------- -----TATTG CAATCAA--- TGCTCACTTA
Marmota_monax                 ---------- ---------- ---------- ----CGTTCC CAATTTG--- GGATCACTTA
Aplodantia_rufa               ---------- ---------- ---------- ---------- -----TG--- GGATCACTTA
Spermophilus                  ---------- ---------- ---------- ---------- ---------- ----------
Anomamorus                    ---------- ---------- ---------- ---------- ---------- ----------
Maxomys                       ---------- ---------- ---------- ----ATATCA GAATCTC--- TGCTC--TTA
Rattus rattus                 ---------- ---------- ---------- -----GATCA GATTCTC--- TGCTC--TTA
Rattus exulans                ---------- ---------- ---------- -----GATCA GATTCTC--- TGTTC--TTA
Rattus_norvegicus             ---------- ---------- ---------- -----GATCA GATTCTC--- TGCTC--TTA
Mus_musculus                  AAA------- ---------- ---------- ----AGATCA GATTCTC--- TGCTC--TTA
Mus_spretus                   ---------- ---------- ---------- ---------- ---------- ----------
Mus_pahari                    AATAAA---- ---------- ---------- -----AGATC AGATTYTY-- TGCTC--TTA
Praomys                       ---------- ---------- ---------- ----AGATCA GAATCTC--- TGCTC--TTA
Meriones                      ---------- ---------- ---------- ----CGAACT TATTACC--- TGATCGCTTA
Meriones_crassus              ---------- ---------- ---------- ---------- ---------- ----------
Acomys cahirinus              ---------- ---------- ---------- ----TGATCG GATTCTC--- TGCTCACTTA
Lophuromys sikapusi           ---------- ---------- ---------- ----TGATCG GATTCTC--- TGCTCACTTA
Mesocricetus_auratus          ---------- ---------- ---------- ----CAATCA CATTCTC--- TGCTCACTTA
Oryzomys                      ---------- ---------- ---------- ----CGATCA CATTCTC--- TGCTCATTTA
Trichys_fasciculata           --------CA TAAAC----- ---------- -----GATCC CAGCCTG--- TGCTCACTTA
Heterocephalus_glaber         --------CA TAAAC----- ---------- -----GATCC CAGTCAG--- TGCTCACTTA
Coendou_melanurus             --------CA TAAAC----- ---------- -----GATCC CAGTCAG--- TGCTCACTTA
Cavia_porcellus               --------TA CAAAT----- ---------- -----GATCC CAGGCAG--- TGCTCATTTA
Ctenomys_maulinus             --------CA ---------- ---------- ---------- ---------- -------TAA
Octodon_degus                 --------CA TAAAC----- ---------- -----AATCA G--------- TGCTCACTTA
Loxodonta_africana            AA--TATATA TAAAT----- ---------- -ATA-AATTC CGATCTA--- TGCTCATTTA
Procavia_capensis             ACTATATGTA TTAAT----- ---------- -ATGCGATTA CACTTTA--- TGCTCACTTA
Echinops_telfairi             ----TATGCA TGAAA----- ---------- ---GAGACCT CAGTGTG--- TGC-CA-CCC
Micropotamogale               AC--TACCTA GCAAT----- ---------- -ATGTCATCC CAATCTG--- TGCTCACTTC
Myotis_lucifugus              ---------- ---------- ---------- ---------- ---------- TGGGCCCTCG
Canis_familiaris              --------GG TCAAC----- ---------- -----GATTC CAATCTG--- TGCTCACTTA
Felis_catus                   ---------- ---------- ---------- ---------- -AGCCTGGGT GGCTCATTGG
Ovis_aries                    ---------- ---------- ---------- ---------- ---------- ----------
Equus_caballus                --------AG TAACC----- ---------- -----GATTC CAATCTG--- TGCTCACGTA
Tapirus_terrestris            --------AG TAAAC----- ---------- -----GATTC CAATCTR--- TGCTCATGTA
Vicugna                       --------AG TAAAC----- ---------- -----CATTC CAATCTG--- TGCAC-----
Sus_scrofa                    --------AG TAAAC----- ---------- -----GATTC CAATCTG--- TGCGCAGGTG
Bos_taurus                    --------AG TAAGC----- ---------- -----GATTC CAGTCGT--- -GCGCACGTA
Tursiops_truncatus            --------AG TGGAA----- ---------- -----GATTC CAATCTG--- TGCGCACGTA
Erinaceus                     --------AG TAAGC----- ---------- -----AATAC CAGTTTG--- TGCTCATGTA
Sorex_araneus
Neomys_anomalus               --------AG TAAAA----- ---------- -----GC-TC CTATTGG--- -GTTCACATC
Choloepus_hoffmanni           --------AG TATAT----- ---------- -----GATTC CAGACTG--- TGTGCTCTTA
Dasypus                       ---------- ---------- ---------- -----GATTC CCCTCTG--- GGTTCTCTTC
```

```
                          2521
Monodelphis               AGTAGTTTGA TTTAAGAGAT ATAGAACTAG CCTT------ ---------- ----------
Macropus                  AGCATCATGG TTTAACCGAT ATACAGCCAA TCTT------ ---------- ----------
Microcebus_murinus        AGTGCTAGGA TTACAGGCGT GAGCCACCGC GCCC------ ---------- ----------
Tarsius_syrichta          AGTGAG-GGC ATGAA----- -----ACCCC ATCA------ ---------- ----------
Callithrix_jacchus        AGTCCTGGGT A--------- ---------- --TT------ ---------- ----------
Macaca_mulatta            AGTAATGGGT ATAAA----- -----ACCCC ACTT------ ---------- ----------
Pongo_pygmaeus            AGTAATGGGT ATAAA----- -----ACCCC ACTT------ ---------- ----------
Gorilla_gorilla           ---------- ---------- ---------- ---------- ---------- ----------
Homo_sapiens              AGTAATGGGT ATAAA----- -----ACCCC ACTT------ ---------- ----------
Pan_troglodytes           AGTAATGGGT ATAAA----- -----ACCCC ACTT------ ---------- ----------
Ochotona_princeps         AGGAAATAGT ATGAA----- -----TTCGC ATTT------ ---------- ----------
Oryctolagus_cuniculus     AGGAAGTAGT ATAAA----- -----ATCCC ATTT------ ---------- ----------
Marmota_monax             ACG------- ATACA----- -----ATATC ATTT------ ---------- ----------
Aplodantia_rufa           G----TTGTC ACACA----- -----ATCCC ATTC------ ---------- ----------
Spermophilus              ---------- ---------- ---------- ---------- ---------- ----------
Anomamorus                ---------- ---------- ---------- ---------- ---------- ----------
Maxomys                   ATTAGAA--- -TAAA----- -----ATCCA ATTT------ ---------- ----------
Rattus rattus             ATTAGAA--- -TGAA----- -----ATCCC ATTT------ ---------- ----------
Rattus exulans            ATTAGAA--- -TGAA----- -----ATCCC ATTT------ ---------- ----------
Rattus_norvegicus         ATTAGAA--- -TGAA----- -----ATCCC ATTT------ ---------- ----------
Mus_musculus              ATTAGAA--- -TAAA----- -----ATCCC ATTT------ ---------- ----------
Mus_spretus               ---------- ---------- ---------- ---------- ---------- ----------
Mus_pahari                ATTAGAA--- -TAAA----- -----ATCCC ATTTAGAAGG AACYACAGAC CAAACCTCAC
Praomys                   ATTAGAA--- -TAAC----- -----ATCCC ATTT------ ---------- ----------
Meriones                  ATTAGCA--- -TAAA----- -----ATCCC ATTT------ ---------- ----------
Meriones_crassus          ---------- ---------- ---------- ---------- ---------- ----------
Acomys cahirinus          ATTAGTA--- -TAAA----- -----ATTCC ATTT------ ---------- ----------
Lophuromys sikapusi       ATTAGCA--- -TAAAA---- -----ATTCC ATTT------ ---------- ----------
Mesocricetus_auratus      ----GCA--- -TAAA----- -----ATCCC ACTT------ ---------- ----------
Oryzomys                  C----CA--- -TAAA----- -----ATCCC ACTT------ ---------- ----------
Trichys_fasciculata       AGCAAAAAGT TTAAA----- -----AT-CC GTTT------ ---------- ----------
Heterocephalus_glaber     AGAAAAAGTT TTAAA----- -----AT-CC GTTT------ ---------- ----------
Coendou_melanurus         AGCAAAAGGT ATAAA----- -----AT-CC GTGG------ ---------- ----------
Cavia_porcellus           AGCAGAAGGT TTAAA----- -----AT-CT GTTG------ ---------- ----------
Ctenomys_maulinus         GCAAAACGGT TTAAA----- -----AT-CT GCTG------ ---------- ----------
Octodon_degus             AGCAAAAAGG TTTAA----- -----AATCC GCTG------ ---------- ----------
Loxodonta_africana        AATAATTGGT ATAAA----- -----ATCAC TTTT------ ---------- ----------
Procavia_capensis         AATAGTTGGT ATAAA----- -----ATCAT CTTT------ ---------- ----------
Echinops_telfairi         CTGAGATCTT GTATA----- ---TGATCTT T--------- ---------- ----------
Micropotamogale           AGTAGTTGGT ATAAA----- -----AGCAC TTT------- ---------- ----------
Myotis_lucifugus          AGCTATTGCA CAGAC----- -----ACCAG CATCTCCA-- ---------- ----------
Canis_familiaris          AGTAAGCGGC GCATA----- -----ATCCT TTTC------ ---------- ----------
Felis_catus               GTTAAGCGCC GGACG----- -----AC--- ---------- ---------- ----------
Ovis_aries                ---------- ---------- ---------- ---------- ---------- ----------
Equus_caballus            AGTAAGTGGT ATAAA----- ----AATCCC TTT------- ---------- ----------
Tapirus_terrestris        AGTAACTGGT ATAAA----- -----ATCCC TTT------- ---------- ----------
Vicugna                   AGGAATTGCT ATAAGATTAA ATTAAATTCA AATTTTAA-- ---------- ----------
Sus_scrofa                A--------- ---------- --------TT TTTC------ ---------- ----------
Bos_taurus                ACT---GGAA TAAA------ ----ATGAAA TGA------- ---------- ----------
Tursiops_truncatus        A----TTGGT ATAAAATTA- ---------- ---------- ---------- ----------
Erinaceus                 ATT-----GA TAGAAA---- ------ATCC TTTTT----- ---------- ----------
Sorex_araneus
Neomys_anomalus           GACAGGATCC TTGAAAGGGG T--GAAACCC CTTT------ ---------- ----------
Choloepus_hoffmanni       AATAGTTAGC ATAAA----- -----ATCAC TTTT------ ---------- ----------
Dasypus                   CTTCGTTGGG CTAAA----- -----ACCAC GTTT------ ---------- ----------
```

```
                         2581
Monodelphis              ---------- --------GA AC----GAAG ACCTGAGTTC AAGTCCTATT TCTGACCTTT
Macropus                 ---------- --------GG ACTTAGGAAG ATCTGAGTTC AAGTCTTGTC TGTGACCTTT
Microcebus_murinus       ---------- ---------- ---------- ----GGCCCA GAGCTGAGTT T---------
Tarsius_syrichta         ---------- ---------- ---------- ----ACACGA AAACTGCGTT TT--------
Callithrix_jacchus       ---------- ---------- ---------- ----GAACGT AT---TATTT TAAATATT--
Macaca_mulatta           ---------- ---------- ---------- ----AAACGT AA---TATTT TAAATATT--
Pongo_pygmaeus           ---------- ---------- ---------- ----AAACGT AC---TATTT TAAATATT--
Gorilla_gorilla          ---------- ---------- ---------- ---------- ---------- ----------
Homo_sapiens             ---------- ---------- ---------- ----AAGCGT AA---TATTT TAAATATT--
Pan_troglodytes          ---------- ---------- ---------- ----AAACGT AA---TATTT TAAATATT--
Ochotona_princeps        ---------- ---------- ---------- ----ACATTA AA---TAATT TGATTTTT--
Oryctolagus_cuniculus    ---------- ---------- ---------- ----GAATTA AA---CAACT TAAATTTT--
Marmota_monax            ---------- ---------- ---------- ----AAATTA ------AGCC CCAATTTT--
Aplodantia_rufa          ---------- ---------- ---------- ----AAATTA AACCCTAATT TT--------
Spermophilus             ---------- ---------- ---------- ---------- ---------- ----------
Anomamorus               ---------- ---------- ---------- ---------- ---------- ----------
Maxomys                  ---------- ---------- ---------- ----AAACTA AATCTTAGTT CACTT-----
Rattus rattus            ---------- ---------- ---------- ----AAACTA AATCTTAATT CGTTT-----
Rattus exulans           ---------- ---------- ---------- ----AAACTA AATCTTAATT CGTTT-----
Rattus_norvegicus        ---------- ---------- ---------- ----AAACTA AATCTTAATT CGTTT-----
Mus_musculus             ---------- ---------- ---------- ----AAGCTA AATCTTAATT CGTTT-----
Mus_spretus              ---------- ---------- ---------- ---------- ---------- ----------
Mus_pahari               TTTCTAAACT TTGMTAGATT GTGATCAGTT CCTAAAACTA AATCTGGCTG GGTACGGTGG
Praomys                  ---------- ---------- ---------- ----AAACTA AATCTTAATT TGTTT-----
Meriones                 ---------- ---------- ---------- ----AAACTA AACCTTAATT CGTTT-----
Meriones_crassus         ---------- ---------- ---------- ---------- ---------- ----------
Acomys cahirinus         ---------- ---------- ---------- ----AAAGTA AACCTTAGTT TGTTT-----
Lophuromys sikapusi      ---------- ---------- ---------- ----AAACTA AAGCTT---- CGTTT-----
Mesocricetus_auratus     ---------- ---------- ---------- ----AAACTA AATCTCAATT CGTTC-----
Oryzomys                 ---------- ---------- ---------- ----AAGCTA AATCTCAATT TGTTC-----
Trichys_fasciculata      ---------- ---------- ---------- ----AATTTA AACCTTAATT TATATATA--
Heterocephalus_glaber    ---------- ---------- ---------- ----AAATGA AAACTTAATA AGAATAT---
Coendou_melanurus        ---------- ---------- ---------- ----AAATTA AAGCTTAATT AAAAAAT---
Cavia_porcellus          ---------- ---------- ---------- ----AAATTA AAGCTTATTT TTTTTTTAAA
Ctenomys_maulinus        ---------- ---------- ---------- ----AAATTA AAGCTTAATT TTTT------
Octodon_degus            ---------- ---------- ---------- ----AAATTA AAGCTTAATT TTTTTTTT--
Loxodonta_africana       ---------- ---------- ---------- ----AAATTA AACCTTCATT TTTTTTTT--
Procavia_capensis        ---------- ---------- ---------- ----AAACT- --------TT TTTTTTTT--
Echinops_telfairi        ---------- ---------- ---------- ---------- ---------- ----------
Micropotamogale          ---------- ---------- ---------- -----AAATC ACACTGCTT- ----------
Myotis_lucifugus         ---------- ---------- ---------- ----AACCCA GAGCCTGATC ----------
Canis_familiaris         ---------- ---------- ---------- ----CAGCTC AAGGGAACTG TCGAGCTT--
Felis_catus              ---------- ---------- ---------- ---------- ---------- ------TG--
Ovis_aries               ---------- ---------- ---------- ---------- ---------- ----------
Equus_caballus           ---------- ---------- ---------- -----TAATA AACCTCAGGG TTTTTT----
Tapirus_terrestris       ---------- ---------- ---------- -----TAATA AACCACAGTT TTTTT-----
Vicugna                  ---------- ---------- ---------- ----AAATTA AACCTTAGTT TTTTT-----
Sus_scrofa               ---------- ---------- -----CAAAA TTAAAATTTA AACCTT---- ----------
Bos_taurus               ---------- ---------- ---------- ----AAATTA AAATTTTAAA AGACCTTTTT
Tursiops_truncatus       ---------- ---------- -----AAATT TAAAAAATTA GACCTT---- ----------
Erinaceus                ---------- ---------- ---------- -----AAATA AACCTTATTT TTTAATTTTC
Sorex_araneus
Neomys_anomalus          ---------- ---------- ---------- ---------- ---------- ----------
Choloepus_hoffmanni      ---------- ---------- ---------- ----AAATTA AGCTGTACTT TTTTTTTT--
Dasypus                  ---------- ---------- ---------- ----CAACTA AACCCTACCC TTTTTT----
```

```
                         2641
Monodelphis              GATGCATATA AG-TTTTGTG ATCCTAGATA AATTACCTAA CCTCTCACTA CCTCAGGCAA
Macropus                 GATACATACT AGCTTTTGTG ACCCAGGGTA AATTACCTAA GTTCTTACTA CCTCAGGCAA
Microcebus_murinus       ---------- ---------- ---------- ---------- ---------- ----------
Tarsius_syrichta         ---------- ---------- ---------- ---------- ---------- ----------
Callithrix_jacchus       ---------- ---------- ---------- ---------- ---------- ----------
Macaca_mulatta           ---------- ---------- ---------- ---------- ---------- ----------
Pongo_pygmaeus           ---------- ---------- ---------- ---------- ---------- ----------
Gorilla_gorilla          ---------- ---------- ---------- ---------- ---------- ----------
Homo_sapiens             ---------- ---------- ---------- ---------- ---------- ----------
Pan_troglodytes          ---------- ---------- ---------- ---------- ---------- ----------
Ochotona_princeps        ---------- ---------- ---------- ---------- ---------- ----------
Oryctolagus_cuniculus    ---------- ---------- ---------- ---------- ---------- ----------
Marmota_monax            ---------- ---------- ---------- ---------- ---------- ----------
Aplodantia_rufa          ---------- ---------- ---------- ---------- ---------- ----------
Spermophilus             ---------- ---------- ---------- ---------- ---------- ----------
Anomamorus               ---------- ---------- ---------- ---------- ---------- ----------
Maxomys                  ---------- ---------- ---------- ---------- ---------- ----------
Rattus rattus            ---------- ---------- ---------- ---------- ---------- ----------
Rattus exulans           ---------- ---------- ---------- ---------- ---------- ----------
Rattus_norvegicus        ---------- ---------- ---------- ---------- ---------- ----------
Mus_musculus             ---------- ---------- ---------- ---------- ---------- ----------
Mus_spretus              ---------- ---------- ---------- ---------- ---------- ----------
Mus_pahari               TACTCACCTT CAAAGCCAGT GCTCCGAAGC TAAGGCTCAG ACCATSACGC AGCARGCTGC
Praomys                  ---------- ---------- ---------- ---------- ---------- ----------
Meriones                 ---------- ---------- ---------- ---------- ---------- ----------
Meriones_crassus         ---------- ---------- ---------- ---------- ---------- ----------
Acomys cahirinus         ---------- ---------- ---------- ---------- ---------- ----------
Lophuromys sikapusi      ---------- ---------- ---------- ---------- ---------- ----------
Mesocricetus_auratus     ---------- ---------- ---------- ---------- ---------- ----------
Oryzomys                 ---------- ---------- ---------- ---------- ---------- ----------
Trichys_fasciculata      ---------- ---------- ---------- ---------- ---------- ----------
Heterocephalus_glaber    ---------- ---------- ---------- ---------- ---------- ----------
Coendou_melanurus        ---------- ---------- ---------- ---------- ---------- ----------
Cavia_porcellus          A--------- ---------- ---------- ---------- ---------- ----------
Ctenomys_maulinus        ---------- ---------- ---------- ---------- ---------- ----------
Octodon_degus            ---------- ---------- ---------- ---------- ---------- ----------
Loxodonta_africana       ---------- ---------- ---------- ---------- ---------- ----------
Procavia_capensis        ---------- ---------- ---------- ---------- ---------- ----------
Echinops_telfairi        ---------- ---------- ---------- ---------- ---------- ----------
Micropotamogale          ---------- ---------- ---------- ---------- ---------- ----------
Myotis_lucifugus         ---------- ---------- ---------- ---------- ---------- ----------
Canis_familiaris         ---------- ---------- ---------- ---------- ---------- ----------
Felis_catus              ---------- ---------- ---------- ---------- ---------- ----------
Ovis_aries               ---------- ---------- ---------- ---------- ---------- ----------
Equus_caballus           ---------- ---------- ---------- ---------- ---------- ----------
Tapirus_terrestris       ---------- ---------- ---------- ---------- ---------- ----------
Vicugna                  ---------- ---------- ---------- ---------- ---------- ----------
Sus_scrofa               ---------- ---------- ---------- ---------- ---------- ----------
Bos_taurus               TTTTTTTGCT TTTAAATATT TTTTAATACA AAAATACCCA TACACAATCT TCCAAACACA
Tursiops_truncatus       ---------- ---------- ---------- ---------- ---------- ----------
Erinaceus                CTTATTTGTT TTT-GGATAG AAAGAAATTG AGAGGTAAGC AGGAGGTAGG CGAGAGTGCG
Sorex_araneus
Neomys_anomalus          ---------- ---------- ---------- ---------- ---------- ----------
Choloepus_hoffmanni      ---------- ---------- ---------- ---------- ---------- ----------
Dasypus                  ---------- ---------- ---------- ---------- ---------- ----------
```

```
                           2701
Monodelphis                TTCTGAAAAT ACAGGTTATG GGAAATTTCC ACACCAAGAG TTCCCTACAC CAACAAAATC
Macropus                   CTTTGAAAAT ACAAGTTATG GGAAAATTCC ACACCAAGAG TTCCCTACAC CAACAAAATC
Microcebus_murinus         ---------- ---------- ---------- ---------- ---------- ----------
Tarsius_syrichta           ---------- ---------- ---------- ---------- ---------- ----------
Callithrix_jacchus         ---------- ---------- ---------- ---------- ---------- ----------
Macaca_mulatta             ---------- ---------- ---------- ---------- ---------- ----------
Pongo_pygmaeus             ---------- ---------- ---------- ---------- ---------- ----------
Gorilla_gorilla            ---------- ---------- ---------- ---------- ---------- ----------
Homo_sapiens               ---------- ---------- ---------- ---------- ---------- ----------
Pan_troglodytes            ---------- ---------- ---------- ---------- ---------- ----------
Ochotona_princeps          ---------- ---------- ---------- ---------- ---------- ----------
Oryctolagus_cuniculus      ---------- ---------- ---------- ---------- ---------- ----------
Marmota_monax              ---------- ---------- ---------- ---------- ---------- ----------
Aplodantia_rufa            ---------- ---------- ---------- ---------- ---------- ----------
Spermophilus               ---------- ---------- ---------- ---------- ---------- ----------
Anomamorus                 ---------- ---------- ---------- ---------- ---------- ----------
Maxomys                    ---------- ---------- ---------- ---------- ---------- ----------
Rattus rattus              ---------- ---------- ---------- ---------- ---------- ----------
Rattus exulans             ---------- ---------- ---------- ---------- ---------- ----------
Rattus_norvegicus          ---------- ---------- ---------- ---------- ---------- ----------
Mus_musculus               ---------- ---------- ---------- ---------- ---------- ----------
Mus_spretus                ---------- ---------- ---------- ---------- ---------- ----------
Mus_pahari                 TA-------- ---------- ---------- ---------- ---------- ----------
Praomys                    ---------- ---------- ---------- ---------- ---------- ----------
Meriones                   ---------- ---------- ---------- ---------- ---------- ----------
Meriones_crassus           ---------- ---------- ---------- ---------- ---------- ----------
Acomys cahirinus           ---------- ---------- ---------- ---------- ---------- ----------
Lophuromys sikapusi        ---------- ---------- ---------- ---------- ---------- ----------
Mesocricetus_auratus       ---------- ---------- ---------- ---------- ---------- ----------
Oryzomys                   ---------- ---------- ---------- ---------- ---------- ----------
Trichys_fasciculata        ---------- ---------- ---------- ---------- ---------- ----------
Heterocephalus_glaber      ---------- ---------- ---------- ---------- ---------- ----------
Coendou_melanurus          ---------- ---------- ---------- ---------- ---------- ----------
Cavia_porcellus            ---------- ---------- ---------- ---------- ---------- ----------
Ctenomys_maulinus          ---------- ---------- ---------- ---------- ---------- ----------
Octodon_degus              ---------- ---------- ---------- ---------- ---------- ----------
Loxodonta_africana         ---------- ---------- ---------- ---------- ---------- ----------
Procavia_capensis          ---------- ---------- ---------- ---------- ---------- ----------
Echinops_telfairi          ---------- ---------- ---------- ---------- ---------- ----------
Micropotamogale            ---------- ---------- ---------- ---------- ---------- ----------
Myotis_lucifugus           ---------- ---------- ---------- ---------- ---------- ----------
Canis_familiaris           ---------- ---------- ---------- ---------- ---------- ----------
Felis_catus                ---------- ---------- ---------- ---------- ---------- ----------
Ovis_aries                 ---------- ---------- ---------- ---------- ---------- ----------
Equus_caballus             ---------- ---------- ---------- ---------- ---------- ----------
Tapirus_terrestris         ---------- ---------- ---------- ---------- ---------- ----------
Vicugna                    ---------- ---------- ---------- ---------- ---------- ----------
Sus_scrofa                 ---------- ---------- ---------- ---------- ---------- ----------
Bos_taurus                 AAGGCCACTC TCGGCCTTTG GGGTACCATG TGCTACCGCC AGTTTTGAGA CAAAAACGTG
Tursiops_truncatus         ---------- ---------- ---------- ---------- ---------- ----------
Erinaceus                  AGAGAG---- ---------- ---------- ---------- ---------- ----------
Sorex_araneus
Neomys_anomalus            ---------- ---------- ---------- ---------- ---------- ----------
Choloepus_hoffmanni        ---------- ---------- ---------- ---------- ---------- ----------
Dasypus                    ---------- ---------- ---------- ---------- ---------- ----------
```

```
                         2761
Monodelphis                 ATAGGTGCAG ATCAAAATAT GCCAATTTGA TTCAATTCAG CAAA----TA TTAAATGCAA
Macropus                    ATAGATCCAG ACCAAAATAC ACTGATTTGA TTCAATTCAG CAAATATTTA TTAAATGCTA
Microcebus_murinus          ---------- ---------- ---------- ---------- ---------- ----------
Tarsius_syrichta            ---------- ---------- ---------- ---------- ---------- ----------
Callithrix_jacchus          ---------- ---------- ---------- ---------- ---------- ----------
Macaca_mulatta              ---------- ---------- ---------- ---------- ---------- ----------
Pongo_pygmaeus              ---------- ---------- ---------- ---------- ---------- ----------
Gorilla_gorilla             ---------- ---------- ---------- ---------- ---------- ----------
Homo_sapiens                ---------- ---------- ---------- ---------- ---------- ----------
Pan_troglodytes             ---------- ---------- ---------- ---------- ---------- ----------
Ochotona_princeps           ---------- ---------- ---------- ---------- ---------- ----------
Oryctolagus_cuniculus       ---------- ---------- ---------- ---------- ---------- ----------
Marmota_monax               ---------- ---------- ---------- ---------- ---------- ----------
Aplodantia_rufa             ---------- ---------- ---------- ---------- ---------- ----------
Spermophilus                ---------- ---------- ---------- ---------- ---------- ----------
Anomamorus                  ---------- ---------- ---------- ---------- ---------- ----------
Maxomys                     ---------- ---------- ---------- ---------- ---------- ----------
Rattus rattus               ---------- ---------- ---------- ---------- ---------- ----------
Rattus exulans              ---------- ---------- ---------- ---------- ---------- ----------
Rattus_norvegicus           ---------- ---------- ---------- ---------- ---------- ----------
Mus_musculus                ---------- ---------- ---------- ---------- ---------- ----------
Mus_spretus                 ---------- ---------- ---------- ---------- ---------- ----------
Mus_pahari                  ---------- ---------- ---------- ---------- ---------- ----------
Praomys                     ---------- ---------- ---------- ---------- ---------- ----------
Meriones                    ---------- ---------- ---------- ---------- ---------- ----------
Meriones_crassus            ---------- ---------- ---------- ---------- ---------- ----------
Acomys cahirinus            ---------- ---------- ---------- ---------- ---------- ----------
Lophuromys sikapusi         ---------- ---------- ---------- ---------- ---------- ----------
Mesocricetus_auratus        ---------- ---------- ---------- ---------- ---------- ----------
Oryzomys                    ---------- ---------- ---------- ---------- ---------- ----------
Trichys_fasciculata         ---------- ---------- ---------- ---------- ---------- ----------
Heterocephalus_glaber       ---------- ---------- ---------- ---------- ---------- ----------
Coendou_melanurus           ---------- ---------- ---------- ---------- ---------- ----------
Cavia_porcellus             ---------- ---------- ---------- ---------- ---------- ----------
Ctenomys_maulinus           ---------- ---------- ---------- ---------- ---------- ----------
Octodon_degus               ---------- ---------- ---------- ---------- ---------- ----------
Loxodonta_africana          ---------- ---------- ---------- ---------- ---------- ----------
Procavia_capensis           ---------- ---------- ---------- ---------- ---------- ----------
Echinops_telfairi           ---------- ---------- ---------- ---------- ---------- --GGGATGAG
Micropotamogale             ---------- ---------- ---------- ---------- ---------- ----------
Myotis_lucifugus            ---------- ---------- ---------- ---------- ---------- ----------
Canis_familiaris            ---------- ---------- ---------- ---------- ---------- ----------
Felis_catus                 ---------- ---------- ---------- ---------- ---------- ----------
Ovis_aries                  ---------- ---------- ---------- ---------- ---------- ----------
Equus_caballus              ---------- ---------- ---------- ---------- ---------- ----------
Tapirus_terrestris          ---------- ---------- ---------- ---------- ---------- ----------
Vicugna                     ---------- ---------- ---------- ---------- ---------- ----------
Sus_scrofa                  ---------- ---------- ---------- ---------- ---------- ----------
Bos_taurus                  AAATTCACTT CTGGGGGGAT ATACTGAAAT GTAGATTTAA GAGCTGCCGG TCGGGGGCGA
Tursiops_truncatus          ---------- ---------- ---------- ---------- ---------- ----------
Erinaceus                   ---------- ---------- ---------- ---------- ---------- ----------
Sorex_araneus
Neomys_anomalus             ---------- ---------- ---------- ---------- ---------- ----------
Choloepus_hoffmanni         ---------- ---------- ---------- ---------- ---------- ----------
Dasypus                     ---------- ---------- ---------- ---------- ---------- ----------
```

```
                         2821
Monodelphis                 AGTGCCAGGT ACTGGGGATA AAAAACCAAT AACCAAGCAG CCTGTGCCTT CCAAGAGCAG
Macropus                    TGTGCTAGGT GTTGGGGATA AAAAGTCAAT AAATA---AG TCTGTGTCCT CAAAGAG---
Microcebus_murinus          ---------- ---------- ---------- ---------- ---------- ----------
Tarsius_syrichta            ---------- ---------- ---------- ---------- ---------- ----------
Callithrix_jacchus          ---------- ---------- ---------- ---------- ---------- ----------
Macaca_mulatta              ---------- ---------- ---------- ---------- ---------- ----------
Pongo_pygmaeus              ---------- ---------- ---------- ---------- ---------- ----------
Gorilla_gorilla             ---------- ---------- ---------- ---------- ---------- ----------
Homo_sapiens                ---------- ---------- ---------- ---------- ---------- ----------
Pan_troglodytes             ---------- ---------- ---------- ---------- ---------- ----------
Ochotona_princeps           ---------- ---------- ---------- ---------- ---------- ----------
Oryctolagus_cuniculus       ---------- ---------- ---------- ---------- ---------- ----------
Marmota_monax               ---------- ---------- ---------- ---------- ---------- ----------
Aplodantia_rufa             ---------- ---------- ---------- ---------- ---------- ----------
Spermophilus                ---------- ---------- ---------- ---------- ---------- ----------
Anomamorus                  ---------- ---------- ---------- ---------- ---------- ----------
Maxomys                     ---------- ---------- ---------- ---------- ---------- ----------
Rattus rattus               ---------- ---------- ---------- ---------- ---------- ----------
Rattus exulans              ---------- ---------- ---------- ---------- ---------- ----------
Rattus_norvegicus           ---------- ---------- ---------- ---------- ---------- ----------
Mus_musculus                ---------- ---------- ---------- ---------- ---------- ----------
Mus_spretus                 ---------- ---------- ---------- ---------- ---------- ----------
Mus_pahari                  ---------- ---------- ---------- ---------- ---------- ----------
Praomys                     ---------- ---------- ---------- ---------- ---------- ----------
Meriones                    ---------- ---------- ---------- ---------- ---------- ----------
Meriones_crassus            ---------- ---------- ---------- ---------- ---------- ----------
Acomys cahirinus            ---------- ---------- ---------- ---------- ---------- ----------
Lophuromys sikapusi         ---------- ---------- ---------- ---------- ---------- ----------
Mesocricetus_auratus        ---------- ---------- ---------- ---------- ---------- ----------
Oryzomys                    ---------- ---------- ---------- ---------- ---------- ----------
Trichys_fasciculata         ---------- ---------- ---------- ---------- ---------- ----------
Heterocephalus_glaber       ---------- ---------- ---------- ---------- ---------- ----------
Coendou_melanurus           ---------- ---------- ---------- ---------- ---------- ----------
Cavia_porcellus             ---------- ---------- ---------- ---------- ---------- ----------
Ctenomys_maulinus           ---------- ---------- ---------- ---------- ---------- ----------
Octodon_degus               ---------- ---------- ---------- ---------- ---------- ----------
Loxodonta_africana          ---------- ---------- ---------- ---------- ---------- ----------
Procavia_capensis           ---------- ---------- ---------- ---------- ---------- ----------
Echinops_telfairi           ATCCCTCGTC AGGCTGAGCT GCCCCAGCGT GCTGCCACTG CTGCTGCGGG CAGCGATTGA
Micropotamogale             ---------- ---------- ---------- ---------- ---------- ----------
Myotis_lucifugus            ---------- ---------- ---------- ---------- ---------- ----------
Canis_familiaris            ---------- ---------- ---------- ---------- ---------- ----------
Felis_catus                 ---------- ---------- ---------- ---------- ---------- ----------
Ovis_aries                  ---------- ---------- ---------- ---------- ---------- ----------
Equus_caballus              ---------- ---------- ---------- ---------- ---------- ----------
Tapirus_terrestris          ---------- ---------- ---------- ---------- ---------- ----------
Vicugna                     ---------- ---------- ---------- ---------- ---------- ----------
Sus_scrofa                  ---------- ---------- ---------- ---------- ---------- ----------
Bos_taurus                  GGGGGGCGGG GGTGGAAATA GCATTTAAGT TCAGAAGTTG TATTTAGACT TTGGTTT---
Tursiops_truncatus          ---------- ---------- ---------- ---------- ---------- ----------
Erinaceus                   ---------- ---------- ---------- ---------- ---------- ----------
Sorex_araneus
Neomys_anomalus             ---------- ---------- ---------- ---------- ---------- -------TTA
Choloepus_hoffmanni         ---------- ---------- ---------- ---------- ---------- ----------
Dasypus                     ---------- ---------- ---------- ---------- ---------- ----------
```

```
                              2881
Monodelphis                ACATTTTATT GAACATGTAC ATAGAAAATT AAATACAAAA TA-------- ----------
Macropus                   ---------- ---CATGTAT GTAGAAAATT TAATACAGAA TA-------- ----------
Microcebus_murinus         ---------- ---------- --TGA----C CAAGAGATTG CT-------- ----------
Tarsius_syrichta           ---------- ---------- --GGAGAGGG CAATTCGGGA TT-------- ----------
Callithrix_jacchus         ---------- ---------- --TGAAG--- ----TCAGCA TT-------- ----------
Macaca_mulatta             ---------- ---------- --TGAAG--- ----CCATCC TT-------- ----------
Pongo_pygmaeus             ---------- ---------- --TGAAG--- ----CCATCC TT-------- ----------
Gorilla_gorilla            ---------- ---------- ---------- ---------- ---------- ----------
Homo_sapiens               ---------- ---------- --TGAAG--- ----CCATCT TT-------- ----------
Pan_troglodytes            ---------- ---------- --TGAAG--- ----CCATCT TT-------- ----------
Ochotona_princeps          ---------- ---------- --TAGAGGAC CAGGCAACCA TT-------- ----------
Oryctolagus_cuniculus      ---------- ---------- --TAGAGGAC CAGTTGAGTA TT-------- ----------
Marmota_monax              ---------- -------TAA AGGGATCGAT TAATTTAGAG TT-------- ----------
Aplodantia_rufa            ---------- -------TAA AGGGATCGAT TAATTTAGAA TT-------- ----------
Spermophilus               ---------- ---------- ---------- ---------- ---------- ----------
Anomamorus                 ---------- ---------- ---------- ---------- ---------- ----------
Maxomys                    ---------- ---------- AAGGACCAAT GGGTTCTGCA TT-------- ----------
Rattus rattus              ---------- ---------- AAGGGTCAAC GGGTTCTGCA TT-------- ----------
Rattus exulans             ---------- ---------- AAGGGTCAAC GGGTTCTGCA TT-------- ----------
Rattus_norvegicus          ---------- ---------- AAGGGTCAAC GGGTTCTGCA TT-------- ----------
Mus_musculus               ---------- ---------- AAGGGCCCAT GGGTTCTGCA TT-------- ----------
Mus_spretus                ---------- ---------- ---------- ---------- ---------- ----------
Mus_pahari                 ---------- ---------- ---------- ---------- ---------- ----------
Praomys                    ---------- ---------- AAGGGCCCAT GGGTTCTACA TT-------- ----------
Meriones                   ---------- ---------- AAGGACCAAC GGGTTCTGCA TT-------- ----------
Meriones_crassus           ---------- ---------- ---------- ---------- ---------- ----------
Acomys cahirinus           ---------- ---------- AAGGGCCAGT -------GCA TT-------- ----------
Lophuromys sikapusi        ---------- ---------- AAGGGCCAGT GGGTTCTGCA TT-------- ----------
Mesocricetus_auratus       ---------- ---------- AAGGGCCAGT GGATTCTGCA TT-------- ----------
Oryzomys                   ---------- ---------- AAAGGCCAAT GGGTTCTGCA TT-------- ----------
Trichys_fasciculata        ---------- TTTTTAAAAA ACAAAACAGT TAACCCAGCA TT-------- ----------
Heterocephalus_glaber      ---------- ---------A AAAGGCCAGT TAATTCA-CA TT-------- ----------
Coendou_melanurus          ---------- ---------A AAAGACCAGT TAATTCAGCA TT-------- ----------
Cavia_porcellus            ---------- ---------- AAAGACCAGT CAATTTAGCA TT-------- ----------
Ctenomys_maulinus          ---------- ------AAAG AAAGACTAGT TAATGCAGCA AT-------- ----------
Octodon_degus              ---------- ------AAAG AAAGACTACT TAATGCAGCA AT-------- ----------
Loxodonta_africana         ---------- -TTAAGACCA ATTTTTCTTT ---------- --TGGTTCCT -CATT-----
Procavia_capensis          ---------- ----AGACCA ATTGCTCATT ---------- ---------- ----------
Echinops_telfairi          CCCATCCCAT CCTCAGGCCT GGTCTCCATG CCAGGAGTTG CTTGGCTGCC CCACC-----
Micropotamogale            -----CCACT TCTCAGGCCA AGCACCCTT- ---------- ---------- ----------
Myotis_lucifugus           ---------- ---------- ---------- ---------- ---------- ----------
Canis_familiaris           ---------- -------TGC TTTTAAGGAC CAATTCCGCG TT-------- ----------
Felis_catus                ---------- -------CAG CGCGGGTCCT GATCTCGCTG TT-------- ----------
Ovis_aries                 ---------- ---------- ---------- ---------- ---------- ----------
Equus_caballus             ---------- ---------- ----AAGGAC CAAGTCAGCA TT-------- ----------
Tapirus_terrestris         ---------- ---------- ----AAGGAT CAAGTCAGCA TT-------- ----------
Vicugna                    ---------- ---------- ---AAAGGAC CAACTTGGCT AC-------- ----------
Sus_scrofa                 ---------- ---------- ----AGTGAC CAATTCCGCA TT-------- ----------
Bos_taurus                 ---------- ---------- ACTAAAGAAC CGATTCGGCA TT-------- ----------
Tursiops_truncatus         ---------- ---------- ---------- ------GGCA TT-------- ----------
Erinaceus                  ---------- ---------- -AGCGAGAGA GGTGCGCAGT ATTGTTTTAC TTCTCCAGAA
Sorex_araneus
Neomys_anomalus            ATTTATATTC CATTTTCCTC TCTTAAAGGA CCACTTCAGT GCTT------ -----CACAG
Choloepus_hoffmanni        --TTTTTAAG GAACAGTTCA GTATAAGGAA CAATTTAGTA TT-------- ----------
Dasypus                    ---------- ---------- ----AAGGAG CACCCGGGTA TT-------- ----------
```

```
                          2941
Monodelphis               -------CAT A-AAGGTGAT TTTCATCAGG AGGACATTAG CAACTGAGAC CTGTGTT-AT
Macropus                  -------TAT ACGAGGTGAT TTTCATCAGA AGGGTATTAG CAACTGGGAT CTGTATTGGG
Microcebus_murinus        -------TAT --GATA---- -ATTTGCTGA T--------- ---------- ----------
Tarsius_syrichta          -------TCC --GAGG---- ---------- ---------- ---------- ----------
Callithrix_jacchus        -------TCC --GAGA---- --------GA A--------- ---------- ----------
Macaca_mulatta            -------TCC --GAGA---- --------GA A--------- ---------- ----------
Pongo_pygmaeus            -------TCT --GAGA---- --------GA A--------- ---------- ----------
Gorilla_gorilla           ---------- ---------- ---------- ---------- ---------- ----------
Homo_sapiens              -------TCT --GAGA---- --------GA A--------- ---------- ----------
Pan_troglodytes           -------TCT --GAGA---- --------GA A--------- ---------- ----------
Ochotona_princeps         -------TCC --CAGA---- ---------- ---------- ---------- ----------
Oryctolagus_cuniculus     -------TCC --CAGA---- --------GA A--------- ---------- ----------
Marmota_monax             -------TTC --AAGA---- --------GA A--------- ---------- ----------
Aplodantia_rufa           -------ACC --AAAA---- --------GA A--------- ---------- ----------
Spermophilus              ---------- ---------- ---------- ---------- ---------- ----------
Anomamorus                ---------- ---------- ---------- ---------- ---------- ----------
Maxomys                   -------TCT AACAGA---- ---------- ---------- ---------- ----------
Rattus rattus             -------TCT AACAGA---- ---------- ---------- ---------- ----------
Rattus exulans            -------TCT AACAGA---- ---------- ---------- ---------- ----------
Rattus_norvegicus         -------TCT AACAGA---- ---------- ---------- ---------- ----------
Mus_musculus              -------TCT AACAGA---- ---------- ---------- ---------- ----------
Mus_spretus               ---------- ---------- ---------- ---------- ---------- ----------
Mus_pahari                ---------- ---------- ---------- ---------- ---------- ----------
Praomys                   -------TCT AACAGA---- ---------- ---------- ---------- ----------
Meriones                  -------TCT GACAGA---- ---------- ---------- ---------- ----------
Meriones_crassus          ---------- ---------- ---------- ---------- ---------- ----------
Acomys cahirinus          -------TCT G--------- ---------- ---------- ---------- ----------
Lophuromys sikapusi       -------TCT G--------- ---------- ---------- ---------- ----------
Mesocricetus_auratus      -------TTT GACAGAACTC TGCCCTGTGG AGTTTTTTTT TTGTTTG--- ----------
Oryzomys                  ---------- ---------- ---------- ---------- ---------- ----------
Trichys_fasciculata       -------TCT --GAGA---- --------GA A--------- ---------- ----------
Heterocephalus_glaber     -------TCT --GAGA---- --------GA A--------- ---------- ----------
Coendou_melanurus         -------T-- --GAGA---- --------GA A--------- ---------- ----------
Cavia_porcellus           -------TCT --GAGA---- --------GA A--------- ---------- ----------
Ctenomys_maulinus         -------TCT --GAGA---- --------AA A--------- ---------- ----------
Octodon_degus             -------TTT --GAGA---- --------AA A--------- ---------- ----------
Loxodonta_africana        -------TCC --GAAG---- -------AGA A--------- ---------- ----------
Procavia_capensis         -------TC- --GGAG---- -------AGA A--------- ---------- ----------
Echinops_telfairi         -AAGACACAC GCAGAG---- --GTGGCAGG A--------- ---------- ----------
Micropotamogale           -------TCC --TGTG---- -------GGA G--------- ---------- ----------
Myotis_lucifugus          -------CAC CTGGGG---- --------GG A--------- ---------- ----------
Canis_familiaris          -------T-- TCGAGA---- --------GA A--------- ---------- ----------
Felis_catus               ---------C ATGAGATTGA CCCCCTCGGA A--------- ---------- ----------
Ovis_aries                ---------- ---------- ---------- ---------- ---------- ----------
Equus_caballus            -------TCC --AAGA---- --------GA A--------- ---------- ----------
Tapirus_terrestris        -------TCC GAGAGA---- ---ACCCGAA A--------- ---------- ----------
Vicugna                   -------CCA CCGAGG---- --------GA G--------- ---------- ----------
Sus_scrofa                -------TCT --GAGG---- --------GA G--------- ---------- ----------
Bos_taurus                -------TCT --AAGG---- --------GA G--------- ---------- ----------
Tursiops_truncatus        -------TCC --GAGG---- --------GA G--------- ---------- ----------
Erinaceus                 AGTTCCCCTA CCCCGTATGT GAGGACCCAG GA-------- ---------- ----------
Sorex_araneus
Neomys_anomalus           AATCCCC--- ---------- ---------- ---------- ---------- ----------
Choloepus_hoffmanni       -------TCC --GAGA---- --------GA A--------- ---------- ----------
Dasypus                   -------TCT --GAGA---- --------GA A--------- ---------- ----------
```

```
                           3001
Monodelphis                   GAGATTAAGA TAGATTTCAG GTAGGAGGTA ACGTATGAGT TTACCTTTGA AGGAAGCT-A
Macropus                      GAGATGAAGC TAGGCTTCAT GTAGGAGATG ACATTTGAGC TGAACTTTGA AGAGAGCTAA
Microcebus_murinus            ---------- ---------- ---------- ---------- ---------- ----------
Tarsius_syrichta              ---------- ---------- ---------- ---------- ---------- ----------
Callithrix_jacchus            ---------- ---------- ---------- ---------- ---------- ----------
Macaca_mulatta                ---------- ---------- ---------- ---------- ---------- ----------
Pongo_pygmaeus                ---------- ---------- ---------- ---------- ---------- ----------
Gorilla_gorilla               ---------- ---------- ---------- ---------- ---------- ----------
Homo_sapiens                  ---------- ---------- ---------- ---------- ---------- ----------
Pan_troglodytes               ---------- ---------- ---------- ---------- ---------- ----------
Ochotona_princeps             ---------- ---------- ---------- ---------- ---------- ----------
Oryctolagus_cuniculus         ---------- ---------- ---------- ---------- ---------- ----------
Marmota_monax                 ---------- ---------- ---------- ---------- ---------- ----------
Aplodantia_rufa               ---------- ---------- ---------- ---------- ---------- ----------
Spermophilus                  ---------- ---------- ---------- ---------- ---------- ----------
Anomamorus                    ---------- ---------- ---------- ---------- ---------- ----------
Maxomys                       ---------- ---------- ---------- ---------- ---------- ----------
Rattus rattus                 ---------- ---------- ---------- ---------- ---------- ----------
Rattus exulans                ---------- ---------- ---------- ---------- ---------- ----------
Rattus_norvegicus             ---------- ---------- ---------- ---------- ---------- ----------
Mus_musculus                  ---------- ---------- ---------- ---------- ---------- ----------
Mus_spretus                   ---------- ---------- ---------- ---------- ---------- ----------
Mus_pahari                    ---------- ---------- ---------- ---------- ---------- ----------
Praomys                       ---------- ---------- ---------- ---------- ---------- ----------
Meriones                      ---------- ---------- ---------- ---------- ---------- ----------
Meriones_crassus              ---------- ---------- ---------- ---------- ---------- ----------
Acomys cahirinus              ---------- ---------- ---------- ---------- ---------- ----------
Lophuromys sikapusi           ---------- ---------- ---------- ---------- ---------- ----------
Mesocricetus_auratus          ---------- ---------- ---------- ---------- ---------- ----------
Oryzomys                      ---------- ---------- ---------- ---------- ---------- ----------
Trichys_fasciculata           ---------- ---------- ---------- ---------- ---------- ----------
Heterocephalus_glaber         ---------- ---------- ---------- ---------- ---------- ----------
Coendou_melanurus             ---------- ---------- ---------- ---------- ---------- ----------
Cavia_porcellus               ---------- ---------- ---------- ---------- ---------- ----------
Ctenomys_maulinus             ---------- ---------- ---------- ---------- ---------- ----------
Octodon_degus                 ---------- ---------- ---------- ---------- ---------- ----------
Loxodonta_africana            ---------- ---------- ---------- ---------- ---------- ----------
Procavia_capensis             ---------- ---------- ---------- ---------- ---------- ----------
Echinops_telfairi             ---------- ---------- ---------- ---------- ---------- ----------
Micropotamogale               ---------- ---------- ---------- ---------- ---------- ----------
Myotis_lucifugus              ---------- ---------- ---------- ---------- ---------- ----------
Canis_familiaris              ---------- ---------- ---------- ---------- ---------- ----------
Felis_catus                   ---------- ---------- ---------- ---------- ---------- ----------
Ovis_aries                    ---------- ---------- ---------- ---------- ---------- ----------
Equus_caballus                ---------- ---------- ---------- ---------- ---------- ----------
Tapirus_terrestris            ---------- ---------- ---------- ---------- ---------- ----------
Vicugna                       ---------- ---------- ---------- ---------- ---------- ----------
Sus_scrofa                    ---------- ---------- ---------- ---------- ---------- ----------
Bos_taurus                    ---------- ---------- ---------- ---------- ---------- ----------
Tursiops_truncatus            ---------- ---------- ---------- ---------- ---------- ----------
Erinaceus                     ---------- ---------- ---------- ---------- ---------- ----------
Sorex_araneus
Neomys_anomalus               ---------- ---------- ---------- ---------- ---------- --TTCACAGT
Choloepus_hoffmanni           ---------- ---------- ---------- ---------- ---------- ----------
Dasypus                       ---------- ---------- ---------- ---------- ---------- ----------
```

```
                        3061
Monodelphis                AAAATTCCAT GAGATAAGGT GAGGAGGGAG GGC-AAACCA GCTATAAGGA AAACCCTTTA
Macropus                   AAAATTCCAT GAGGTGAGGT GAGGAGGGAG GGCTTTACTG ACTAGGAAGG GAATCCT---
Microcebus_murinus         ---------- ---------- ---------- ---------- ---------- ----------
Tarsius_syrichta           ---------- ---------- ---------- ---------- ---------- ----------
Callithrix_jacchus         ---------- ---------- ---------- ---------- ---------- ----------
Macaca_mulatta             ---------- ---------- ---------- ---------- ---------- ----------
Pongo_pygmaeus             ---------- ---------- ---------- ---------- ---------- ----------
Gorilla_gorilla            ---------- ---------- ---------- ---------- ---------- ----------
Homo_sapiens               ---------- ---------- ---------- ---------- ---------- ----------
Pan_troglodytes            ---------- ---------- ---------- ---------- ---------- ----------
Ochotona_princeps          ---------- ---------- ---------- ---------- ---------- ----------
Oryctolagus_cuniculus      ---------- ---------- ---------- ---------- ---------- ----------
Marmota_monax              ---------- ---------- ---------- ---------- ---------- ----------
Aplodantia_rufa            ---------- ---------- ---------- ---------- ---------- ----------
Spermophilus               ---------- ---------- ---------- ---------- ---------- ----------
Anomamorus                 ---------- ---------- ---------- ---------- ---------- ----------
Maxomys                    ---------- ---------- ---------- ---------- ---------- ----------
Rattus rattus              ---------- ---------- ---------- ---------- ---------- ----------
Rattus exulans             ---------- ---------- ---------- ---------- ---------- ----------
Rattus_norvegicus          ---------- ---------- ---------- ---------- ---------- ----------
Mus_musculus               ---------- ---------- ---------- ---------- ---------- ----------
Mus_spretus                ---------- ---------- ---------- ---------- ---------- ----------
Mus_pahari                 ---------- ---------- ---------- ---------- ---------- ----------
Praomys                    ---------- ---------- ---------- ---------- ---------- ----------
Meriones                   ---------- ---------- ---------- ---------- ---------- ----------
Meriones_crassus           ---------- ---------- ---------- ---------- ---------- ----------
Acomys cahirinus           ---------- ---------- ---------- ---------- ---------- ----------
Lophuromys sikapusi        ---------- ---------- ---------- ---------- ---------- ----------
Mesocricetus_auratus       ---------- ---------- ---------- ---------- ---------- ----------
Oryzomys                   ---------- ---------- ---------- ---------- ---------- ----------
Trichys_fasciculata        ---------- ---------- ---------- ---------- ---------- ----------
Heterocephalus_glaber      ---------- ---------- ---------- ---------- ---------- ----------
Coendou_melanurus          ---------- ---------- ---------- ---------- ---------- ----------
Cavia_porcellus            ---------- ---------- ---------- ---------- ---------- ----------
Ctenomys_maulinus          ---------- ---------- ---------- ---------- ---------- ----------
Octodon_degus              ---------- ---------- ---------- ---------- ---------- ----------
Loxodonta_africana         ---------- ---------- ---------- ---------- ---------- ----------
Procavia_capensis          ---------- ---------- ---------- ---------- ---------- ----------
Echinops_telfairi          ---------- ---------- ---------- ---------- ---------- ----------
Micropotamogale            ---------- ---------- ---------- ---------- ---------- ----------
Myotis_lucifugus           ---------- ---------- ---------- ---------- ---------- ----------
Canis_familiaris           ---------- ---------- ---------- ---------- ---------- ----------
Felis_catus                ---------- ---------- ---------- ---------- ---------- ----------
Ovis_aries                 ---------- ---------- ---------- ---------- ---------- ----------
Equus_caballus             ---------- ---------- ---------- ---------- ---------- ----------
Tapirus_terrestris         ---------- ---------- ---------- ---------- ---------- ----------
Vicugna                    ---------- ---------- ---------- ---------- ---------- ----------
Sus_scrofa                 ---------- ---------- ---------- ---------- ---------- ----------
Bos_taurus                 ---------- ---------- ---------- ---------- ---------- ----------
Tursiops_truncatus         ---------- ---------- ---------- ---------- ---------- ----------
Erinaceus                  ---------- ---------- ---------- ---------- ---------- ----------
Sorex_araneus
Neomys_anomalus            TGTTTTCTTT TCTTTTTGTT TTTTCTTTGG GGGGGAGTCA CACCCGGCAG TGCTCAGGGG
Choloepus_hoffmanni        ---------- ---------- ---------- ---------- ---------- ----------
Dasypus                    ---------- ---------- ---------- ---------- ---------- ----------
```

```
                         3121
Monodelphis                 AAAATTCAGT TACCCTTTCT ACCTGTGTGA CTTTAGGCAA ATCTCTTAAT C--TTCCAAG
Macropus                    AAGTCTCAGT TACCCTTTGT ACCTGTGTGA CTTTGGGTAA GTCTTTTAAC CTATGTTAAG
Microcebus_murinus          ---------- ---------- ---------- ---------- ---------- ----------
Tarsius_syrichta            ---------- ---------- ---------- ---------- ---------- ----------
Callithrix_jacchus          ---------- ---------- ---------- ---------- ---------- ----------
Macaca_mulatta              ---------- ---------- ---------- ---------- ---------- ----------
Pongo_pygmaeus              ---------- ---------- ---------- ---------- ---------- ----------
Gorilla_gorilla             ---------- ---------- ---------- ---------- ---------- ----------
Homo_sapiens                ---------- ---------- ---------- ---------- ---------- ----------
Pan_troglodytes             ---------- ---------- ---------- ---------- ---------- ----------
Ochotona_princeps           ---------- ---------- ---------- ---------- ---------- ----------
Oryctolagus_cuniculus       ---------- ---------- ---------- ---------- ---------- ----------
Marmota_monax               ---------- ---------- ---------- ---------- ---------- ----------
Aplodantia_rufa             ---------- ---------- ---------- ---------- ---------- ----------
Spermophilus                ---------- ---------- ---------- ---------- ---------- ----------
Anomamorus                  ---------- ---------- ---------- ---------- ---------- ----------
Maxomys                     ---------- ---------- ---------- ---------- ---------- ----------
Rattus rattus               ---------- ---------- ---------- ---------- ---------- ----------
Rattus exulans              ---------- ---------- ---------- ---------- ---------- ----------
Rattus_norvegicus           ---------- ---------- ---------- ---------- ---------- ----------
Mus_musculus                ---------- ---------- ---------- ---------- ---------- ----------
Mus_spretus                 ---------- ---------- ---------- ---------- ---------- ----------
Mus_pahari                  ---------- ---------- ---------- ---------- ---------- ----------
Praomys                     ---------- ---------- ---------- ---------- ---------- ----------
Meriones                    ---------- ---------- ---------- ---------- ---------- ----------
Meriones_crassus            ---------- ---------- ---------- ---------- ---------- ----------
Acomys cahirinus            ---------- ---------- ---------- ---------- ---------- ----------
Lophuromys sikapusi         ---------- ---------- ---------- ---------- ---------- ----------
Mesocricetus_auratus        ---------- ---------- ---------- ---------- ---------- ----------
Oryzomys                    ---------- ---------- ---------- ---------- ---------- ----------
Trichys_fasciculata         ---------- ---------- ---------- ---------- ---------- ----------
Heterocephalus_glaber       ---------- ---------- ---------- ---------- ---------- ----------
Coendou_melanurus           ---------- ---------- ---------- ---------- ---------- ----------
Cavia_porcellus             ---------- ---------- ---------- ---------- ---------- ----------
Ctenomys_maulinus           ---------- ---------- ---------- ---------- ---------- ----------
Octodon_degus               ---------- ---------- ---------- ---------- ---------- ----------
Loxodonta_africana          ---------- ---------- ---------- ---------- ---------- ----------
Procavia_capensis           ---------- ---------- ---------- ---------- ---------- ----------
Echinops_telfairi           ---------- ---------- ---------- ---------- ---------- ----------
Micropotamogale             ---------- ---------- ---------- ---------- ---------- ----------
Myotis_lucifugus            ---------- ---------- ---------- ---------- ---------- ----------
Canis_familiaris            ---------- ---------- ---------- ---------- ---------- ----------
Felis_catus                 ---------- ---------- ---------- ---------- ---------- ----------
Ovis_aries                  ---------- ---------- ---------- ---------- ---------- ----------
Equus_caballus              ---------- ---------- ---------- ---------- ---------- ----------
Tapirus_terrestris          ---------- ---------- ---------- ---------- ---------- ----------
Vicugna                     ---------- ---------- ---------- ---------- ---------- ----------
Sus_scrofa                  ---------- ---------- ---------- ---------- ---------- ----------
Bos_taurus                  ---------- ---------- ---------- ---------- ---------- ----------
Tursiops_truncatus          ---------- ---------- ---------- ---------- ---------- ----------
Erinaceus                   ---------- ---------- ---------- ---------- ---------- ----------
Sorex_araneus
Neomys_anomalus             TTATTCCTGG CTCTGTGCTC AGGAATCAAC TCCTGGCGGT GCACAGGGGA CCATATGGGA
Choloepus_hoffmanni         ---------- ---------- ---------- ---------- ---------- ----------
Dasypus                     ---------- ---------- ---------- ---------- ---------- ----------
```

```
                              3181
Monodelphis                   TCTCA---GT TTCTTCATCT GCAAAATGAT GGGATTGAAC TAGATCCCTC TGAGGTAGCT
Macropus                      CATGAAGTGT TTCTTCATCT GT-------- ---------- --GATCCCTC TGAAGTCGCT
Microcebus_murinus            ---------- ---------- ---------- ---------- -------ATA TTGTACACTA
Tarsius_syrichta              ---------- ---------- ---------- ---------- ---------- ----------
Callithrix_jacchus            ---------- ---------- ---------- ---------- -------CCC -GAAGC----
Macaca_mulatta                ---------- ---------- ---------- ---------- -------CCC -GAAGC----
Pongo_pygmaeus                ---------- ---------- ---------- ---------- -------CCC -GAAGC----
Gorilla_gorilla               ---------- ---------- ---------- ---------- ---------- ----------
Homo_sapiens                  ---------- ---------- ---------- ---------- -------CCC -GAAGC----
Pan_troglodytes               ---------- ---------- ---------- ---------- -------CTC -GAAGC----
Ochotona_princeps             ---------- ---------- ---------- ---------- ---------- ----------
Oryctolagus_cuniculus         ---------- ---------- ---------- ---------- -------CCT -GAAGC----
Marmota_monax                 ---------- ---------- ---------- ---------- -------CCG -GAAGCGCTT
Aplodantia_rufa               ---------- ---------- ---------- ---------- -------CTG -GAAGC--TA
Spermophilus                  ---------- ---------- ---------- ---------- ---------- ----------
Anomamorus                    ---------- ---------- ---------- ---------- ---------- ----------
Maxomys                       ---------- ---------- ---------- ---------- -------ACC CTACACTGTT
Rattus rattus                 ---------- ---------- ---------- ---------- -------ACT CTCCACTGTT
Rattus exulans                ---------- ---------- ---------- ---------- -------ACT CTACACTGTT
Rattus_norvegicus             ---------- ---------- ---------- ---------- -------ACT CTACACTGTT
Mus_musculus                  ---------- ---------- ---------- ---------- -------ACC GTATACTGTT
Mus_spretus                   ---------- ---------- ---------- ---------- ---------- ----------
Mus_pahari                    ---------- ---------- ---------- ---------- ---------- ----------
Praomys                       ---------- ---------- ---------- ---------- -------ACC TTACACCGTT
Meriones                      ---------- ---------- ---------- ---------- ---------- ----------
Meriones_crassus              ---------- ---------- ---------- ---------- ---------- ----------
Acomys cahirinus              ---------- ---------- ---------- ---------- ---------- -------GTT
Lophuromys sikapusi           ---------- ---------- ---------- ---------- ---------- -------GGG
Mesocricetus_auratus          ---------- ---------- ---------- ---------- ---------- --------TT
Oryzomys                      ---------- ---------- ---------- ---------T CTGACAGAAC CTACATTCTT
Trichys_fasciculata           ---------- ---------- ---------- ---------- -------TCC -GAAGC----
Heterocephalus_glaber         ---------- ---------- ---------- ---------- -------CTC -AAAGGTTTT
Coendou_melanurus             ---------- ---------- ---------- ---------- -------CCC -AAAGC----
Cavia_porcellus               ---------- ---------- ---------- ---------- -------CTC -AGAGC----
Ctenomys_maulinus             ---------- ---------- ---------- ---------- -------CCA -AAAGC----
Octodon_degus                 ---------- ---------- ---------- ---------- -------CCA GAAAGC----
Loxodonta_africana            ---------- ---------- ---------- ---------- -------CCC -AAAGC----
Procavia_capensis             ---------- ---------- ---------- ---------- -------CCA ----------
Echinops_telfairi             ---------- ---------- ---------- ---------- --CCCCCTGC AATGGC----
Micropotamogale               ---------- ---------- ---------- ---------- -------CCC -AACGC----
Myotis_lucifugus              ---------- ---------- ---------- ---------- -------CCC ----------
Canis_familiaris              ---------- ---------- ---------- ---------- -------CCC -CAAAC----
Felis_catus                   ---------- ---------- ---------- ---------- -------ATC -AAAGC----
Ovis_aries                    ---------- ---------- ---------- ---------- ---------- ----------
Equus_caballus                ---------- ---------- ---------- ---------- -------CCC -CAAGC----
Tapirus_terrestris            ---------- ---------- ---------- ---------- -------CCC TTTTTCCAGA
Vicugna                       ---------- ---------- ---------- ---------- -------CCC -AAAGC----
Sus_scrofa                    ---------- ---------- ---------- ---------- -------CCC -TAAGC----
Bos_taurus                    ---------- ---------- ---------- ---------- -------TGC -TAAGC----
Tursiops_truncatus            ---------- ---------- ---------- ---------- -------CCC -TAAGC----
Erinaceus                     ---------- ---------- ---------- ---------- ---------C TTGAACTCAA
Sorex_araneus
Neomys_anomalus               TGCAGGGGCT CGAACCCGGG TAGGCCGCAT GCAAGGCAAA AGCCCTACCC ACTAGACTAT
Choloepus_hoffmanni           ---------- ---------- ---------- ---------- -------CCC -AAAGC----
Dasypus                       ---------- ---------- ---------- ---------- -------CCC -AAAGC----
```

```
                        3241
Monodelphis             TCCATTTCTG TGTTCCTACG TTCGTCAATA TAAATTAAAT TTTAAGCTTT CATGTAAGAA
Macropus                TTCACCTTCA GATC----TG TTCGTCATCA TAAATTAAAT CCTAAGCTTT CTTATAAGAA
Microcebus_murinus      AATTTGATTA TTGT------ ---------- ---------- ---------- ----------
Tarsius_syrichta        ---------- ---------- ---------- ---------- ---------- ----------
Callithrix_jacchus      -------TGT TTCT------ ---------- ---------- ---------- ----------
Macaca_mulatta          -------TGT TTTT------ ---------- ---------- ---------- ----------
Pongo_pygmaeus          -------TGT TTTT------ ---------- ---------- ---------- ----------
Gorilla_gorilla         ---------- ---------- ---------- ---------- ---------- ----------
Homo_sapiens            -------TGT TTTT------ ---------- ---------- ---------- ----------
Pan_troglodytes         -------TGT TTTT------ ---------- ---------- ---------- ----------
Ochotona_princeps       -CAGTCTGTG TTTT------ ---------- ---------- ---------- ----------
Oryctolagus_cuniculus   -------AGT GTTT------ ---------- ---------- ---------- ----------
Marmota_monax           TTATTTTATT TTTT------ ---------- ---------- ---------- ----------
Aplodantia_rufa         TTATTTTATT TTAT------ ---------- ---------- ---------- ----------
Spermophilus            ---------- ---------- ---------- ---------- ---------- ----------
Anomamorus              ---------- ---------- ---------- ---------- ---------- ----------
Maxomys                 TGGGGGCGGT TTGT------ ---------- ---------- ---------- ----------
Rattus rattus           TTTTGGGGGT GGGGGTGGAG GTGGGGGTTG ---------- ---------- ----------
Rattus exulans          TTTGGGGGGT GGGGGTGGGG GTTG------ ---------- ---------- ----------
Rattus_norvegicus       TTTTGGGGGT GGGGGTGGGG GTTGTTTT-- ---------- ---------- ----------
Mus_musculus            TTTTTGGTTT TTGC------ ---------- ---------- ---------- ----------
Mus_spretus             ---------- ---------- ---------- ---------- ---------- ----------
Mus_pahari              ---------- ---------- ---------- ---------- ---------- ----------
Praomys                 TTTGTTGTTG TTGT------ ---------- ---------- ---------- ----------
Meriones                ---------- ---------- ---------- ---------- ---------- ----------
Meriones_crassus        ---------- ---------- ---------- ---------- ---------- ----------
Acomys cahirinus        TTGTTTTGTT TTGTTTTGTT TTGTTTTGTT TTT------- ---------- ----------
Lophuromys sikapusi     TTTTTTGGGG GGTGG--TGG TGGTGTTGTT TTTGCTTTTT G--------- ----------
Mesocricetus_auratus    TTTGAGTTTT TGGTTTTTTT TGTTT----- ---------- ---------- ----------
Oryzomys                TTTTCCC--- ---------- ---------- ---------- ---------- ----------
Trichys_fasciculata     -------TTT TTTG------ ---------- ---------- ---------- ----------
Heterocephalus_glaber   TTTTTCTTTC TTTT------ ---------- ---------- ---------- ----------
Coendou_melanurus       ---TTTTTTT TTCC------ ---------- ---------- ---------- ----------
Cavia_porcellus         --------TT TTTC------ ---------- ---------- ---------- ----------
Ctenomys_maulinus       -----TTTTT TCTT------ ---------- ---------- ---------- ----------
Octodon_degus           -----TTTTT TCTT------ ---------- ---------- ---------- ----------
Loxodonta_africana      ---------- -TGT------ ---------- ---------- ---------- ----------
Procavia_capensis       ---------- ---------- ---------- ---------- ---------- ----------
Echinops_telfairi       ---------- -TGC------ ---------- ---------- ---------- ----------
Micropotamogale         ---------- -TGT------ ---------- ---------- ---------- ----------
Myotis_lucifugus        ---------- ---------- ---------- ---------- ---------- ----------
Canis_familiaris        --------TG CTTT------ ---------- ---------- ---------- ----------
Felis_catus             ---------- ---------- ---------- ---------- ---------- ----------
Ovis_aries              ---------- ---------- ---------- ---------- ---------- ----------
Equus_caballus          --------TC TTTT------ ---------- ---------- ---------- ----------
Tapirus_terrestris      GCGGCTGTGC GGGTGTCGGG CGCCCTCTCT TCATTGCTTT GTGTAGCACT TACCCTGGA-
Vicugna                 ---------- TGTC------ ---------- ---------- ---------- ----------
Sus_scrofa              -------T-C TTTT------ ---------- ---------- ---------- ----------
Bos_taurus              --------TA TTTT------ ---------- ---------- ---------- ----------
Tursiops_truncatus      --------TA TTTT------ ---------- ---------- ---------- ----------
Erinaceus               ACCCTTGCAC ACTTGC---- ---------- ---------- ---------- ----------
Sorex_araneus
Neomys_anomalus         CACTCCAGTC CGGACAGTTG TATTTATCTA GAGACTGTGT GTGTGTGTGT GTGTGTGTGT
Choloepus_hoffmanni     --------TA TTTT------ ---------- ---------- ---------- ----------
Dasypus                 ---------- TGTT------ ---------- ---------- ---------- ----------
```

```
                              3301
Monodelphis                   TTAAATATCT CCCCAATACT TAATAGAAGC TACTTCAGTA AAGGGCCTAG GTGCTGCTAA
Macropus                      TTAAACTTCT CCACACTAAT TCACAGTAGC TATTTCAGTA AAAAGCCTAG GTGCTGCTAA
Microcebus_murinus            ---------- ---------- ---------- ----CTCTTT CAAA------ ----------
Tarsius_syrichta              ---------- ---------- ---------- ---------- GAGGCTGAGT GT--------
Callithrix_jacchus            ---------- ---------- ---------- ----TCT--A GAGAGGG--T GTGC------
Macaca_mulatta                ---------- ---------- ---------- ----TCT--A GGGAGGC--T GTGA------
Pongo_pygmaeus                ---------- ---------- ---------- ----TCT--A GAGAAGC--T GTGA------
Gorilla_gorilla               ---------- ---------- ---------- ---------- ---------- ----------
Homo_sapiens                  ---------- ---------- ---------- ----TCT--A GAGAAGC--T GTGA------
Pan_troglodytes               ---------- ---------- ---------- ----TCT--A GAGAAGC--T GTTC------
Ochotona_princeps             ---------- ---------- ---------- ----TCT--A CAGAGGCTGT GAGG------
Oryctolagus_cuniculus         ---------- ---------- ---------- ----TCT--A GAGAGGC--T ATGA------
Marmota_monax                 ---------- ---------- ---------- -----ACT-A GAGAGGCCAG GGGA------
Aplodantia_rufa               ---------- ---------- ---------- --TTTTTCTA GAGAGACCAA GAAA------
Spermophilus                  ---------- ---------- ---------- ---------- ---------- ----------
Anomamorus                    ---------- ---------- ---------- ---------- ---------- ----------
Maxomys                       ---------- ---------- --------TT TTTTCCTTCA GAAGAGTGAA CC--------
Rattus rattus                 ---------- ---------- ---------- ---------- ---------- ----------
Rattus exulans                ---------- ---------- ---------- ---------- ---------- ----------
Rattus_norvegicus             ---------- ---------- ---------- -TTTCCTTCA GAAGAGTGAA CC--------
Mus_musculus                  ---------- ---------- ---------T TTTTCCTTCA GAGGAGTAAA CC--------
Mus_spretus                   ---------- ---------- ---------- ---------- ---------- ----------
Mus_pahari                    ---------- ---------- ---------- ---------- ---------- ----------
Praomys                       ---------- ---------T GTTGTTTGCT TTTTCCGTCA GAGGAGAGAA GC--------
Meriones                      ---------- ---------- ---------- ---------- ---------- ----------
Meriones_crassus              ---------- ---------- ---------- ---------- ---------- ----------
Acomys cahirinus              ---------- ---------- ---------- ----CCTCCA AAGGAATGAA GA--------
Lophuromys sikapusi           ---------- ---------- ---------- ---TCCTCCA GAGGAGTGAA CA--------
Mesocricetus_auratus          ---------- ---GTTTGTT TGTTTGTTTT GTTTTCTCCA GAAGAGTGA- ----------
Oryzomys                      ---------- ---------- ---------- ----CCTTCA GAGTAGTGA- ----------
Trichys_fasciculata           ---------- ---------- ---------- ----CCAAC- ---------- ----------
Heterocephalus_glaber         ---------- ---------- ---------- ----TCTTCT TAGAGGCCAA GTGA------
Coendou_melanurus             ---------- ---------- ---------- ----CTC--T TAGAGGCCAA GTGA------
Cavia_porcellus               ---------- ---------- ---------- ---CCCCCTT -AGAGGCCAA GTGA------
Ctenomys_maulinus             ---------- ---------- ---------- ----TCCCCT GAGAGGCTAA ATGA------
Octodon_degus                 ---------- ---------- ---------- ----CCCCCT CGCAGGCTAA ACGA------
Loxodonta_africana            ---------- ---------- ---------- ----TTTCTA GGGAGGCT-- --GA------
Procavia_capensis             ---------- ---------- ---------- ------ACTA GAGAGGGT-- --GC------
Echinops_telfairi             ---------- ---------- ---------- ----CCACTA GTGATGCT-- --CC------
Micropotamogale               ---------- ---------- ---------- ---TTCCCA- ---AGG-T-- --GC------
Myotis_lucifugus              ---------- ---------- ---------- -----CACCT CGGGGGGTGG GGGCGGTCTC
Canis_familiaris              ---------- ---------- ---------- ----CCT--A GAATGGCTGT CCGG------
Felis_catus                   ---------- ---------- ---------- ---------C TGGATCCTGC TTCA------
Ovis_aries                    ---------- ---------- ---------- ---------- ---------- ----------
Equus_caballus                ---------- ---------- ---------- ----TCT--A GAGAGGCTGG GAG-------
Tapirus_terrestris            ---------- ---------- ---------- ---------- ---------- ----------
Vicugna                       ---------- ---------- ---------- ----TTCCTA GCGAGCCTGT GAGA------
Sus_scrofa                    ---------- ---------- ---------- ----TCT--A GAGAGGCAGT GAGA------
Bos_taurus                    ---------- ---------- ---------- ----TCT--A GCGAGGCTGT GCGA------
Tursiops_truncatus            ---------- ---------- ---------- ----TCT--A GCGAGGCTGT GCGA------
Erinaceus                     ---------- ---------- ---TCTCTAC TGGGTTT--- GCCAGGCTCT TTTTTTTTTT
Sorex_araneus
Neomys_anomalus               GTGTGTGTGT GTGTGT---- ---------- ---------- ---------- ----------
Choloepus_hoffmanni           ---------- ---------- ---------- ----GANNNN N--------- ----------
Dasypus                       ---------- ---------- ---------- ----TCTCTA GAGAGGCTGG GGGA------
```

```
                           3361
Monodelphis                TG-------- ---------- ---------- ---------- ---------- ---------A
Macropus                   TG-------- ---------- ---------- ---------- ---------- ---------A
Microcebus_murinus         ---------- ---------- ---------- ---------- ---------- ----------
Tarsius_syrichta           ---------- ---------- ---------- ---------- ---------- ----------
Callithrix_jacchus         ---------- ---------- ---------- ---------- ---------- ---------G
Macaca_mulatta             ---------- ---------- ---------- ---------- ---------- ---------G
Pongo_pygmaeus             ---------- ---------- ---------- ---------- ---------- ---------G
Gorilla_gorilla            ---------- ---------- ---------- ---------- ---------- ----------
Homo_sapiens               ---------- ---------- ---------- ---------- ---------- ---------G
Pan_troglodytes            ---------- ---------- ---------- ---------- ---------- ----------
Ochotona_princeps          ---------- ---------- ---------- ---------- ---------- ---------G
Oryctolagus_cuniculus      ---------- ---------- ---------- ---------- ---------- ---------G
Marmota_monax              ---------- ---------- ---------- ---------- ---------- ---------A
Aplodantia_rufa            ---------- ---------- ---------- ---------- ---------- ---------G
Spermophilus               ---------- ---------- ---------- ---------- ---------- ----------
Anomamorus                 ---------- ---------- ---------- ---------- ---------- ----------
Maxomys                    ---------- ---------- ---------- ---------- ---------- ---------A
Rattus rattus              ---------- ---------- ---------- ---------- ---------- ----------
Rattus exulans             ---------- ---------- ---------- ---------- ---------- ----------
Rattus_norvegicus          ---------- ---------- ---------- ---------- ---------- ---------A
Mus_musculus               ---------- ---------- ---------- ---------- ---------- ---------A
Mus_spretus                ---------- ---------- ---------- ---------- ---------- ----------
Mus_pahari                 ---------- ---------- ---------- ---------- ---------- ----------
Praomys                    ---------- ---------- ---------- ---------- ---------- ---------A
Meriones                   ---------- -ACCCGCCAC TGTATTCTCC CCTCCCCCGC ATCTCTGGAA GAGTGTGCAA
Meriones_crassus           ---------- ---------- ---------- ---------- ---------- ----------
Acomys cahirinus           ---------- ---------- ---------- ---------- ---------- ---------A
Lophuromys sikapusi        ---------- ---------- ---------- ---------- ---------- ---------A
Mesocricetus_auratus       ---------- ---------- ---------- ---------- ---------- ------GCAG
Oryzomys                   ---------- ---------- ---------- ---------- ---------- ------ACAA
Trichys_fasciculata        ---------- ---------- ---------- ---------- ---------- ----------
Heterocephalus_glaber      ---------- ---------- ---------- ---------- ---------- ---------G
Coendou_melanurus          ---------- ---------- ---------- ---------- ---------- ---------G
Cavia_porcellus            ---------- ---------- ---------- ---------- ---------- ---------G
Ctenomys_maulinus          ---------- ---------- ---------- ---------- ---------- ---------G
Octodon_degus              ---------- ---------- ---------- ---------- ---------- ---------G
Loxodonta_africana         ---------- ---------- ---------- ---------- ---------- ---------G
Procavia_capensis          ---------- ---------- ---------- ---------- ---------- ---------G
Echinops_telfairi          ---------- ---------- ---------- ---------- -------ACT TGAAGGGAAG
Micropotamogale            ---------- ---------- ---------- ---------- ---------- --------AG
Myotis_lucifugus           TGATT----- ---------- ---------- ---------- ---------- ---------A
Canis_familiaris           ---------- ---------- ---------- ---------- ---------- ---------A
Felis_catus                ---------- ---------- ---------- ---------- ---------- ---------A
Ovis_aries                 ---------- ---------- ---------- ---------- ---------- ----------
Equus_caballus             ---------- ---------- ---------- ---------- ---------- ----------
Tapirus_terrestris         ---------- ---------- ---------- ---------- ---------- ----------
Vicugna                    ---------- ---------- ---------- ---------- ---------- ---------G
Sus_scrofa                 ---------- ---------- ---------- ---------- ---------- ---------G
Bos_taurus                 ---------- ---------- ---------- ---------- ---------- ---------C
Tursiops_truncatus         ---------- ---------- ---------- ---------- ---------- ---------G
Erinaceus                  TTTTTTTTTT TTTTTTTAGT TCTGTCCAAG AACCCTCAGC TATTTTTGTA AAGAGGGCTA
Sorex_araneus
Neomys_anomalus            ---------- ---------- ---------- ---------- ---------- ----------
Choloepus_hoffmanni        ---------- ---------- ---------- ---------- ---------- ----------
Dasypus                    ---------- ---------- ---------- ---------- ---------- ---------G
```

```
                          3421
Monodelphis               GTTTTGCAAT GGGAATTATC TTTTCATTCT CT-TTGTTGT ---AATACTT -CCCTGGGGG
Macropus                  GTTTTGCCAT GAGAATCATC TTTTCATTCT CT-TTGTTGT ---AGTACTT -CCCCGGGGG
Microcebus_murinus        ---------C CAGTTTTACC TTTCTGTTC- TTATGTGTGA ---A-GGC-- ----------
Tarsius_syrichta          GGATGGGC-- -------TGC TTTCCCTCG- ---CTTTGTG T---CGCTCC CACCCGGGGG
Callithrix_jacchus        TGTGAAT--C GGGCAACTTC TTTTCACTC- GT-TTTGTGC ---GGCACTC ACCCT---GG
Macaca_mulatta            TGTGTTT--C GGGCCACTTG TTTTCACTC- CT-TTTGTGC ---AGCACTC ACCCT---GG
Pongo_pygmaeus            TGTGTAT--C GGGCGACTTC TGTTCACTC- CT-TTTCTGC ---GGCACTC ACCCT---GG
Gorilla_gorilla           ---------- ---------- ---------- ---------- ---------- ----------
Homo_sapiens              TGTGTAT--T GGGCGACTTC TGTTCACTC- CT-TTTCTGC ---AGCACTC ACCCT---GG
Pan_troglodytes           ---------- ---------- ---------- -------TGC ---AGCACTC ACCCT---GG
Ochotona_princeps         TGCATCT--C GAATCTTCTT TTTAAGGCC- CT--TTGTGT ---AGCGTTC ACCTT---GG
Oryctolagus_cuniculus     TGTGCGC--C TCAAGTCTGC T--------- ----TTGTGT ---AGCATTC ACCCT---GG
Marmota_monax             CGCGTGT--- CTGGCGTTTT CCT-CCCGC- TCCTTTGTGC ---AGCACTA CCCCA---GG
Aplodantia_rufa           CTTGTGT--- CTGGCG-TTC TCTTCTCGC- TCCTTTGCGA ---AGCACTC ACC-TATA--
Spermophilus              ---------- ---------- ---------- ---------- ---------- ----------
Anomamorus                ---------- ---------- ---------- ---------- ---------- ----------
Maxomys                   GAGACCTCAC GGCTGACTTT TCTTCCCGC- TCCTTTGTGT ---AGTGCTC AYCCA---GG
Rattus rattus             ---------- ---------- ---------- ---------- ---------- ----------
Rattus exulans            ---------- ---------- ---------- ---------- ---------- ----------
Rattus_norvegicus         GAGACCTCAC GACTGACTTT TCTTCCCGC- TCCTTTGTGT ---AGTGTTC ACCCA---GG
Mus_musculus              GAGACCTCAA GACTGACTTT TCTTCCCGC- TCCTTTGTGT ---AGTGCTC ACCCA---GG
Mus_spretus               ---------- ---------- ---------- ---------- ---------- ----------
Mus_pahari                ---------- ---------- ---------- ---------- ---------- ----------
Praomys                   GAGGCCTCAG GACTGGCTTT TCTTCCCGC- TCCTTTGTGT ---AGCGCTC ACTCA---GG
Meriones                  GAGACCTTGC GACTGGC-TT TCTTCCTGC- TCCTT-GTGC ---AGTC--- ----------
Meriones_crassus          ---------- ---------- ---------- ---------- ---------- ----------
Acomys cahirinus          GAGACCTTGC GACCGGGGTT TCTTCTCGC- TCCTTTGTGT ---AGTGCTC AGCCA---GG
Lophuromys sikapusi       GAGACCTTGT GACTGGCTTT TCTTCCCAC- TCCTTTGTGT ---AGTGCTC AGCCA---GG
Mesocricetus_auratus      AAGACCTTGT GACTGGCTTT TCTTCCCGA- TTCTTTGTGT ---AGCACTC ACCAA---GG
Oryzomys                  GAGACTTTCC GACTGGTTTT TCTTCCTGC- TCCTTTGTGT ---AGAACTC ACCCA---GG
Trichys_fasciculata       ----TGT-GC GGAGTGA--T CCTTCTCGC- TCCTTTGTGT ---AACTCTC GCCCT---GG
Heterocephalus_glaber     CGTGTGC--- AGAGTGA--T SCTTCTCTC- TCCTTTGTGT ---AACTCTC ACCCC---GG
Coendou_melanurus         CGGGTGC--- AGAGTGA-TC CCT-CTCGC- TCCTTTGTGT ---AGCTCTC ACC-T---GG
Cavia_porcellus           CATGTGT--- ---GGA---T CCTTCTCGC- TCCTTTGTGT ---AACTCTC ACCCT---TA
Ctenomys_maulinus         CGTGTGC--- AGAGCGA--T CCTTCCCGC- TCCTTTGTGT ---AACTCTC ACCCT---GG
Octodon_degus             CGTGTGC--- AGAGCGA--T CCTTCCCGC- TCCTTTGTGT ---AACTCTC ACCCT---GG
Loxodonta_africana        CATGCAC--C AGGCATCTTC TTTTCCACC- CTCTTTGTGT ---AGCACTT ACCCT---GG
Procavia_capensis         TGTATAC--C AGGCATCGTC TTTTCCCCT- CTCTTTGTGC ---AACGCTT ACCCT---GG
Echinops_telfairi         TTTCCTTTTC CCAAAGTTCC CCTCAATGTG ATAGTTATCC ---ACCACCG GGCTT---GG
Micropotamogale           CGTGCAC--C AGACATCTTA TGTTCTGCT- TTCTTTGAAC ---ACCGCGT ACCTT---GC
Myotis_lucifugus          GGTGGGCCTC TAGTCCTGTC CTTGCTTTAA AATACTGTGT -------TTA GCCCTGGCCG
Canis_familiaris          TGTCTAT--C AAACGTCCCC TTTCCGCTC- CC-TTTGGGG ---GCACCTG GCCCT---GG
Felis_catus               AGTCCGT--G TCTCCCTCGC TCTCTGCTC- CT-CCCCCGC ---TCTCGGT CTCTC---AG
Ovis_aries                ---------- ---------- ---------- ---------- ---------- ----------
Equus_caballus            ----TAT--C CAGCGTCTTC TTTTCACCG- CT--TTGCGT ---AGCACTT ACCCT---GG
Tapirus_terrestris        ---------- ---------- ---------- ---------- ---------- ----------
Vicugna                   TGTGTGT--C AGGCGTCCTC TATTCACTC- CC-TTTGTGG ---AATCCAC CACA------
Sus_scrofa                TGCGTAT--C AGGCTTCCTC TCTTCACTC- CC-TTTGTGC ---TGCGCTT ACCCT---GG
Bos_taurus                CGTGTAT--C AGGC---TTC TTTTCACTC- CT-TTTGTGT ---TGCACTT AACCT---GG
Tursiops_truncatus        TGTGTGT--C -AGCTTCCTC TTTTCACTC- CC-TTTGTGT ---TGTAAGT NNNNN---NN
Erinaceus                 CGTGTGTCCC AGACATCCTC TCTGCACCCT CCCTTTGTGC CGGAGCACTC ACCCT---GG
Sorex_araneus
Neomys_anomalus           ---------- -------
Choloepus_hoffmanni       ---------- ---------- ---------- ---------- ---------- ----------
Dasypus                   CGTGTGTACC GGGCATCTTT CCTCCACTC- CC-TCCGTGT ---A---CTC ACCCC---GG
```

```
                          3481
Monodelphis               AGGTCAGGTA ATGACTAATG CAAAAAAGAA CAAAAAATCA A
Macropus                  AGGTCAGGTA ATGACTAATG CAAAAAAGAA -AAAAAGTCA A
Microcebus_murinus        -AGATATAGA TTACTTAAAA CTNNN----- ---------- -
Tarsius_syrichta          GGTGTCGTGC AGTAACGACG GCCAA----- --GAAAGAT- --
Callithrix_jacchus        AGGT------ ----CTAATG CCAAA----- -GAAAAATCA A
Macaca_mulatta            AGGTCATATA GTAACTAATG CCAAA----- -GAAAAATCA A
Pongo_pygmaeus            AGGTAATGTA GTAACTAAGG CCAAA----- -GAAAAATCA A
Gorilla_gorilla           ---------- ---------- ---------- ----------
Homo_sapiens              AGGTCATGTA GTAACTAATG CCAAA----- -GAAAAATCA A
Pan_troglodytes           AGGTCATGTA GTAACTAATG CCAAA----- -GGAAAATCA A
Ochotona_princeps         AGGTCATGTA ATAACTAATA CCAAA----- -GAACAATCT A
Oryctolagus_cuniculus     AGGTCATGTA ATAACTAATG CCAAA----- -GAACAATCC A
Marmota_monax             A--------- ---------- ---------- ---------- -
Aplodantia_rufa           ---------- ---------- ---------- ---------- -
Spermophilus              ---------- ---------- ---------- ----------
Anomamorus                ---------- ---------- ---------- ----------
Maxomys                   A--------- ---------- ---------- ---------- -
Rattus rattus             ---------- ---------- ---------- ---------- -
Rattus exulans            ---------- ---------- ---------- ---------- -
Rattus_norvegicus         AGGTCAGGCA GTAACTAATG CCAAA----- -GAAACATCA A
Mus_musculus              AGGTCAGGCA GTAACTAATG CCAAA----- -GAAACATCA A
Mus_spretus               ---------- ---------- ---------- ---------- -
Mus_pahari                ---------- ---------- ---------- ---------- -
Praomys                   A--------- ---------- ---------- ---------- -
Meriones                  ---------- ---------- ---------- ---------- -
Meriones_crassus          ---------- ---------- ---------- ---------- -
Acomys cahirinus          A--------- ---------- ---------- ---------- -
Lophuromys sikapusi       A--------- ---------- ---------- ---------- -
Mesocricetus_auratus      A--------- ---------- ---------- ---------- -
Oryzomys                  A--------- ---------- ---------- ---------- -
Trichys_fasciculata       A--------- ---------- ---------- ---------- -
Heterocephalus_glaber     A--------- ---------- ---------- ---------- -
Coendou_melanurus         A--------- ---------- ---------- ---------- -
Cavia_porcellus           AGGTCAGGCA GTAACTAAAG CCAAA----- -GAAACATCA A
Ctenomys_maulinus         A--------- ---------- ---------- ---------- -
Octodon_degus             A--------- ---------- ---------- ---------- -
Loxodonta_africana        AGGTCATGTA ATAACTAATG CCAGA----- -GAAAAATCA A
Procavia_capensis         AGGTCATGTA ATAACTAATG CCAGG----- -GAAAAATCA A
Echinops_telfairi         ACCTCTTACT CCATTCACCC AGGGCCCTGG TAACTGACCT GCAAT
Micropotamogale           A
Myotis_lucifugus          GTTTGGCTCA GTGGATAGAG ---------- ---------- -
Canis_familiaris          AGGTCATGCA GTTACGAGTG CCGCA----- -GAGAAATCC G
Felis_catus               A----ATGT- --GAATAAAA CGGT------ ---------- -
Ovis_aries                ---------- ---------- ---------- ----------
Equus_caballus            AGGTCAAGTA GTAACCAATG CCAAA----- -GA-AAATCA A
Tapirus_terrestris        ---------- ---------- ---------- ---------- -
Vicugna                   ---------- ---------- ---------- ---------- --
Sus_scrofa                AGGTCATGTA ATAACCAATG CCAAA----- -GAAAAATCA A
Bos_taurus                AGGTCACGCA ATAACCAATG CCAAA----- -GAAAAATCA A
Tursiops_truncatus        NNNNNNNNNN NNNNNNNNNN NNNNN----- -NNNNNNNNN N
Erinaceus                 AGGTCACGTA ACAACTAATG GCAAA----- -GC-AAATCA A
Sorex_araneus
Neomys_anomalus
Choloepus_hoffmanni       --NNNNNGTA GTAACTAATG CCAAA----- -GAAAAATCA A
Dasypus                   AGGTCATGTA GCAGCGAAT- ---------- ---------- -
```
